# Mask decontamination methods (model N95) for respiratory protection: a rapid review

**DOI:** 10.1186/s13643-021-01742-1

**Published:** 2021-08-07

**Authors:** Livia Fernandes Probst, Ana Tereza Gomes Guerrero, Andréia Insabralde de Queiroz Cardoso, Antonio Jose Grande, Mariana Garcia Croda, James Venturini, Maria Cristina de Camargo Fonseca, Anamaria Mello Miranda Paniago, Jorge Otávio Maia Barreto, Sandra Maria do Vale Leone de Oliveira

**Affiliations:** 1 Piracicaba Dental School, State University of Campinas, Piracicaba, Brazil; 2 Health Technology Assessment Unit, Hospital Alemão Oswaldo Cruz, São Paulo, Brazil; 3 Institute of Technology in Immunobiologicals: Bio-Manguinhos. Oswaldo Cruz Foundation, Rio de Janeiro, Brazil; 4 Federal University of Mato Grosso do Sul , Campo Grande, Brazil; 5 Faculty of Medicine , State University of Mato Grosso do Sul, Campo Grande, Brazil; 6 State University of Feira de Santana, Feira de Santana, Brazil; 7 Fiocruz School of Government Fiocruz Brasília, Oswaldo Cruz Foundation, Brasília, Brazil

**Keywords:** Decontamination, Disinfection, Sterilization, Equipment reuse, N95 respirator, COVID-19

## Abstract

**Background:**

N95 respiratory protection masks are used by healthcare professionals to prevent contamination from infectious microorganisms transmitted by droplets or aerosols.

**Methods:**

We conducted a rapid review of the literature analyzing the effectiveness of decontamination methods for mask reuse. The database searches were carried out up to September 2020. The systematic review was conducted in a way which simplified the stages of a complete systematic review, due to the worldwide necessity for reliable fast evidences on this matter.

**Results:**

A total of 563 articles were retrieved of which 48 laboratory-based studies were selected. Fifteen decontamination methods were included in the studies. A total of 19 laboratory studies used hydrogen peroxide, 21 studies used ultraviolet germicidal irradiation, 4 studies used ethylene oxide, 11 studies used dry heat, 9 studies used moist heat, 5 studies used ethanol, two studies used isopropanol solution, 11 studies used microwave oven, 10 studies used sodium hypochlorite, 7 studies used autoclave, 3 studies used an electric rice cooker, 1 study used cleaning wipes, 1 study used bar soap, 1 study used water, 1 study used multi-purpose high-level disinfection cabinet, and another 1 study used chlorine dioxide. Five methods that are promising are as follows: hydrogen peroxide vapor, ultraviolet irradiation, dry heat, wet heat/pasteurization, and microwave ovens.

**Conclusions:**

We have presented the best available evidence on mask decontamination; nevertheless, its applicability is limited due to few studies on the topic and the lack of studies on real environments.

**Supplementary Information:**

The online version contains supplementary material available at 10.1186/s13643-021-01742-1.

## Background

Severe acute respiratory syndrome coronavirus 2 (SARS-CoV-2), which is responsible for the coronavirus pandemic (COVID-19), has affected the world and changed the way we live [1–5]. Aerosols, droplets, secretions, and direct contact with nasal mucosa are the main respiratory transmission routes of these viruses between humans [6].

Health professionals have been using respiratory protection masks; particularly model N95, in the care of infected patients for aerosol-generating procedures (tracheal intubation, non-invasive ventilation, tracheotomy, cardiopulmonary resuscitation, manual ventilation, before intubation, collections of nasotracheal secretions, and bronchoscopies). The recommendation is to discard them after close contact with a patient (single-use) [7–9].

During the current COVID-19 pandemic, governments have found it difficult to acquire adequate amounts of personal protection equipment (PPE), including respiratory protection masks. This has been accompanied by a high level of infection of health professionals on the front lines of care provided for sick people [10–14]. The COVID-19 pandemic has not only exposed how crucial effective PPE is for health professionals, but also exposed the inability of health systems to meet this demand. In this context, based on the shortage of N95 masks, one of the alternatives found was the decontamination and reuse of PPE [15].

Even though research studies have been conducted on how decontamination may be conducted, there is concern about disease transmission as there are no best practices for mask decontamination and reuse [16]. In such critical circumstances, as we go through, rapid analyses are recommended by WHO [17] to provide guidance for timely decision making. Therefore, this rapid review aims to describe the effectiveness and safety of decontamination methods for N95 masks for reuse as protection against COVID-19.

Recent systematic reviews evaluated the efficacy and safety of different mask decontamination methods (model N95) [18, 19]. Other reviews were carried out evaluating specific methods of decontamination, such as microwave and heat [20], ultraviolet germicidal irradiation [21], and chemical disinfectants [22]. Concerns about efficacy and safety of methods of decontamination of personal protective equipment are, and will continue to be, relevant for scientific evidence as there has been a likelihood for the third wave of COVID-19 recently. Thus, planning for stockpiling and handling of personal protective equipment should be one of the priorities [23, 24].

This rapid review adds knowledge to evidence-based decision making as it presents a global view of 15 different decontamination methods reported by 48 original research articles. It is possible to assume from our results that affordable methods are available such as the microwave oven, to methods more expensive choices such as ultraviolet germicidal irradiation (UVGI). In addition, this review highlighted that the current evidence is insufficient to determine a safe and widely accessible method, despite all recent efforts in this field of research. It is important to mention that our first search in April 15 recovered less than half of the studies than the updated search. In addition, of the 48 studies included, 31 were published after April 2020, reinforcing the growth of primary research in the area.

## Methods

This rapid review follows the recommendations outlined by Haby et al. [25]. A rapid review is a form to provide a knowledge synthesis through streamlining or omitting specific methods to produce evidence for stakeholders more quickly [26]. Therefore, based on the recommended approaches of a rapid review [25], only the risk of bias assessment of the included studies was not carried out. Challenging scenarios such as the onset of coronavirus demand that decision makers receive the best evidence quickly and urgently, making traditional methods of systematic review unviable [27].

### Criteria for considering studies for this review

Based on these recommended approaches [25], we developed a specific protocol for this study (Supplementary material 1). We planned to include only primary research studies that evaluated methods for the decontamination of N95 masks for reuse and whose outcome was the effectiveness, safety, maintenance of protection, or filtering characteristics of the evaluated decontamination method were included. Therefore, we could answer the question: “How effective and safe are decontamination methods for respiratory protection masks model N95/PFF2 against respiratory viruses?”

### Information sources for identification of studies

We followed the limit main database searching recommended by Cochrane Rapid Reviews Methods Group [27]. Therefore, searches were conducted on MEDLINE, Cochrane Library, and EMBASE databases on September 25, 2020. Search terms were related to decontamination (e.g., “Sterilization,” “Disinfection,” and “Decontamination”), reuse (e.g., “Equipment Reuse” and “Reuse”), device failure (“Equipment Failure”), and masks (e.g., “N95” and “filtering facepiece respirators”).

The search strategy was developed in two stages. In the first stage, an experienced researcher (LFP) structured the strategies with the collaboration of different specialists (AIQC, ATGG, JV, MCCF, AMMP, JOB, and SMVLO). In the second stage, a researcher (AJG) who is trained by the Cochrane Systematic Reviews Group evaluated and validated the search strategies. The review did not have a date or language restrictions. The complete search strategies can be found in Table 1 of Supplementary material 2; therefore, they are valid, reliable, and reproducible.

### Searching other resources

In addition to searching the official databases, the reference lists of all studies selected for full-text reading, as well as the review articles identified in official searches, were scrutinized to identify possible eligible studies.

### Selection process

Two reviewers (LFP and AIQC) independently screened for the title, abstracts, and full text, the reviewers used the Rayyan systematic review application in blind mode [28]. Disagreements during the selection process were resolved by discussion with a third review (SMLVO). Mendeley citation management software was used for the automatic removal of duplicate articles.

### Data collection process and analysis

Two reviewers (LFP and AIQC) independently used a pre-specified data extraction sheet form, in duplicates, designed to obtain the specific data required for this review. The data extracted from the primary studies were data related to the author, year, study objective, intervention, comparator, commercial mask model, target microorganism, results, and conclusions of the study authors, limitations, and detailed description of the decontamination process making its reproducibility in other scenarios possible. Moreover, authors of the included primary studies were also contacted to provide data that was not available in the manuscripts.

### Data synthesis

The characteristics of each study (cycles, temperatures, protocols, densities, exposure time, technology used, and results) are presented in Table 3 of Supplementary material 4 [29–76]. The differences before and after decontamination are shown in Table 4 of Supplementary Material 5 for outcomes: filter aerosol penetration, filter airflow resistance, and filtration efficiency. Results of the studies were summarized based on the decontamination method and results for the two following issues [53]:

1. Whether the device maintains its structural characteristics and provides an adequate level of protection after the decontamination method, without any risk of exposure for the health professional (as inhalable chemical residues that may have remained after the method used). The penetration of 0.3 μm (aerodynamic mass mean diameter) of sodium chloride aerosols aerosol particles through a certified N95 respirator cannot exceed 5% [77]. Also inhalation and exhalation resistance to airflow of certified N95, i.e., filter airflow resistance utilizing a filter tester at 85 l/min of constant airflow, shall not exceed 35mm (343.2 Pa) water column height pressure and upon initial exhalation shall not exceed 25mm (245.1 Pa) water column height pressure. These are specifications required for certification as a 95% filtration efficiency level [78].
2. Whether the decontamination method used was effective in reducing or eliminating the infectious capacity of the target organism without any risk of exposure for the health professional to contamination. This criterion can be verified when the mean log reduction of the microorganisms allows us to state that the mask has reached non-infectious levels, as recommended by the FDA, at level 1 with a reduction (≥6-log) of more resistant spores and Mycobacterium. Alternatively, using a quantitative molecular amplification assay (quantitative real-time polymerase chain reaction) that shows if there was a reduction in the levels of detectable viral RNA, with the absence of any pathogenic infectious agent [53].

## Results

### Search results

The complete search strategies can be found in Table 1 of Supplementary material 2. Initial searches retrieved 552 articles (MEDLINE: 381, Cochrane: 52, and EMBASE: 119). The reference lists of all studies selected for full-text reading, as well as the review articles identified in official searches, were scrutinized to identify possible eligible studies. Authors of the included primary studies were also contacted. These manual searches detected 11 additional publications that were added to the total recovered. Mendeley citation management software was used for the automatic removal of duplicate articles, leaving 301 studies remaining.

### Selection process

**Fig. 1:**
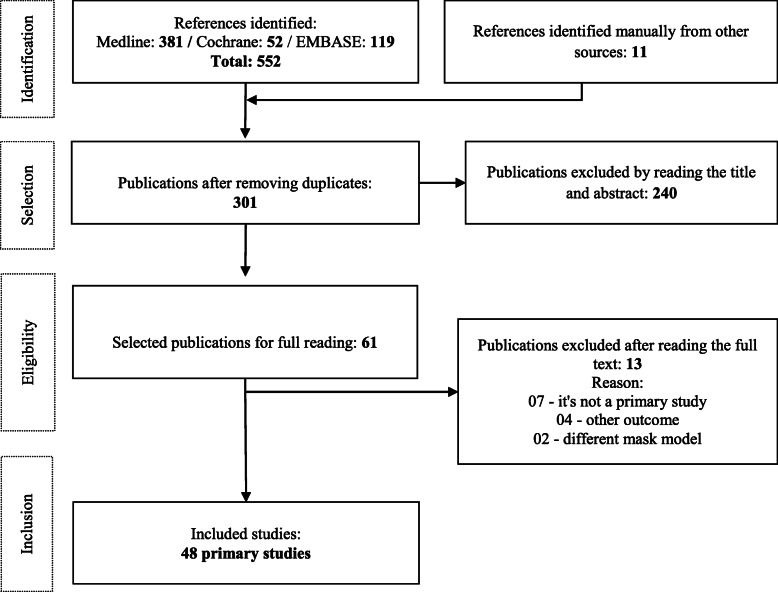
PRISMA flow-chart of the study selection process

Two reviewers (LFP and AIQC) independently screened the 301 studies using the Rayyan systematic review application to screen abstracts and titles [28]. Of these, 240 were excluded for not meeting the inclusion criteria. The full texts of 61 studies were screened by two reviewers (LFP and AIQC), and thirteen additional studies were excluded. Excluded full-text studies and the reasons for exclusion are listed in Table 2 of Supplementary material 3 [79–91]. At the end, 48 studies were selected for the full-review process (Fig. 1).

Fifteen methods were assessed in the 48 papers: hydrogen peroxide, ultraviolet irradiation, ethylene oxide, dry heat, moist heat/pasteurization, ethanol, isopropanol solution, microwaving, sodium hypochlorite (NaClO), autoclaving, electric rice cooker, cleaning wipes, bar soap, and water, multi-purpose high-level disinfection cabinet (Altapure, Mequon, WI), and chlorine dioxide (ClO_2_) [29–76]. Each method will be briefly analyzed below:

### Hydrogen peroxide

Hydrogen peroxide was evaluated in its liquid, plasma, and gas/vapor forms by nineteen laboratory studies [29–47]. The effect of hydrogen peroxide on the filtering capacity varied according to the method used. The average penetration was not significantly changed when the masks were submerged in 3% or 6% hydrogen peroxide liquid or treated with vaporized hydrogen peroxide (STERRAD®) for one cycle [29]. However, it resulted in mean penetration levels > 5% after 3-Cycles [32]. The integrity and filtering capacity of the mask were preserved when hydrogen peroxide was used as steam [32, 34, 92].

Additionally, studies have shown hydrogen peroxide led to changes in the masks’ metallic nasal clips [29, 30, 32]. As for the ability to eliminate microorganisms, SteraMist™ Binary Ionization Technology® (BIT™) was effective against influenza A virus subtype H1N1 [33] and *Geobacillus stearothermophilus spores* [43] and STERRAD 100NX sterilization system eliminated *SARS-CoV-2*, *Staphylococcus aureus*, and *Acinetobacter baumannii* [44].

On the other hand, vaporized hydrogen peroxide (VHP) was effective in eliminating SARS-CoV-2 [34], *Geobacillus stearothermophilus* spores [35], *Porcine respiratory coronavirus* (PRCV) [39], *Escherichia coli, Mycobacterium smegmatis*, and *spores of Bacillus stearothermophilus* [45] and 3 aerosolized bacteriophages: T1, T7, and Pseudomonas phage phi-6 [47].

### Ultraviolet germicidal irradiation (UVGI)

The effect of ultraviolet germicidal irradiation on N95 respirator masks was evaluated by 21 studies [29–32, 34, 39, 42, 48–61], and there was a difference among studies in relation to UVGI doses and application time periods. In general, UVGI did not affect the integrity and ability of the masks to filter aerosols or adapt to the face, nor did it leave a smell, irritating/toxic residues, or create important changes in appearance even when multiple cycles were performed [29–32, 34, 42, 49, 51–53, 55, 57, 58]. However, different commercial brands of N95 models resisted differently in terms of performance penetration after multiple cycles and doses applied [55]. The most efficient N95 brand in terms of long-standing penetration performance was 3M 9210, but the mean penetration values for all brands were 5% or less both before and after exposure [55]. The most efficient N95 brand in terms of long-standing flow resistance performance was 3M 9210, but the mean flow resistance values were less than 1% of the initial value for all brands [55].

Ultraviolet germicidal irradiation was effective against MS2 coliphage (ATCC 15597-B1) [48], the influenza virus H5N1 [53] SARS-CoV-2 [34, 60, 61], bacteriophages MS2 [49, 54], influenza virus H1N1 [50, 56] and porcine respiratory coronavirus (PRCV) [39]. However, at the same time, one study reported that even after 20 min of irradiation with 365 nm UVA the relative survival of *Bacillus subtilis* spores remained above 20% [57] and another one highlighted the UV-C technologies tested did not meet pre-established criteria for decontamination against *Methicillin-resistant Staphylococcus aureus* (MRSA), *Bacteriophage* Phi6, and *Bacteriophage* MS2 [59].

### Ethylene oxide

Evaluated by four studies, the effectiveness of ethylene oxide (EtO) depended on the type of sterilization equipment used, whether there was a hot cycle, and exposure to EtO [29–32]. The process did not affect the filtration, resistance, odor, or appearance of the masks. The main limitations of the method were the processing time and the presence of toxic residues. It is also important to note that none of the studies tested the effectiveness of EtO treatment against microorganisms. Thus, there is no evidence that EtO can eliminate any microorganism from N95 masks.

### Dry heat

The use of dry heat was evaluated by eleven different experimental studies [29, 30, 34, 39, 58, 59, 62–66]. Temperatures between 70 and 85°C did not affect the structural characteristics of the masks under various humidity conditions (≤100% RH) [34, 58]. Also, filtering efficiency remained acceptable (≥95%) up to 50 cycles at 85°C and 30% of RH [58]. When an oven was used at 70°C, filtration performance was maintained if only one cycle was performed [34]. However, there were no detectable changes in aerosol filtration efficiency, even after three reprocessing cycles when the masks were subjected to the same temperature of 70°C when laboratory MINI/6 incubator (Genlab Ltd.) was used to provide dry heat [63].

Dry heat treatment (70°C for 60 min) was effective against SARS-CoV-2 [34, 63]; however, dry heat (70°C for 30 min) had limited effectiveness against bacteriophages MS2 and Phi6 versus methicillin-resistant Staphylococcus [59]. Also, dry heat at 100°C for 15 min did not eliminate *Methicillin-resistant Staphylococcus aureus* (*MRSA*) and *Nonenveloped single-stranded RNA virus bacteriophage MS2* [64].

### Moist heat/pasteurization

Nine studies [32, 50–53, 58, 63, 67, 68] evaluated the effect of moist heat between 60 and 100°C. The method did not alter mask fit, odor, or comfort [32, 50, 51, 53, 58]. In one study [58], filtration efficiency presented a significant drop after cycle 5 when stacked on top of a beaker with boiling water inside (around 15 cm above the water), although filtration efficiency was not affected when masks were subjected to 5 cycles with a lower temperature—at 85°C + 60–85% humidity [68]. Moist heat (65±5°C for 3h) was effective in eliminating H1N1 [50] and H5N1 [53] viruses; in addition, when masks were steamed in boiling water, the treatment was effective against the Avian infectious bronchitis virus H120 without changing [67]. Another study also has shown that a single-heat treatment for thermal disinfection in cycles of 60 min at 70°C at 50% eliminated *SARS-CoV-2* and *Escherichia coli* [63].

### Ethanol

Different methods of decontamination with ethanol were tested: spray [34], immersion [58, 69, 70], and pipette drips [57]. Results were divergent between methods. The filtration efficiency of masks was degraded to unacceptable levels when they were immersed in alcohol [58, 69, 70]. Mask filtration performance was not significantly reduced after single ethanol sprays which were also effective in eliminating SARS-CoV-2 [34]. Subsequent rounds of spraying caused a significant decrease in efficacy in filtration performance [34]. Nonetheless, pipette drips were not effective in eliminating *Bacillus subtilis* spores [57].

### Isopropanol solution

The filtering capacity of N95 masks was changed and the particle penetration through N95 masks exceeded 5% after they had been submerged in isopropanol solution [29, 69]. Although effects on microorganisms were not evaluated, this method could be further studied in order to check these effects.

### Microwave oven

Eleven studies tested the use of microwave ovens in the disinfection of N95 masks [29, 30, 32, 49–53, 62, 71, 72]. The type of commercial furnace, maximum temperature, and time protocols varied between the studies (Table 3, Supplementary material 4). When masks were placed directly on the rotating plate of the microwave without protection, two commercial models of the tested masks melted [30]. When the masks were placed in containers with water [32, 50, 51, 53, 72] or in steam bags specifically marketed for microwave ovens no residual odor was observed [49]. In addition, there were no structural changes affecting adjustment on the face, filtration capacity, or resistance to airflow and none of the metal components melted or combusted. Microwaving the masks was effective in eliminating H5N1 [30] and H1N1 influenza viruses [51], bacteriophage MS2 [49, 72], and *Staphylococcus aureus* [62].

### Sodium hypochlorite (NaClO)

Ten studies [29–32, 42, 48, 57, 58, 69, 71] evaluated the use of hypochlorite at different concentrations and application methods (Table 3, Supplementary material 4). The maintenance of mask integrity and filtering capacity varied among studies. For instance, one round of disinfection drastically degraded filtration efficiency to unacceptable levels when samples were left to air dry and off-gas completely, hanging [58], or submersed in 0.5% sodium hypochlorite for 10 min [69]. Application of sodium hypochlorite discolored the metallic components of the masks which, unfortunately, caused a characteristic smell of bleach [30].

One of the study treatments caused the release of low levels of hydrochloric gas [31]. Hypochlorite concentrations of 0.006%, 0.06%, and of 0.6% were not effective against Coliphage MS2 [71]. On the other hand, when higher sodium hypochlorite doses (>8.25 mg/liter) were used for the dilute solutions containing 2.75 to 5.50 mg/liter during a 10-min decontamination period MS2 coliphage was inactivated [48]. When 5.4%, 2.7%, or 0.54% NaOCl was used, the method was effective in eliminating *Bacillus subtilis* spores [57].

### Autoclave

Considered an accessible method, as it is equipment present in all hospital environments, autoclave decontamination was evaluated by 7 studies [29, 57, 69, 70, 73–75]. Autoclave disinfection was effective in eliminating *Bacillus subtilis* spores [57]; however, the integrity of the mask was altered [29, 69, 75]. Two studies pointed that even though masks were able to retain their structural integrity and efficacy, no results were provided regarding the filtering capacity, and only the fittest was performed [73, 74]. In addition, a survey conducted in 2020 showed the degree of integrity of the mask can be preserved depending on the model tested (Table 3, Supplementary material 4) [70].

### Electric rice cooker

The method showed 99–100% biocide efficacy against *Bacillus subtilis* [57] spores after using dry heat for 3 min (149–164°C, without adding water). Also, the treatment for 13–15 min, including 8–10 min of heating and 5 min of steam, resulted in a greater than 5 log_10_ reduction in bacteriophage MS2 and methicillin-resistant *S. aureus* [64]. However, the method visibly changes the mask’s integrity [69].

### Cleaning wipes

The effectiveness of commercial wipes containing 0.9% hypochlorite, benzalkonium chloride, or no active antimicrobial ingredients was evaluated in masks contaminated with *Staphylococcus aureus* and mucin [76]. The three mask models, 3M-1860S, 3M-1870, and Kimberly-Clark-46727-PFR, withstood handling and abrasion during the disinfection process. All were successfully decontaminated against atypically high microbe levels by wipes containing antimicrobial agents; however, inert wipes did not produce adequate decontamination.

### Bar soap and water

Average penetration has markedly increased for N95 respirators after being submerged. Authors have hypothesized the soap could have removed the charge on the fibers similar to the effect observed with isopropanol solution exposure [29].

### Multi-purpose high-level disinfection cabinet (Altapure, Mequon, WI)

The treatment was effective against microorganisms, and the researchers reported no visible changes in the masks. However, the efficiency of the filtration has not been confirmed [59].

### Chlorine dioxide (ClO_2_)

The method significantly changed the filtering efficiency of the tested masks which makes this method not worth using or testing in further studies [36].

## Discussion

Fifteen decontamination methods were identified in the 48 studies [29–76] included in this review. Ten methods have addressed the infectious capacity of microorganisms, and all fifteen methods have investigated masks structural integrity after the decontamination process, also being based on laboratory tests.

As different methodologies were used in each study, summarizing the results is a challenging task. Nevertheless, this review has uncovered the inadequacy of the evidence supporting the use of N95 mask decontamination methods. Therefore, it is important to highlight that even when a mask has retained its structural integrity after decontamination, if the elimination of an organism’s infectious capacity has not been proven, it is still a potential vehicle of transmission. This concern is reinforced by a recent study which showed that pathogens may be present on the external surface of about 10% of used masks, and that the risk of infecting the user increases with prolonged use [93], as the number of viral particles and their survival time is key determining factors when consideration of reuse becomes necessary [83].

The most valuable data for decision making is to identify after which methods the filtration efficiency remained unchanged or within the established thresholds [77]. Although hydrogen peroxide vapor, germicidal ultraviolet irradiation, dry heat ≤85°C, moist heat/pasteurization, and microwaving seems not only promising but also has demonstrated these methods have preserved the integrity of the masks, so far, the evidence does not indicate a method that is consistently safe and effective to decontaminate N95 respiratory protection masks.

Particularly, hydrogen peroxide vapor and ultraviolet germicidal irradiation require specific equipment and environments increasing costs, which should be taken into account. Dry heat, wet heat/pasteurization, and microwave ovens also showed positive results in terms of effectiveness and safety and are more accessible methods in scenarios of scarce financial resources. These results concerning dry heat reinstate that decontamination attempts must be conducted in identical methods and conditions to the ones performed in the laboratory. Not only regarding the temperature, but also regarding the device used. Nevertheless, the effect against different microorganisms varied greatly between different studies due to the decontamination cycle time. The feasibility of these methods should be assessed with new studies, especially by health systems in developing countries.

The decontamination protocols that used hydrogen peroxide (solution and plasma), ethylene oxide, ethanol, sodium hypochlorite, autoclaves, electric rice cooker, and isopropanol solution interfered with the integrity of the masks within the test conditions used in the studies. Commercial cleaning wipes were effective antimicrobial agents and did not degrade the masks; however, they were evaluated by a single study and for only two microorganisms.

In the case of serious emerging infections such as COVID-19, the principle of universal precaution must be considered, that is, a balance must be made between the benefits and risks of the possible decontamination methods, in order to ensure maximum safety and real protection for the user, especially in the case of health professionals on the front line [15, 94].

### Limitations and strengths of this review

As reported in the results, the search strategy was developed by an experienced researcher and validated by another systematic review researcher, who is trained by the Cochrane Systematic Reviews Group. However, it was not developed by a health information specialist. Considering that this was the review that found the largest number of studies that were in fact eligible and included compared to previous reviews which investigated different mask decontamination methods (model N95) [18, 19], we do not consider this a limitation. As a rapid review, another limitation concerns a series of methodological simplifications adopted, which may affect the findings and our interpretations. Eliminating the evaluation of the studies’ methodological qualities was among the simplifications and calls for caution in interpreting the results presented. The technical aspects of outcome assessment in the included studies were not taken into account in this review and is an important limitation when interpreting the presented results.

The strengths of this review, however, are the number of identified decontamination methods and its unfolding results which points to practices that may be adopted and further studied as they seem to present better results initially, the multiple approaches used to search for relevant studies, such as contact with various authors and manual search of article references, and the participation of a team of multi-disciplinary specialists in all stages of the project, which included professionals from the areas: nursing, dentistry, medicine, pharmacy, and physical therapist.

### Agreements and disagreements with other reviews

A previous systematic review that included 15 studies concluded that future studies were required in order to establish the efficacy and safety of N95 decontamination methods [18]. Other systematic reviews have reported that masks can be decontaminated with microwave irradiation and moderate-temperature heat (up to 90°C), in both moist and dry conditions [20] and a single cycle of vaporized H_2_O_2_ can be used as a chemical disinfectant to remove viral pathogens without degrading the masks [22]. Our review is broader than the cited ones, which shows that our search strategy is much more sensitive. However, there is not sufficient evidence concerning UVGI as a safe decontamination method [21] To our knowledge, this is the first rapid review to assess 15 different decontamination methods identified in 48 studies, providing an overview of all available methods.

### Implications for future research studies

Whereas the current evidence is insufficient to determine a safe and widely accessible method, even for countries with financial limitations, our review points to an important gap in the evidence base, despite recent research efforts. In addition, considering the possibility of new challenging pandemic scenarios, investigating decontamination methods for reuse of protection professional equipment has an environmental, social appeal, and economic aspect. Moreover, it is important to invest in new fabric technologies that are prepared to be reused.

## Conclusion

Access to effective PPE should be guaranteed for health care workers on the front lines of pandemics. However, there is currently insufficient evidence to recommend any method as being safe and effective for the decontamination and reuse of respiratory protection masks. Even though there are several promising methods worth for further studies such as hydrogen peroxide vapor, germicidal ultraviolet irradiation, dry heat at temperatures ≤85°C, wet heat/pasteurization, and the microwave oven, this rapid review has exposed all methods for decontamination need further evaluation and validation in real-life scenarios, also considering economic issues for implementation.

## Supplementary Information

**Additional file 1.** Rapid review protocol.

**Additional file 2: Table S1.** Search strategies and results from each database on September 25, 2020.

**Additional file 3: Table S2.** Studies excluded after full reading and justifications for exclusion.

**Additional file 4: Table S3.** Decontamination protocols for the 48 studies included.

**Additional file 5: Table S4.** Results assessing filter aerosol penetration, airflow resistance and filtration efficiency.

## Data Availability

All data are included in the manuscript.

## Abbreviations

COVID-19: Coronavirus disease
EtO: Ethylene oxide
SARS-CoV-2: Severe acute respiratory syndrome coronavirus 2
PPE: Personal protection equipment
Influenza A: Virus subtype H5N1
Influenza virus A: Subtype H1N1
NaClO: Sodium hypochlorite
UVGI: Ultraviolet germicidal irradiation
UVA: Ultraviolet A
MS2: Bacteriophage
WHO: World Health Organization

## References

[1] Hui DS (2017). Epidemic and emerging coronaviruses (severe acute respiratory syndrome and middle east respiratory syndrome). Clin Chest Med..

[2] Sims LD, Domenech J, Benigno C, Kahn S, Kamata A, Lubroth J (2005). Origin and evolution of highly pathogenic H5N1 avian influenza in Asia. Vet Rec..

[3] Neumann G, Noda T, Kawaoka Y (2009). Emergence and pandemic potential of swine-origin H1N1 influenza virus. Nature..

[4] Guo Y, Cao Q, Hong Z (2020). The origin, transmission and clinical therapies on coronavirus disease 2019 (COVID-19) outbreak – an update on the status. Mil Med Res..

[5] Zambon M (2014). Influenza and other emerging respiratory viruses. Medicine (Baltimore)..

[6] Otter JA, Donskey C, Yezli S, Douthwaite S, Goldenberg SD, Weber DJ (2016). Transmission of SARS and MERS coronaviruses and influenza virus in healthcare settings: the possible role of dry surface contamination. J Hosp Infect..

[7] Long Y, Hu T, Liu L (2020). Effectiveness of N95 respirators versus surgical masks against influenza: a systematic review and meta-analysis. J Evid Based Med.

[8] Bartoszko JJ, Farooqi MAM, Alhazzani W, Loeb M. Medical masks vs N95 respirators for preventing COVID-19 in health care workers a systematic review and meta-analysis of randomized trials. Influenza Other Respi Viruses. 2020;(4):365–3 10.1111/irv.12745. 10.1111/irv.12745 PMC7298295 32246890

[9] Jefferson T, Del Mar CB, Dooley L, et al. Physical interventions to interrupt or reduce the spread of respiratory viruses. Cochrane Database Syst Rev. 2011;7 10.1002/14651858.CD006207.pub4. 10.1002/14651858.CD006207.pub4 PMC6993921 21735402

[10] Zhang Z, Liu S, Xiang M (2020). Protecting healthcare personnel from 2019-nCoV infection risks: lessons and suggestions.

[11] Malhotra N, Gupta N, Ish S, Ish P. COVID-19 in intensive care. Some necessary steps for health care workers. Monaldi Arch Chest Dis. 2020;90(1) 10.4081/monaldi.2020.1284. 10.4081/monaldi.2020.1284 32210421

[12] Xiang YT, Jin Y, Wang Y, Zhang Q, Zhang L, Cheung T (2020). Tribute to health workers in China: a group of respectable population during the outbreak of the COVID-19. Int J Biol Sci..

[13] Willsher K, Borger J, Holmes O. US accused of “modern piracy” after diversion of masks meant for Europe. The Guardian: Julian Borger in Washington and Oliver Holmes in Jerusalem. [online publication]. Available at https://www.theguardian.com/world/2020/apr/03/mask-wars-coronavirus-outbidding-demand. Accessed on 21 June 2021.

[14] Whalen J, Morris L, Hamburger T, McCoy T. White House scrambles to scoop up medical supplies worldwide, angering Canada, Germany. The Washington Post. https://www.washingtonpost.com/business/2020/04/03/white-house-scrambles-scoop-up-medical-supplies-angering-canada-germany/. Published 2020.

[15] Rubio-romero JC, Pardo-ferreira MC, García JAT, Calero-Castro S. Disposable masks: disinfection and sterilization for reuse, and non-certified manufacturing, in the face of shortages during the COVID-19 pandemic. Safety. 2020;(January). 10.1016/j.ssci.2020.104830. 10.1016/j.ssci.2020.104830 PMC7218384 32406406

[16] Chan KH, Yuen K (2020). COVID-19 epidemic: disentangling the re-emerging controversy about medical facemasks from an epidemiological perspective. Int J Epidemiol.

[17] World Health Organisation (WHO). Rapid reviews to strengthen health policy and systems: a practical guide. Andrea C. Tricco EVL and SES, ed. Published online 2017. p. 119. https://www.who.int/alliance-hpsr/resources/publications/rapid-review-guide/en/

[18] Rodriguez-Martinez CE, Sossa-Briceño MP, Cortés JA. Decontamination and reuse of N95 filtering facemask respirators: a systematic review of the literature. Am J Infect Control. Published online July 2020. 10.1016/j.ajic.2020.07.004. 10.1016/j.ajic.2020.07.004 PMC7342027 32652253

[19] Paul D, Gupta A, Maurya AK (2020). Exploring options for reprocessing of N95 filtering facepiece respirators (N95-FFRs) amidst COVID-19 pandemic: a systematic review. Mukherjee A, ed. PLoS One.

[20] Gertsman S, Agarwal A, O’Hearn K, et al. Microwave- and heat-based decontamination of N95 filtering facepiece respirators: a systematic review. J Hosp Infect. Published online August 2020. 10.1016/j.jhin.2020.08.016. 10.1016/j.jhin.2020.08.016 PMC7443086 32841704

[21] Yang H, Hu J, Li P, Zhang C (2020). Ultraviolet germicidal irradiation for filtering facepiece respirators disinfection to facilitate reuse during COVID-19 pandemic: a review. Photodiagnosis Photodyn Ther..

[22] O’Hearn K, Gertsman S, Webster R, et al. Efficacy and safety of disinfectants for decontamination of N95 and SN95 filtering facepiece respirators: a systematic review. J Hosp Infect. Published online August 2020. 10.1016/j.jhin.2020.08.005. 10.1016/j.jhin.2020.08.005 PMC7423630 32800824

[23] Middleton J, Lopes H, Michelson K, Reid J (2020). Planning for a second wave pandemic of COVID-19 and planning for winter. Int J Public Health..

[24] Wise J (2020). COVID-19: risk of second wave is very real, say researchers. BMJ..

[25] Haby MM, Chapman E, Clark R, Barreto J, Reveiz L, Lavis JN (2015). Designing a rapid response program to support evidence-informed decision-making in the Americas region: using the best available evidence and case studies. Implement Sci..

[26] Hamel C, Michaud A, Thuku M, Skidmore B, Stevens A, Nussbaumer-Streit B (2021). Defining rapid reviews: a systematic scoping review and thematic analysis of definitions and defining characteristics of rapid reviews. J Clin Epidemiol..

[27] Garritty C, Gartlehner G, Nussbaumer-Streit B, King VJ, Hamel C, Kamel C (2021). Cochrane Rapid Reviews Methods Group offers evidence-informed guidance to conduct rapid reviews. J Clin Epidemiol..

[28] Ouzzani M, Hammady H, Fedorowicz Z, Elmagarmid A (2016). Rayyan—a web and mobile app for systematic reviews. Syst Rev..

[29] Viscusi DJ, King WP, Shaffer RE (2007). Effect of decontamination on the filtration efficiency of two filtering facepiece respirator models. Int Soc Respir Prot..

[30] Viscusi DJ, Bergman MS, Eimer BC, Shaffer RE (2009). Evaluation of five decontamination methods for filtering facepiece respirators. Ann Occup Hyg..

[31] Salter WB, Kinney K, Wallace WH, Lumley AE, Heimbuch BK, Wander JD (2010). Analysis of residual chemicals on filtering facepiece respirators after decontamination. J Occup Environ Hyg..

[32] Bergman MS, Viscusi DJ, Heimbuch BK, Wander JD, Sambol AR, Shaffer RE (2010). Evaluation of multiple (3-cycle) decontamination processing for filtering facepiece respirators. J Eng Fiber Fabr..

[33] Cheng VCC, Wong S-C, Kwan GSW, Hui W-T, Yuen K-Y (2020). Disinfection of N95 respirators by ionized hydrogen peroxide during pandemic coronavirus disease 2019 (COVID-19) due to SARS-CoV-2. J Hosp Infect..

[34] Fischer RJ, Morris DH, van Doremalen N, Sarchette S, Matson MJ, Bushmaker T (2020). Effectiveness of N95 respirator decontamination and reuse against SARS-CoV-2 virus. Emerg Infect Dis..

[35] Schwartz A, Stiegel M, Greeson N, Vogel A, Thomann W. Decontamination and reuse of N95 respirators with hydrogen peroxide vapor to address worldwide personal protective equipment shortages during the SARS-CoV-2 (COVID-19) pandemic. Appl Biosaf J ABSA Int. 2020;25(2) 10.1177/1535676020919932. 10.1177/1535676020919932 PMC9387741 36035079

[36] Cai C, Floyd EL (2020). Effects of sterilization with hydrogen peroxide and chlorine dioxide solution on the filtration efficiency of N95, KN95, and surgical face masks. JAMA Netw Open..

[37] Lieu A, Mah J, Zanichelli V, Exantus RC, Longtin Y (2020). Impact of extended use and decontamination with vaporized hydrogen peroxide on N95 respirator fit. Am J Infect Control..

[38] Levine C, Grady C, Block T, Hurley H, Russo R, Peixoto B (2021). Use, re-use or discard? Quantitatively defined variance in the functional integrity of N95 respirators following vaporized hydrogen peroxide decontamination during the COVID-19 pandemic. J Hosp Infect..

[39] Ludwig-Begall LF, Wielick C, Dams L, Nauwynck H, Demeuldre PF, Napp A (2020). The use of germicidal ultraviolet light, vaporized hydrogen peroxide and dry heat to decontaminate face masks and filtering respirators contaminated with a SARS-CoV-2 surrogate virus. J Hosp Infect..

[40] Jatta M, Kiefer C, Patolia H, Pan J, Harb C, Marr LC (2021). N95 reprocessing by low temperature sterilization with 59% vaporized hydrogen peroxide during the 2020 COVID-19 pandemic. Am J Infect Control..

[41] Widmer AF, Richner G (2020). Proposal for a EN 149 acceptable reprocessing method for FFP2 respirators in times of severe shortage. Antimicrob Resist Infect Control..

[42] Peltier RE, Wang J, Hollenbeck BL, Lanza J, Furtado RM, Cyr J (2020). Addressing decontaminated respirators: some methods appear to damage mask integrity and protective function. Infect Control Hosp Epidemiol..

[43] Cramer A, Plana D, Yang HL, et al. Analysis of SteraMist ionized hydrogen peroxide technology in the sterilization of N95 respirators and other PPE: a quality improvement study. medRxiv Prepr Serv Heal Sci. Published online April 2020. 10.1101/2020.04.19.20069997. 10.1038/s41598-021-81365-7 PMC7819989 33479334

[44] Ibáñez-Cervantes G, Bravata-Alcántara JC, Nájera-Cortés AS, Meneses-Cruz S, Delgado-Balbuena L, Cruz-Cruz C (2020). Disinfection of N95 masks artificially contaminated with SARS-CoV-2 and ESKAPE bacteria using hydrogen peroxide plasma: impact on the reutilization of disposable devices. Am J Infect Control..

[45] Saini V, Sikri K, Batra SD, Kalra P, Gautam K (2020). Development of a highly effective low-cost vaporized hydrogen peroxide-based method for disinfection of personal protective equipment for their selective reuse during pandemics. Gut Pathog..

[46] Russo R, Levine C, Veilleux C (2021). Decontaminating N95 respirators during the COVID-19 pandemic: simple and practical approaches to increase decontamination capacity, speed, safety and ease of use. J Hosp Infect..

[47] Kenney P, Chan B, Kortright K, Cintron M, Havill N, Russi M (2020). Hydrogen Peroxide Vapor sterilization of N95 respirators for reuse.

[48] Vo E, Rengasamy S, Shaffer R (2009). Development of a test system to evaluate procedures for decontamination of respirators containing viral droplets. Appl Environ Microbiol..

[49] Fisher EM, Shaffer RE (2011). A method to determine the available UV-C dose for the decontamination of filtering facepiece respirators. J Appl Microbiol..

[50] Heimbuch BK, Wallace WH, Kinney K, Lumley AE, Wu CY, Woo MH (2011). A pandemic influenza preparedness study: use of energetic methods to decontaminate filtering facepiece respirators contaminated with h1n1 aerosols and droplets. Am J Infect Control..

[51] Viscusi DJ, Bergman MS, Novak DA, Faulkner KA, Palmiero A, Powell J (2011). Impact of three biological decontamination methods on filtering facepiece respirator fit, odor, comfort, and donning ease. J Occup Environ Hyg..

[52] Bergman MS, Viscusi DJ, Palmiero AJ, Powell JB, Shaffer RE (2011). Impact of three cycles of decontamination treatments on filtering facepiece respirator fit. J Int Soc Respir Prot..

[53] Lore MB, Heimbuch BK, Brown TL, Wander JD, Hinrichs SH (2011). Effectiveness of three decontamination treatments against influenza virus applied to filtering facepiece respirators. Ann Occup Hyg..

[54] Woo M-H, Grippin A, Anwar D, Smith T, Wu C-Y, Wander JD (2012). Effects of relative humidity and spraying medium on UV decontamination of filters loaded with viral aerosols. Appl Environ Microbiol..

[55] Lindsley WG, Martin SB, Thewlis RE (2015). Effects of ultraviolet germicidal irradiation (UVGI) on N95 respirator filtration performance and structural integrity. J Occup Environ Hyg..

[56] Mills D, Harnish DA, Lawrence C, Sandoval-Powers M, Heimbuch BK (2018). Ultraviolet germicidal irradiation of influenza-contaminated N95 filtering facepiece respirators. Am J Infect Control..

[57] Lin T-H, Tang F-C, Hung P-C, Hua Z-C, Lai C-Y (2018). Relative survival of Bacillus subtilis spores loaded on filtering facepiece respirators after five decontamination methods.

[58] Liao L, Xiao W, Zhao M, Yu X, Wang H, Wang Q (2020). Can N95 respirators be reused after disinfection? How many times?. ACS Nano..

[59] Cadnum JL, Li D, Redmond SN, John AR, Pearlmutter B, Donskey C (2020). Effectiveness of ultraviolet-c light and a high-level disinfection cabinet for decontamination of N95 respirators. Pathog Immun..

[60] Ozog DM, Sexton JZ, Narla S, Pretto-Kernahan CD, Mirabelli C, Lim HW (2020). The effect of ultraviolet C radiation against different N95 respirators inoculated with SARS-CoV-2. Int J Infect Dis..

[61] Simmons S, Carrion R, Alfson K (2021). Deactivation of SARS-CoV-2 with pulsed-xenon ultraviolet light: implications for environmental COVID-19 control. Infect Control Hosp Epidemiol..

[62] Pascoe MJ, Robertson A, Crayford A, Durand E, Steer J, Castelli A (2020). Dry heat and microwave-generated steam protocols for the rapid decontamination of respiratory personal protective equipment in response to COVID-19-related shortages. J Hosp Infect..

[63] Daeschler SC, Manson N, Joachim K, Chin AWH, Chan K, Chen PZ (2020). Effect of moist heat reprocessing of N95 respirators on SARS-CoV-2 inactivation and respirator function. Can Med Assoc J..

[64] Li DF, Cadnum JL, Redmond SN, Jones LD, Donskey CJ (2020). It’s not the heat, it’s the humidity: effectiveness of a rice cooker-steamer for decontamination of cloth and surgical face masks and N95 respirators. Am J Infect Control..

[65] Li DF, Cadnum JL, Redmond SN, Jones LD, Pearlmutter B, Haq MF (2020). Steam treatment for rapid decontamination of N95 respirators and medical face masks. Am J Infect Control..

[66] Xiang Y, Song Q, Gu W (2020). Decontamination of surgical face masks and N95 respirators by dry heat pasteurization for one hour at 70°C. Am J Infect Control..

[67] Ma Q-X, Shan H, Zhang C-M, Zhang HL, Li GM, Yang RM (2020). Decontamination of face masks with steam for mask reuse in fighting the pandemic COVID-19: experimental supports. J Med Virol..

[68] Anderegg L, Meisenhelder C, Ngooi CO, Liao L, Xiao W, Chu S (2020). A scalable method of applying heat and humidity for decontamination of N95 respirators during the COVID-19 crisis. PLoS One..

[69] Lin T-H, Chen C-C, Huang S-H, Kuo C-W, Lai C-Y, Lin W-Y (2017). Filter quality of electret masks in filtering 14.6–594 nm aerosol particles: effects of five decontamination methods. Mukherjee A, ed. PLoS One.

[70] Grinshpun SA, Yermakov M, Khodoun M (2020). Autoclave sterilization and ethanol treatment of re-used surgical masks and N95 respirators during COVID-19: impact on their performance and integrity. J Hosp Infect..

[71] Fisher E, Rengasamy S, Viscusi D, Vo E, Shaffer R (2009). Development of a test system to apply virus-containing particles to filtering facepiece respirators for the evaluation of decontamination procedures. Appl Environ Microbiol..

[72] Zulauf KE, Green AB, Nguyen Ba AN (2020). Microwave-generated steam decontamination of N95 respirators utilizing universally accessible materials. mBio.

[73] Carrillo IO, Floyd ACE, Valverde CM, Tingle TN, Zabaneh FR (2020). Immediate-use steam sterilization sterilizes N95 masks without mask damage. Infect Control Hosp Epidemiol..

[74] Czubryt MP, Stecy T, Popke E, et al. N95 mask reuse in a major urban hospital - COVID-19 response process and procedure. J Hosp Infect. Published online July 2020. 10.1016/j.jhin.2020.07.035 10.1016/j.jhin.2020.07.035 PMC7837009 32745590

[75] Harskamp RE, van Straten B, Bouman J, van Maltha-van Santvoort B, van den Dobbelsteen JJ, van der Sijp JRM (2020). Reprocessing filtering facepiece respirators in primary care using medical autoclave: prospective, bench-tobedside, single-centre study. BMJ Open..

[76] Heimbuch BK, Kinney K, Lumley AE, Harnish DA, Bergman M, Wander JD (2014). Cleaning of filtering facepiece respirators contaminated with mucin and Staphylococcus aureus. Am J Infect Control..

[77] Qian Y, Willeke K, Grinshpun SA, Donnelly J, Coffey CC (1998). Performance of N95 respirators: filtration efficiency for airborne microbial and inert particles. Am Ind Hyg Assoc J..

[78] Code of Federal Regulations (42 CFR 84.180) (2007). Airflow resistance tests.

[79] Banerjee R, Roy P, Das S, Paul MK (2021). A hybrid model integrating warm heat and ultraviolet germicidal irradiation might efficiently disinfect respirators and personal protective equipment. Am J Infect Control..

[80] Burkhart CG (2020). Ozone disinfectants like SoClean CPAP Sanitizer can be used to sterilize cloth and n95 masks in the protection against COVID-19. Open Dermatol J..

[81] Cabaluna ITG, Melicor AF (2020). What are the effective methods of decontaminating N95 mask for reuse?. Acta Medica.

[82] Grossman J, Pierce A, Mody J, Gagne J, Sykora C, Sayood S (2020). Institution of a novel process for N95 respirator disinfection with vaporized hydrogen peroxide in the setting of the COVID-19 pandemic at a large academic medical center. J Am Coll Surg..

[83] Hamzavi IH, Lyons AB, Kohli I, Narla S, Parks-Miller A, Gelfand JM (2020). Ultraviolet germicidal irradiation: possible method for respirator disinfection to facilitate reuse during the COVID-19 pandemic. J Am Acad Dermatol..

[84] Juang PSC, Tsai P (2020). N95 respirator cleaning and reuse methods proposed by the inventor of the N95 mask material. J Emerg Med..

[85] Lawrence C, Harnish DA, Sandoval-Powers M, Mills D, Bergman M, Heimbuch BK (2017). Assessment of half-mask elastomeric respirator and powered air-purifying respirator reprocessing for an influenza pandemic. Am J Infect Control..

[86] Lowe JJ, Paladino KD, Farke JD, et al. N95 filtering facemask respirator ultraviolet germicidal irridation (uvgi) process for decontamination and reuse. Nebraska, USA. 2020. Available at https://www.nebraskamed.com/sites/default/files/documents/covid-19/n-95-decon-process.pdf. Accessed 21 June 2021.

[87] Perkins DJ, Villescas S, Wu TH, Muller T, Bradfute S, Hurwitz I (2020). COVID-19 global pandemic planning: decontamination and reuse processes for N95 respirators. Exp Biol Med (Maywood)..

[88] Sherwood SC, Dixit V, Salomez C (2011). Final report for the Bioquell hydrogen peroxide vapor (HPV) decontamination for reuse of N95 respirators. J Phys Chem A..

[89] Viscusi DJ, Bergman M, Sinkule E, Shaffer RE (2009). Evaluation of the filtration performance of 21 N95 filtering face piece respirators after prolonged storage. Am J Infect Control..

[90] Yim W, Cheng D, Patel S, Kui R, Meng YS, Jokerst JV. Assessment of N95 and K95 respirator decontamination: fiber integrity, filtration efficiency, and dipole charge density. medRxiv [Preprint]. 2020:2020.07.07.20148551. 10.1101/2020.07.07.20148551. Update in: ACS Appl Mater Interfaces. 2020;12(49):54473–54480. 10.1021/acsami.0c17333 PMC7724761 33253527

[91] Zhong H, Zhu Z, You P, Lin J, Cheung CF, Lu VL (2020). Plasmonic and superhydrophobic self-decontaminating N95 respirators. ACS Nano..

[92] Hajifathalian K, Mahadev S, Schwartz RE, Shah S, Sampath K, Schnoll-Sussman F (2020). SARS-COV-2 infection (coronavirus disease 2019) for the gastrointestinal consultant. World J Gastroenterol..

[93] Chughtai AA, Stelzer-braid S, Rawlinson W (2019). Contamination by respiratory viruses on outer surface of medical masks used by hospital healthcare workers. BMC Infect Dis..

[94] Chughtai AA, Seale H, Islam S, Owais M, Macintyre CR. Policies on the use of respiratory protection for hospital health workers to protect from coronavirus disease (COVID-19). Int J Nurs Stud. 2020;105(103567) 10.1016/j.ijnurstu.2020.103567. 10.1016/j.ijnurstu.2020.103567 PMC7174826 32203757

