# Supplementary material for: Mask decontamination methods (model N95) for respiratory protection: a rapid review
Source: Syst Rev. 2021 Aug 7;10:219. doi: 10.1186/s13643-021-01742-1 (PMC8349237; doi:10.1186/s13643-021-01742-1)
Supplement: Supplementary file 1 — Additional file 1. Rapid review protocol. [file 13643_2021_1742_MOESM1_ESM.docx]

**Supplementary Material 1**

**Rapid review protocol**

**Title**: Decontamination methods for respiratory protection mask model N95: a rapid review

**Research question**: How effective and safe are decontamination methods for respiratory protection masks model N95/PFF2 against respiratory viruses?

**PICO**:

P: Healthcare professionals wearing respiratory protection masks model N95

I: Decontamination methods

C: Not applicable

O: Effectiveness, safety, maintenance of protection and filtering characteristics

**Searches**: Medline, Cochrane Library and EMBASE. Keywords: decontamination (eg. “Sterilization”, “Disinfection” and “Decontamination”), reuse (eg. “Equipment Reuse” and “Reuse”), device failure (“Equipment Failure”) And masks (eg. “N95” and “filtering facepiece respirators”)

**Inclusion criteria**: only original research studies in order to evaluate decontamination methods in respiratory masks of the model N95, without limitations of year and language of publication, and study design.

**Exclusion criteria**: studies that evaluated decontamination methods in other types of respiratory masks other than N95. Studies developing possible decontamination methods without carrying out tests themselves were also excluded because they had different objectives.

**Selection of studies**: duplicate, with automatic deletion of duplicates, followed by the reading of titles and abstracts. After the screening, the full text of the selected studies was read to verify the inclusion/exclusion criteria.

**Data extraction**: data related to the author, year, study objective, intervention, comparator, results, limitations, conflict of interest and last year of the search will be extracted.

**Data synthesis**: a narrative synthesis of the results related to the outcomes of interest will be made, grouped by decontamination method.

**Simplified steps**: searches will not be carried out in duplicate; the methodological quality of the included studies will not be assessed and no statistical analysis of the results will be carried out.

**Conflict of interest**: The authors declare that they have no conflict of interest.
