# Supplementary material for: Mask decontamination methods (model N95) for respiratory protection: a rapid review
Source: Syst Rev. 2021 Aug 7;10:219. doi: 10.1186/s13643-021-01742-1 (PMC8349237; doi:10.1186/s13643-021-01742-1)
Supplement: Supplementary file 2 — Additional file 2: Table S1. Search strategies and results from each database on September 25, 2020. [file 13643_2021_1742_MOESM2_ESM.docx]

**Supplementary Material 2**

| **Table 1. Search strategies and results from each database on September 25, 2020.** | | |
| --- | --- | --- |
| **Database** | **Search strategy** | **Results** |
| *Medline* | (("filtering facepiece respirators" OR N95)) AND Equipment Failure | 38 |
|  | (("filtering facepiece respirators" OR N95)) AND Sterilization | 61 |
|  | (("filtering facepiece respirators" OR N95)) AND Decontamination | 98 |
|  | (("filtering facepiece respirators" OR N95)) AND Disinfection | 74 |
|  | (("filtering facepiece respirators" OR N95)) AND Reuse | 110 |
|  | Total | 381 |
| *The Cochrane Library* | #1 - (filtering facepiece respirators):ti,ab,kw" (Word variations have been searched)  #2 - ("N95 respirator"):ti,ab,kw" (Word variations have been searched)  #3 - MeSH descriptor: [Masks] explode all trees  #4 - #1 OR #2 OR #3  #5 - MeSH descriptor: [Sterilization] in all MeSH products  #6 - MeSH descriptor: [Decontamination] explode all trees  #7 - MeSH descriptor: [Disinfection] in all MeSH products  #8 – MeSH descriptor: [Equipment Failure] explode all trees  #9 - MeSH descriptor: [Equipment Reuse] explode all trees  #10 - #5 OR #6 OR #7 OR #8 OR #9  #11 - #4 AND #10 | 52 |
| *EMBASE* | ('filtering facepiece respirator'/exp OR n95) AND ('sterilization'/exp OR 'decontamination'/exp OR 'disinfection'/exp OR 'device failure'/exp OR 'recycling'/exp) | 119 |
| Handsearch | Not applicable | 11 |
