# Supplementary material for: Mask decontamination methods (model N95) for respiratory protection: a rapid review
Source: Syst Rev. 2021 Aug 7;10:219. doi: 10.1186/s13643-021-01742-1 (PMC8349237; doi:10.1186/s13643-021-01742-1)
Supplement: Supplementary file 3 — Additional file 3: Table S2. Studies excluded after full reading and justifications for exclusion. [file 13643_2021_1742_MOESM3_ESM.docx]

**Supplementary Material 3**

**Table 2. Studies excluded after full reading and justifications for exclusion.**

| **1º author, year** | **Study** | **Reason for exclusion** |
| --- | --- | --- |
| Banerjee et al 2020 [79] | A hybrid model integrating warm heat and ultraviolet germicidal irradiation might efficiently disinfect respirators and personal protective equipment. | It does not evaluate a decontamination method. Different outcome. |
| Burkhart 2020 [80] | Ozone disinfectants like soclean CPAP sanitizer can be used to sterilize cloth and N95 masks in the protection against COVID-19 | It is not a primary/experimental study. |
| Cabaluna et al 2020 [81] | What are the effective methods of decontaminating N95 mask for reuse? Asia Pacific Center for Evidence Based Healthcare. | It is not a primary/experimental study. |
| Grossman et al 2020 [82] | Institution of a Novel Process for N95 Respirator Disinfection with Vaporized Hydrogen Peroxide in the Setting of the COVID-19 Pandemic at a Large Academic Medical Center. | It is not a primary/experimental study. |
| Hamzavi et al 2020 [83] | Ultraviolet germicidal irradiation: possible method for respirator disinfection to facilitate reuse during COVID-19 pandemic. | It is not a primary/experimental study. |
| Juang et al 2020 [84] | N95 Respirator Cleaning and Reuse Methods Proposed by the Inventor of the N95 Mask Material. | It is not a primary/experimental study. |
| Lawrence et al 2017 [85] | Assessment of half-mask elastomeric respirator and powered air purifying respirator reprocessing for an influenza pandemic. | The mask model evaluated is not the N95. |
| Lowe et al 2020 [86] | N95 filtering facemask respirator ultraviolet germicidal irradiation (UVGI) process for decontamination and reuse. | It is not a primary/experimental study. |
| Perkins et al 2020 [87] | COVID-19 global pandemic planning: Decontamination and reuse processes for N95 respirators. | The objective is to present a working process to select and implement the use of hydrogen peroxide (HPV) vapor as a viable method for reprocessing N95 respirators in an operating room. |
| Sherwood et al 2011 [88] | Final report for the Bioquell hydrogen peroxide vapor (HPV) decontamination for reuse of N95 respirators. | It is not a primary/experimental study. |
| Viscusi et al 2019 [89] | Evaluation of the filtration performance of 21 N95 filtering facepiece respirators after prolonged storage. | The study does not evaluate any decontamination method, but the storage of the masks. |
| Yim et al 2020 [90] | Assessment of N95 and K95 respirator decontamination: fiber integrity, filtration efficiency, and dipole charge density. | The objective is to compare the N95 and KN95 regarding filtration efficiency, density and fit factor. |
| Zhong et al 2020 [91] | Plasmonic and Superhydrophobic Self-Decontaminating N95 Respirators. | It does not evaluate a decontamination method, but a self-decontaminating respirator. |
