# Supplementary material for: Mask decontamination methods (model N95) for respiratory protection: a rapid review
Source: Syst Rev. 2021 Aug 7;10:219. doi: 10.1186/s13643-021-01742-1 (PMC8349237; doi:10.1186/s13643-021-01742-1)
Supplement: Supplementary file 4 — Additional file 4: Table S3. Decontamination protocols for the 48 studies included. [file 13643_2021_1742_MOESM4_ESM.docx]

**Supplementary Material 4**

**Table 3. Decontamination protocols for the 48 studies included. In order to avoid misinterpretations, especially regarding the methods and results, the information presented in this table was reported as closely as possible to the original articles, which are properly cited in the first column.**

| **Study** | **Protocol** | **Parameters evaluated** | **Commercial mask model** | **Target microorganism** | **Results and conclusions of the study authors** |
| --- | --- | --- | --- | --- | --- |
| **1. Method: Hydrogen Peroxide** | | | | | |
| Viscusi et al 2007 [29] | 1. Liquid hydrogen peroxide: Hydrogen peroxide; Fisher 30% stabilized H_2_O_2_. Based on the treatment vessel's capacity, use either 4 or 8 respirator samples were placed (submerged) into a dishpan or 4L beaker containing 3-5 liters of treatment solution. A second dishpan was used to keep respirators submerged for the time interval the 30 minutes when needed. 1st Test Condition Less aggressive: dunk 3%. 2nd Test Condition More aggressive: dunk 6%. Respirators were removed, hung on a pegboard, and air-dried for 72 hours before filter penetration testing. 2. Vaporized Hydrogen Peroxide (VHP): Two treatment conditions (STERRAD® NX Standard cycle and STERRAD® 100S Standard cycle) were different model instruments using the same process differing by duration and capacity. Respirators tested using these treatments were shipped to and from a commercial facility specializing and were tested inhouse for filtration efficiency within 72 hours of receipt. The STERRAD® sterilization process is less effective when used on cellulose-based products; hence, Tyvek/Mylar pouches were required. As there are no hazardous residues inherent in the STERRAD® process, no aeration interval was necessary. Filter tester and Particle penetration: TSI Model 8130 Automated Filter Tester (AFT) (TSI, Inc., St. Paul, MN) was used to measure filter penetration (Pen). With room temperature with a continuous air+ow of 85 L/minute. Particle penetration was determined using a cubic Plexiglas box (20 x 20 x10 cm3) placed between the filter chucks (sample holding mechanism on the ATF). | - Filter aerosol penetration | Two classes of FFRs were used; the N95 class and the P100 class. A single respirator model from the same manufacturer was selected for both FFR classes tested (3M 8000). | None. | 1. Liquid hydrogen peroxide: Submersion in 3% hydrogen peroxide for 30 minutes exhibited no Average penetration was not significantly increased for either model respirator but was significantly more variable for the P100 respirator. The same treatment with 6% hydrogen peroxide for 30 minutes slightly faded label ink on the respirator's fabric. The average penetration was not significantly changed for the N95 respirator than the baseline and remained well below the NIOSH 5% criteria. The P100 penetration results were significantly more variable compared to baseline but remained less than 0.03%. 2. Vaporized hydrogen peroxide: Aluminum nose bands were slightly tarnished and visibly not as shiny as their as received counterparts after both STERRAD® treatments. The average penetration of both respirator models was not significantly increased for both treatments and remained below NIOSH certification limits. STERRAD® N.X. was found to be slightly less degrading than STERRAD® 100S, but the difference was not statistically significant. FFR did not list cotton as one of the ingredients. Because cellulose absorbs the hydrogen peroxide, care must be exercised not to compromise the decontamination process. Further research will be necessary to determine the effect of the cellulose component on decontamination efficacy. |
| Viscusi et al 2009 [30] | STERRAD® 100S H_2_O_2_ Gas Plasma Sterilizer (Advanced Sterilization Products, Irvine, CA, USA), single 55-min standard cycle. FFRs and a chemical indicator placed in an individual Mylar/Tyvekä self-seal pouch. FFRs were shipped to and from a commercial facility specializing in low-temperature sterilization methods and were tested within 72h of receipt. | - Changes in physical appearance. - Odor changes. - Laboratory performance (filter aerosol penetration and filter airflow resistance). | Random sampling from those N95 FFR models presented in the US Strategic National Stockpile (SNS) and from models commercially available at the time of the study and included because they were considered likely to be more resistant to filtration efficiency degradation and thus offered a more rigorous basis of comparison (3M 8210, 3M 8000, Moldex 2201, KC PFR95-174, 3M 1870 and 3M 1860s) | None. | Metallic nosebands were slightly tarnished and visibly not as shiny when compared with their as-received counterparts. |
| Salter et al 2010 [31] | (1) Hydrogen peroxide 3%: Three FFRs of each model were submerged in liquid decontamination agents in a chemical fume hood for 30 min at room temperature. A volume of 200 mL of decontaminant per FFR was used. After a 30-min soak, the FFRs were removed from the solutions, placed on trays, and allowed to off-gas for 18 hr in a chemical fume hood. Following the off-gassing period, 10 14-mm-diameter samples were punched from areas equally spaced on each respirator and separately weighed in 20-mL glass scintillation vials. The straps, nose cushions, and metal nosepieces were cut into∼12-mm pieces and separately weighed in scintillation vials.  (2) Vaporized hydrogen peroxide (Sterrad 100S System): Triplicate FFRs of each model, packaged individually in sterilization pouches that contained sterilization indicator strips, were exposed to Vaporized hydrogen peroxide for 55 min at 45–55 Cina STERRAD 100S system (Advanced Sterilization Products, Irvine, Calif.) according to supplier directions. Following the sterilization cycle, 14-mm-diameter samples were punched from areas equally spaced on each respirator and weighed in a 20-mL scintillation vial The straps, nose cushions, and metal nosepieces were cut into ∼ 12-mm pieces and separately weighed in vials. IBT analyzed samples, and GC-MS analyzed pentane extracts. | -The amount of residual chemicals created or deposited. | NIOSH, FDA-approved N95, Surgical FFR, NIOSH-approved N95 Particulate FFR | None. | (1) Similar amounts of oxidant remained after treatment with 3% hydrogen peroxide on all FFRs: NIOSH (Average 0.59; 95% CI: 0.14-1.04); FDA-approved N95 (Average 0.36; 95% CI: 0.28-0.45); NIOSH-approved N95 Particulate FFR (Average 0.43, 95% CI: 0.12-0.74); P2 (Average 0.53; 95% CI: 0.20-0.87); P3 (Average 0.70; 95% CI: 0.38-1.02). Except for surgical FFR ND S3, on which no oxidant was detected. (2) The NIOSH, FDA-approved N95, NIOSH-approved N95 Particulate FFR, and P2 models treated with VHP retained∼3 times as much oxidant as the other two models. NIOSH (Average 1.23; 95% CI: 0.68-1.77); FDA-approved N95 (Average 0.43; 95% CI: 0.29-0.57); Surgical FFR (Average 0.36; 95% CI: –0.11-0.83); NIOSH-approved N95 Particulate FFR (Average 1.09; 95% CI: 0.64-1.53); P2 (Average 0.81; 95% CI: 0.29-1.34); P3 (Average 0.35; 95% CI: 0.04-0.66). |
| Bergman et al 2010 [32] | Multiple (3-Cycle) of:  (1) Hydrogen peroxide gas plasma (HPGP): STERRAD® 100S H_2_O_2_ Gas Plasma Sterilizer (Advanced Sterilization Products, Irvine, CA), 59% H_2_O_2_ cycle time ~55-min (short cycle); 45°C–50°C. Samples were packaged in Steris Vis-U-All Low-Temperature Tyvek® /polypropylene–polyethylene Heat Seal Sterilization pouches (six samples per pouch with a chemical indicator strip). Samples were processed at a university medical center. The same pouch was used for all three treatments.  (2) Hydrogen Peroxide Vapor (HPV): Room Bio-Decontamination Service (RBDS™, BIOQUELL UK Ltd, Andover, UK), which utilizes four portable modules: the Clarus® R HPV generator (utilizing 30% H_2_O_2_), the Clarus R20 aeration unit, an instrumentation module, and a control computer. The Clarus® R was placed in a room (64 m3). The instrumentation module measured the HPV concentration, temperature, and relative humidity within the room and monitored by a control computer situated outside the room. Room concentration= 8 g/m3, 15- min dwell, 125-min total cycle time. FFRs were hung on a string stretching across the length of the room. Following HPV exposure, the Clarus R20 aeration unit was run overnight inside the room to convert the HPV into oxygen and water vapor catalytically. The treatments were performed on three consecutive days (one treatment per day). Biological indicators containing Geobacillus stearothermophilus spores were placed in five separate locations inside the room, and a 6-log spore reduction was measured following the 3X treatment.  (3) Liquid hydrogen peroxide* (LHP): 30-min submersion in 6% (one part hydrogen peroxide to four parts of deionized water) solution of hydrogen peroxide. Manufacturing specification: 30% hydrogen peroxide; Cat No. H325-500, CAS Nos. 7722-84-1, 7732-18-5, 12058-66-1 (Fisher Scientific, Fair Lawn, NJ). *Liquid submersion methods. Following each exposure, FFRs were hung on a laboratory pegboard and dried for 16 hours with a fan's aid before repeating the treatment or performing the laboratory aerosol filtration test. | - Filtration performance. - Physical integrity. - Filter airflow resistance. | Six models [three N95 FFR models (N95-A, N95-B, and N95-C) and three surgical N95 respirator models (SN95-D, SN95-E, and SN95-F)] | None. | (1) In this study, the 3-Cycles of Hydrogen peroxide gas plasma (HPGP) treatments resulted in mean penetration levels > 5% for four of the six FFR models. Of the 36 samples that underwent HPGP processing, nine samples had % penetration levels > 5%.  (2) 3X Hydrogen Peroxide Vapor (HPV) did not cause any observable physical changes to the FFRs.  (3) For those models with staples (N95-B, N95-C, SN95-E, and SN95-F), liquid hydrogen peroxide treatment caused staples to oxidize to varying degrees; this effect was not observed following the 3X HPGP and HPV treatments. |
| Cheng et al 2020 [33] | SteraMist™ Binary Ionization Technology® (BIT™) solution delivered through the SteraMist™ Surface Unit, registered with the U.S. Environmental Protection Agency. The main constituent contains a 7.8% H_2_O_2_ solution, which converts to ionized H_2_O_2_ (iHP) after passing through a cold plasma arc and moving like a gas throughout the surface of the N95 respirator. The by-product of iHP is oxygen and water in the form of humidity. The experiment was conducted in a well-ventilated room with 6 air-change-per-hour inside the biosafety level-2 microbiology laboratory with the operator wearing a coverall protection gown. | - Disinfection capacity | 3M1870 and 3M1860s | Influenza A virus subtype H1N1. | The virus was eluted from the N95 respirators for viral culture in Madin-Darby Canine Kidney (MDCK). Cytopathic changes in MDCK cells were observed daily for 7 days by microscopy. All iHP-treated pieces of N95 respirators did not demonstrate cytopathic changes suggestive of the influenza A virus. They were sub-cultured to the MDCK cell line again for another 7 days for cytopathic observation. No sign of cytopathic changes were observed after 7 days, and it was finally confirmed by a negative influenza An antigen detection by an immunofluorescence staining procedure. This experiment showed that the spray of iHP could kill the influenza A virus at moderate to a high level of inoculum. Healthcare workers should be reminded not to reuse the N95 respiratory immediately after disinfection. In our experiment, the level of H_2_O_2_ on the inner surface of the N95 respirator was 0.6ppm (lower than the safety limit of <1ppm) at 2 hours and undetectable at 3 hours. |
| Fischer et al 2020 [34] | Vaporized hydrogen peroxide (VHP) - Plates with fabric and steel discs were placed into a Panasonic MCO-19AIC-PT (PHC Corp. of North America Wood Dale, IL) incubator with VHP generation capabilities and exposed to hydrogen peroxide (approximately 1000 ppm). The exposure to VHP was 10 minutes. After the inactivation of the hydrogen peroxide, the plate was removed, and 1 mL of cell culture medium was added. | - Disinfection capacity  - Mask Integrity was quantitatively determined using the Portacount Respirator fit tester | N95 respirators (3M Aura Particulate Respirator 9211+/37193) AOSafety N9504C respirators (Aearo Company Southbridge, MA). | HCoV-19 nCoV-WA1-2020 (MN985325.1) | Vaporized hydrogen peroxide yielded extremely rapid inactivation both on N95 and on stainless steel. Filtration performance of the N95 respirator was not markedly reduced after single decontamination. The VHP-treated masks retained comparable filtration performance to the control group after two rounds of decontamination and acceptable performance after three rounds. |
| Schwartz et al 2020 [35] | The Hydrogen Peroxide Vapor run consisted of the following five stages: Conditioning, Pre‐gassing, Gassing, Gassing Dwell, and Aeration. The existing RBL Hydrogen Peroxide Vapor standard operating procedure (SOP) was employed and required that the processing room attain 480+ ppm level of Hydrogen Peroxide Vapor with a “Gassing” time of 25 minutes and a “Gassing Dwell” time of 20 minutes. At the end of a cycle, fresh air is introduced into the room to increase the rate of catalytic conversion of hydrogen peroxide vapor into oxygen and water during the aeration stage. This procedure leaves no residue other than water. When sufficient time had passed, they used a PortaSens II™ sensor to ensure hydrogen peroxide levels were below the OSHA Permissible Exposure Limit3 (PEL) of 1.0ppm before entering the room. | - Maintenance of functions as designed, using a standardized human N95 fit testing methodology.  - Disinfection capacity | 3MTM, 1860 N95 | *Geobacillus stearothermophilus spores* | For the quantitative assessment, the PortaSens II was used to detect H_2_O_2_ levels over a 4-hour time frame by taking readings at regular intervals by placing the probe close to the respirators. At approximately 4 hours, the levels of H_2_O_2_ decreased below the PortaSens II level of detection (0 ppm). In the qualitative test, 3 individuals did a qualitative smell test to determine any noticeable odors. None were detected. After complete aeration, the respirators went through a QA process to ensure no physical or performance degradation. The decontaminated respirators then underwent our normal quantitative fit testing process to ensure their continued performance. They were tested on 2 individuals with different facial structures with no loss of fit or seal, thereby ensuring that N95s decontaminated using this method can be placed back into circulation. They validated the efficacy of the decontamination process by using 9 individual 6-log biological indicators (*Geobacillus stearothermophilus spores*). |
| Cai and Floyd 2020 [36] | Experiments were conducted using a test chamber. A stable salt aerosol was generated using a 3-jet Collison nebulizer (CH Technologies) and 2% sodium chloride solution by the National Institute of Occupational Safety and Health procedure No. TEB-APR-STP-0059. A scanning mobility particle sizer (model 3936; TSI) was used to measure the particle number concentration from 16.8 nm to 514 nm. All masks were preconditioned in an incubator at 38 °C and 100% relative humidity for 12 hours. For each mask, 5 samples were tested with 4 upstream measurements and 4 downstream measurements. Acceptable pressure drop was defined as less than 35 mm or 1.38-inch water for inhalation. | - Filtration performance | N95s (model 1860; 3M) N95s (Civilian Antivirus; Qingdao Sophti Health Technology) | None. | The mean (SD) filtration efficiencies of untreated masks were 97.3% (0.4%) for N95s and 96.7% (1.0%) for KN95s. After H_2_O_2_ sterilization, the filtration efficiencies were 96.6% (1.0%) for N95s and 97.1% (2.4%) for KN95s. |
| Lieu et al 2020 [37] | The participants received teaching on the proper quality check, seal, donning, and doffing of the respirator. Each participant was fit-tested with their current N95 respirator or with the ProGear and solution the denatonium benzoate (Bitrex FT-32, 3M) or sweet saccharin solution (FT-12, 3M). Masks were identified for proper storage and returned to the same user after decontamination. Participants were expected to wear the respirator for a total of 4 hours, with temporary removal was permitted, for example, to drink. Decontamination was performed with VHP (V-PRO maX Low-Temperature Sterilization System; Steris, Mentor, OH), set to a non-lumen setting, and lasted 28 minutes. Each respirator was separately placed in a Tyvek 8 × 14 inch pouch. A verified vaporized H_2_O_2_ process indicator adhesive label was added to each pouch. Before reusing the decontaminated respirator, he was visually inspected for loss of integrity. Participants used to assess respirator's structural and functional integrity. | - Respirator failure, defined as either fit-test failure or mechanical failure. - Overall number of cycles required for half of the respirators to fail. | Models 1860, 1860s, and 1870+ from 3M, models 1510, 1511, 1512, and 1517 from Moldex, and model 88020 from ProGear. | None. | The overall median number of cycles completed before mechanical or fit test failure was 2. Only fittest failures, the median number of decontamination cycles that could be performed before fit test failure was 4. However, there was a marked variation between respirator models in the number of cycles that could be performed before failure. The overall number of cycles required for half of the respirators to fail for the models 1860(S) was 1, 1870+ was 3, Moldex 151X was 5, and ProGear was 4. The overall number of cycles required for half of the respirators to fail fit testing was 1 for the 1860(S), 4 for the 1870+, 5 for the Moldex 151X, and 4 for the ProGear. Finally, the proportion of masks that failed fit testing after a single cycle of extended use and decontamination was 66% for 1860, 22% for the 1870+ and the Moldex, and 0% for the ProGear. Overall, the user seal check was concordant with the qualitative fit test in 81.3% (n = 75) of cases. Participants were able to detect leakage on seal check-in on 4 occasions, all of which corresponded to failure with the qualitative fit testing. However, the user seal check failed to predict fit test failure in 14 of 18 cases (77.8%) of fit test failures. Decontamination and prolonged usage of respirators must be done cautiously. |
| Levine et al 2020 [38] | Respirators were decontaminated using VHP via 100 the Steris VHP system (Steris Life Sciences, Mentor, OH). Fit testing was administered following OSHA standard with minor modifications: 1) N95 respirators tested using both qualitative fit tests (QLFTs) and quantitative fit test (QTFTs) underwent QLFTs first, 2) QLFT users were blinded to the order by which qualitative testing was administered. To evaluate VHP decontamination's effect on N95 respirator integrity, new, unworn N95 respirators were decontaminated consecutively for up to eight cycles. Following each decontamination cycle, a subset was removed for quantitative fit testing. | - Fit Testing. - The integrity of decontaminated N95 respirators in the face shape of a second user | Halyard Fluidshield N95 46727 3M 1860 N95 3M 1860S N95 3M 1870 N95 3M 9210 N95 | None. | Both 3M 1860/3M 1860S and 3M 1870 N95 respirators maintained integrity following up to eight and six cycles of VHP decontamination. There was also no significant difference in the integrity of 3M 9210 following up to seven decontamination cycles. The clear downward trend in the integrity of Halyard Fluidshield 46727 N95 respirators throughout eight decontamination cycles. There was a significant decrease in the integrity of 3M 1870 N95 respirators when the respirator was lightly worn, decontaminated for six cycles with VHP, and fit-tested by a second user compared to a respirator of the same model and size that had undergone the same number of decontamination cycles but was never previously used. No significant differences were observed in the integrity of 3M 1860/3M 1860S, 3M 9210, and Halyard Fluidshield 46727 N95 respirators when fit-tested by a second user following multiple rounds of decontamination. Data revealed variability in the integrity of different N95 models after VHP decontamination and potential limitations of N95. |
| Ludwig-Begall et al 2020 [39] | Porcine respiratory coronavirus (PRCV strain 91V44) was passaged three times on confluent cell monolayers of the ST continuous cell line. A virus stock with a titer of 107.8 TCID50/mL was used. One negative control mask or respirator (not contaminated but treated), three treated masks or respirators (PRCV-contaminated and treated), and three positive controls (PRCV-contaminated but untreated), i.e., seven masks in total, were utilized. FFRs and a chemical indicator were placed in individual Mylar/Tyvek pouches. Vaporous hydrogen peroxide (VHP) treatment was performed with the V-PRO Max Sterilizer (Steris, Mentor, OH), which uses 59% liquid H_2_O_2_ to generate hydrogen peroxide vapor. A 28-min non-lumen cycle was used, consisting of 2 min 40 s conditioning (5 g/min), 19 min 47 s decontamination (2.2 g/min), and 7 min 46 s aeration. Peak VHP concentration was 750 ppm. | - Disinfection capacity | KN95 FFR – Guangzhou Sunjoy Auto Supplies CO. LTD, Guangdong, China (2020 N°26202002240270) | *Porcine respiratory coronavirus* (PRCV) | Recovery from FFR coupons yielded a mean infectious virus at 1.80 × 105 (± 1.58) TCID50/mL for positive controls of the H_2_O_2_ assays. Recovery values for the infectious virus from FFR straps used as a positive control in the H_2_O_2_ experiments were 1.75 × 103 (± 0.43) TCID50/mL. FFR coupons decontaminated via H_2_O_2_ reduced viral titers by over five orders of magnitude (1.80 × 105 (± 1.58) TCID50/mL). Hydrogen peroxide treatment of FFR straps reduced the infectious virus load by 1.69 × 103 (± 0.43) TCID50/mL. |
| Jatta et al 2020 [40] | N95 respirators were treated with 5 and 10 cycles of VHP by the V-PRO maX Low-Temperature Sterilization System. A biological indicator (BI) containing Geobacillus stearothermophilus placed into the center of each load. The masks were processed on a 28-minute non-lumen cycle per instructions provided with the V-PRO maX Low-Temperature Sterilization System. After each cycle, packaged masks are cooled to room temperature before being subjected to a new sterilization cycle. A fit factor of at least 100 for half-mask respirators was used as the minimum fit factor pass level. Subjects that had confirmed fit for the specified masks were tested with an unprocessed mask (control) and masks that had been reprocessed but not used in the clinical They evaluated 2 masks of each model that were treated with 5 or 10 decontamination cycles. | - Filtration efficiency - Quantitative fit testing per Occupational Safety and Health Administration standards | 3M 8211 FF and 9210 FF | None. | The overall fit factor for either the 3M 8211 and 9210 respirators using quantitative fit testing was consistently above the minimum fit factor pass level of 100 needed for half-mask respirators before and following VHP sterilization cycles. Individuals who were fit tested did not report odor or facial irritation. Straps remained functional for both models and at all cycles. The masks reprocessed for 15 cycles were reported to be tighter and uncomfortable on the face. VHP sterilization did not demonstrate an effect on filtration efficiency. A minor drop was observed for particles smaller than 70 nm with the 3M 8211 sterilized with 10 cycles. However, the difference was not significant (p > .05) between this respirator and an untreated one (0 cycles). The 3M 8211 respirator treated for 5 cycles demonstrated significantly higher filtration efficiency than the other 2 for particles smaller than 100 nm (P < .05), though the difference was still small. The 3M 9210 model treated with 5 cycles or 10 cycles demonstrated no significant difference compared to the untreated one (P > .05). |
| Widmer and Richner. 2020 [41] | Used FFP2 respirators were collected in designated containers and stored for 24 h at Central Sterilization. The staff, protected with appropriate PPE, checked under the magnifying glass each FFP2 respirator for intact surfaces, debris, and visual changes, such as residual lipstick, make-up, and other residuals from humans. Respirators that passed this test were individually packed in bags as recommended by the manufacturer. The respirators were sterilized by vaporized hydrogen peroxide (H_2_O_2_) low temp sterilization using the V-PRO® maX Low-Temperature Sterilization System (STERIS 5960 Heisley Road Mentor, OH 44060, USA). Then the respirators were tested according to EN 149 after reprocessing. DIN EN 149 is the European standard for FFP respirators, similar to the American standard N95 (NIOSH-42CFR84). The assessment is based on testing the total inward leakage of masks on 10 test individuals. New and reprocessed respirators (each n = 10) have been tested at Spiez Laboratory, Federal Office for Civil Protection FOCP, Switzerland. The total inward leakage of the protective respirators was quantitatively tested in an atmosphere charged with paraffin aerosol, according to EN 149. The aerosol concentration was measured with the Portacount® Pro+ Respirator Fit Tester 8038 (TSI Incorporated, Shoreview, USA) outside and inside the respirator, while the test persons were performing a series of tasks (movement) according to EN 149. The total inward leakage is the ratio of outside and inside paraffin aerosol concentrations. The fit factor (FF) is the inverse of the total inward leakage. | - Filtration efficiency outlined by EN 149. | FFP2 respirators – Model Aura™ 1862+ | No specific microorganism. | The assurance level requires a 6 log reduction of the spores most resistant to the sterilization technique. SARS-CoV-2 belongs to the enveloped viruses that are highly susceptible to this sterilization technique and are therefore killed by this process. Individually packed FFP2 respirators demonstrated 1.5 ± 0.1 mg/m3 hydrogen peroxide immediately after sterilization. Unpacked respirators showed a relatively high concentration of H_2_O_2_ immediately after sterilization (3.6 ± 1.5 mg/ m3 H_2_O_2_). However, aeration for 24 h leads to shallow values of 0.2 ± 0.1 mg/m3 H_2_O_2_, which is much below the maximum allowed concentration of 0.7 mg/ m3 H_2_O_2_. After one single reprocessing cycle, all tested FFP2 respirators fulfilled the requirement of the standard EN 149 for FFP2 (Fit Factor > 13, corresponding to a total inward leakage of 8%) |
| Peltier et al 2020 [42] | gpHP (gas-plasma hydrogen peroxide: Respirators were donned on a mannequin covered in a layer of soft closed-cell foam. The mannequin was installed in a 0.1-m3 exposure chamber and flooded with polydispersed combustion aerosol. Protocol 1- Sterrad 100S. No. of Respirators Tested (1); Cycle or Dwell Time (min) 55; Repeated Decontamination Treatments (1x).Protocol 2 -Sterrad 100NX Express No. of Respirators Tested (5); Cycle or Dwell Time (min) 28; Repeated Decontamination Treatments ( 1x, 3x, 5x) Protocol 3 - Sterrad 100NX Standard No. of Respirators Tested (1); Cycle or Dwell Time (min) 47; Repeated Decontamination Treatments ( 1x) VHP (vaporized hydrogen peroxide) Respirators were donned on a mannequin covered in a layer of soft closed-cell foam. The mannequin was installed in a 0.1-m3 exposure chamber and flooded with polydispersed combustion aerosolProtocol 1- Battelle CCDS. No. of Respirators Tested (1); Cycle or Dwell Time (min) 150; Repeated Decontamination Treatments (1x).Protocol 2 - Bioquell No. of Respirators Tested (1); Cycle or Dwell Time (min) varies; Repeated Decontamination Treatments ( 1x); Protocol 3- Steris V-Pro Max II No. of Respirators Tested (10); Cycle or Dwell Time (min) 28; Repeated Decontamination Treatments ( 1x through 10x) | - Filtration efficiency - Respiratory performance, | 3M 1860 or 1860S models (3M, StPaul, MN). | None. | Respirators that were treated with vHP, or shorter decontamination cycles of gpHP, retained their original filtration capabilities. These included Battelle Critical Care Decontamination System (CCDS) and Bioquell (both processed just once), or Sterrad 100NX Express (processed up to 5 times). Steris V-Max Pro produced similar results; respirators maintained their filtration efficiency up to 10 treatments. In contrast, gpHP treatment (by Sterrad 100S or Sterrad 100NX standard) degrades respirator filtration performance, with substantially decreased collection efficiencies across the entire size distribution. |
| Cramer et al 2020 [43] | One to five sterilization cycles using ionized hydrogen peroxide (iHP), generated by SteraMist® equipment (TOMI; Frederick, MD). Main outcome measures: Personal protective equipment, including five N95 mask models from three manufacturers, were evaluated for efficacy of sterilization following iHP treatment. | sterility, filtration efficiency,fit | 3M1860; KC/Halyard 46767 (duckbill); Gerson 2130; 3M 8210; 3M 9210/37021 | *Geobacillus stearothermophilus spores* | The was retain function up to five cycles. Some but not all PPE could also be sterilized using an iHP environmental chamber. However, pre-treatment with a handheld iHP generator was required for semi-enclosed surfaces such as respirator hoses. A typical iHP environment chamber with a volume of ~80 m3 can treat ~7000 masks per day. |
| Ibáñez-Cervantes et al 2020 [44] | N95 masks artificially contaminated with SARS-CoV-2, Staphylococcus aureus, and Acinetobacter baumannii bacteria were individually placed in 20 cm X 20 cm sterilization bags “Tyvek” (with STERRAD chemical indicator and STERRAD VELOCITY biological indicator). They were hermetically sealed and subjected to disinfection by using a STERRAD 100NX sterilization system (ASP), by using the “standard STERRAD NX” sterilization cycle under 47 minutes. | - Desinfection capacity | 3M 8210 | *- SARS-CoV-2, Staphylococcus aureus and Acinetobacter baumannii* | Hydrogen peroxide plasma inhibited the detection of the SARS-CoV-2 in all virus dilutions by RT-PCR (compared to control). It killed Acinetobacter baumannii and Staphylococcus aureus (not cultivable with inoculums of 102 to 106 CFU). |
| Saini et al 2020 [45] | To compare the efficacy of vaporized hydrogen peroxide (VHP) sterilization method with the heat and alcohol-based to disinfect PPE (coveralls, face-shields, and N-95 masks), were used a range of concentrations of hydrogen peroxide by diluting the hydrogen peroxide stock to 6, 8 and 10% with distilled water - VHP generator (SATEJ Plus machine), VHP cycle (7–8% Hydrogen peroxide) housing room of about 1200 cubic feet (length10 ft × breadth 10 ft × height 12 ft). The PPE integrity performed droplet permeations tests and microscopic analysis using Scanning Electron Microscopy (SEM). Disinfection with three biological indicators as control. | -Decontamination efficiency, physical features, permeability, fabric integrity | Not mentioned. | *Escherichia coli, Mycobacterium smegmatis* and spores of *Bacillus stearothermophilus* | VHP treatment completely cleared two biological indicators (E. coli and M. smegmatis), and a 100% deactivation of B. stearothermophilus spores spiked at high exposure area on N-95 masks. Physical tear or deformity, macroscopic, and microscopic alterations were found in in-house permeation tests and SEM studies. The results are consistent and reproducible as tested in over 10 cycles. |
| Russo et al 2020 [46] | Used N95 respirators were decontaminated with vaporized hydrogen peroxide (VHP) using a VHP® VICTORY TM unit (Steris Life Sciences, Mentor, OH) filled with a 35% aqueous hydrogen peroxide solution (Vaprox@). The VICTORYTM unit was remotely controlled by a computer using SmartPhaseTM software technology that automatically adjusted VHP injection rates based on room temperature, relative humidity, and VHP concentration.N95 respirators were hung on metal racks, stacked in piles, placed in paper bags or covered with makeup or moisturizer. | -Decontamination efficiency Novel approaches to improve workflow | 3M 9210, 3M 1870, 3M 1870+, 3M 1860S, and 3M 1860 (3M, St. Paul, MN; Cardinal Health, Dublin, OH, Gerson, Middleboro, MA); Cardinal Health S and M/L (Cardinal Health, Dublin, OH), Gerson 2130 and Gerson 1730; (Gerson, Middleboro, MA), Halyard Fluidshield 46727 and 46827; (O&M Halyard, Inc., Alpharetta, GA) | No specific agent. | It took 90 minutes for VHP concentrations to reach a level reported to reduce microorganisms by 12-logs. All N95 respirator models were decontaminated when stacked in piles of eight or fewer. Using a six-stack piling strategy would increase the capacity of a facility from 4,250 to 12,240 units. N95 respirators can be decontaminated in labeled paper bags. It would speed up and facilitate the sorting after the decontamination process. Makeup and moisturizer creams did not interfere with the decontamination process. |
| Keney et al 2020 [47] | Evaluated HP vapor's virucidal activity using a BQ-50 system (Bioquell) after inoculating 3M 1870 N95 respirators (3M, St. Paul, MN) with 3 aerosolized bacteriophages that are a reasonable proxy for SARS-CoV-2. Inoculation resulted in contamination of the respirator with 9.87e4 plaque-forming units (PFU) of phage phi-6, 4.17e7 PFU of phage T7, and 1.35e7 PFU of phage T1. Respirators were reprocessed with BQ-50 with a long aeration phase to reduce vapors. A standard plaquing assay measured the virucidal activity before and after sterilization. | - Virucidal activity | 3M 1870 N95 | 3 aerosolized bacteriophages: T1, T7, and Pseudomonas phage phi-6. | A single HP vapor cycle resulted in complete eradication of phage from masks (limit of detection 10 PFU, lower than the infectious dose of most respiratory viral pathogens). |
| **2. Method: Ultraviolet germicidal irradiation** | | | | | |
| Viscusi et al 2007 [29] | Respirators were placed on a laminar flow hood's working surface, Sterilgard III (The Baker Company, Sanford) fitted with a 40W ultraviolet light for general decontamination. The intensity is reported as the average obtained at nine positions over the area used with a UVX Digital Radiometer with MODEL UVX-25 sensor (254nm filter) (VWR Lab Shop). For both treatments, samples were turned over after 50% exposure to allow treatment of the inside and the outside of the respirator. 1st Test Condition Less aggressive: 30 minutes. 2nd Test Condition More aggressive: 480 minutes. TSI Model 8130 Automated Filter Tester (TSI, Inc., St. Paul, MN) was used to measure filter penetration. With room temperature with a continuous airflow of 85 L/minute. Particle penetration was determined using a cubic Plexiglas box placed between the filter chucks. | - Filtration performance | Two classes of FFRs were used; the N95 class and the P100 class. A single respirator model from the same manufacturer was selected for both FFR classes tested. | None. | No significant visible changes were observed for any samples after. The average penetration results for N95 respirators were not significantly affected by either treatment. The P100 respirators’ penetration was unchanged by the 30-min. Treatment. For the 480-min. P100 penetration results were significantly more variable than the baseline but remained less than 0.03%. The data reported indicated that important to control UV exposure duration and that irradiation for 2 hours was appropriate. |
| Vo et al 2009 [48] | Using a UV germicidal lamp in a biological safety cabinet (SterilGARD III model SG403A; Baker Company, Sanford, ME). A low-pressure mercury arc lamp (5.5 mg Hg; lamp type, TUV 36TS 4P SE; lamp voltage, 94 V; lamp wattage, 40 W; wavelength, 253.7 nm) was used the UV source. The UV intensity on the sample surface was measured using a UVX-25 digital radiometer (model E28457; Cole-Parmer, Vernon Hills, IL). After exposure to MS2, and FFR was treated with UV irradiation using a UV source intensity of 0.4 mW/cm^2^ at the FFR surface (distance from lamp to sample surface, 42 cm). Because of the relatively low intensity, long irradiation times (1, 2, 3, 4, and 5 h; control, 0 h) were used. The survival of the MS2 virus on the FFRs at different times was also examined by storing the MS2-contaminated FFR samples for 1, 2, 3, 4, and 5 h (at the same temperature and RH) but without UV irradiation (control experiments). Three replicate tests were carried out for each irradiation time and each control experiment | -Effectiveness -Decontamination decreases in the number of viable viruses recovered from the respirators. | N95 FFR (modelo N1105; Willson, Santa Ana, CA) | MS2 coliphage (ATCC 15597-B1) | For UV decontamination at a wave length of 254 nm, an approximately 3-log reduction in the level of MS2 virus was achieved with a dose of 4.32 J/cm2 doses of UV irradiation (>7.20 J/cm^2^; 3 h of contact time with a UV intensity of 0.4 mW/cm^2^; UV intensity, 0.4 mW/cm^2^), while with higher; contact times, >5 h, all MS2 was inactivated. |
| Viscusi et al 2009 [30] | FFRs were placed on a Sterilgard III laminar flow cabinet (The Baker Company, Sanford, ME, USA) fitted with a 40-W UV-C light (average UV intensity experimentally measured to range from 0.18 to 0.20 mW cm^−2^). Fifteen-minute exposure to each side (outer and inner), 176–181 mJ cm^−2^ exposure to each side of FFR | - Changes in physical appearance - Odor changes - Laboratory performance (filter aerosol penetration and filter airflow resistance) | Random sampling from those N95 FFR models present in the US Strategic National Stockpile (SNS) and from models commercially available at the time of the study and included because they were considered likely to be more resistant to filtration efficiency degradation and thus offer a more rigorous basis of comparison. | None. | No visible changes were observed for all samples. |
| Salter et al 2010 [31] | Ultraviolet light (254 and 302 nm,∼2.7X105J/m^2^. Triplicate 38-mm-diameter circles were cut from each FFR model. A multi-wave length, 8-watt lamp (Ultra-Violet Products) was used to expose each FFR model's triplicate samples to UV light. Samples were placed 1 inch from the lamp source and were irradiated with 4.0 mW/cm^2^ of UV-B (302 nm) and 3.4 mW/cm^2^ UV-C (254 nm) for 1 hr each. A UV meter (Ultra-Violet Products) was used to measure irradiance. After exposure, samples were weighed in 20-mL glass scintillation vials and extracted with pentane as described for liquid decontaminants. | -The amount of residual chemicals created or deposited | NIOSH, FDA-approved N95, Surgical FFR, NIOSH-approved N95 and Particulate FFR | None. | UV irradiation produced the greatest number of unique peaks; however, many appear to be constituents of the pentane solvent |
| Bergman et al 2010 [32] | Multiple (3-Cycle) of UV Bench Lamp (UV-C, 254 nm, 40 W), Model XX-40S (UVP, LLC, Upland, CA). 45-min exposure at intensity 1.8 mW/cm^2^ (note: one 45-min continuous exposure constitutes the 3X cycle). Test tube racks were placed beneath both ends of the lamp to lift the lamp ~ 25 cm from a laboratory hood's working surface. The UV intensity was reported as the mean of 27 measurements over the rectangular area used at the hood's surface using a UVX Digital Radiometer with a model UVX-25 Sensor (254 nm filter) (UVP, LLC, Upland, CA). Only the exteriors of the FFRs were exposed. The duckbill and flat fold style FFRs were placed over beakers to facilitate exposure to the FFR surface. | - Filtration performance - Physical Integrity - Filter airflow resistance | Six models:  -Three N95 FFR models (N95-A, N95-B, and N95-C)  -Three surgical N95 respirator models (SN95-D, SN95-E, and SN95-F)] | None | The 3X UVGI treatment had similar mean % penetration to 1X treated samples tested previously for the same six models. No visible changes were observed for all samples. |
| Fisher et al 2011 [49] | The αIFM was used to categorize FFRs as most shielded (≤5%), moderately shielded (>5% but ≤30%), and least shielded (>30%). UV-C decontamination of MS2 was determined for FFR coupons excised from two FFR models from each shielded category. For models C and F, the MS2-contaminated coupons were exposed to UV-C (25 ± 1.0Wm^-2^) for combined bidirectional treatment times of 1, 2, 4, and 10 min (0.5, 1, 2, 5 min per side). Only the 10-min exposure was performed on model A because of a low αIFM. A supplemental experiment for Model A was performed using a bidirectional treatment time of 5 h. Specific UV-C doses (J m^-2^) to the IFM (DIFM) doses ranging from 38 to 4707 J m^-2^. | - Filtration efficiency - Airflow resistance - Reductions MS2 bacteriophage. | Cardinal N95‐ML (Model A); Wilson SAF‐T‐FIT® Plus (Model B); 3M™ 8210 (Model C); 3M™ 1860 (Model D); 3M™ 1870 (Model F); and the Kimberly‐Clark PFR95‐174 (Model E). | MS2 bacteriophage. | The supplied dose at each treatment time was the same for each FFR model, but the DIFM varied because of the model-specific αIFM values. Log reductions (LR) within a given treatment time differed among the FFR models. A 10-min treatment produced LRs of 0.1 (model A), 2.9 (model C), and >4.8 (model F). Across models A, C, and F and within models C and F, LR increased with increasing DIFMs. The calculated treatment times and log reductions (LR) for models B, D, and E: The treatment times required to reach the targeted DIFMs of 300, 1000, and 3000 J m^-2^ differed for each model. LRs were statistically different among the models for DIFMs of 300 J m^-2^ (P=0.006) and 3000 J m^-2^ (P<0.001). The results for the 1000 J m^-2^ treatment among the models were statistically similar (P=0.79). LRs increased with increased DIFM for models B (P<0.001) and D (P<0.001). The LRs reported for model E did not differ significantly among all three doses (P=0.18). Exposure times were required to achieve a DIFM of 1000 J m^-2^ for each model-specific αIFM with a UV-C irradiance of 25 W m^-2^, ranging from 266 to 2 min. LRs for the approximate 1000 J m^-2^ DIFM ranged from 2.86 to 3.59, with the lowest LR value (2.86) corresponding to the lowest DIFM (917 J m^-2^). |
| Heimbuch et al 2011 [50] | A 120-cm, 80-W UV-C (254 nm) lamp (Ultraviolet Products, Upland, CA) was adjusted to a height of 25 cm. The output from the lamp was measured using a radiometer (Ultraviolet Products). The range of UV irradiation to which the FFR was exposed varied from 1.6 mW/cm^2^ to 2.2 mW/cm^2^.The exterior surface of H1N1-contaminated FFRs was irradiated for 15 minutes, which provided an average dose of 18 kJ/m^2^. | - Inactivation of the H1N1 virus | 6 FFR models (3 particulate, designated P1-P3, and 3 surgical, designated S1-S3). | H1N1 influenza virus. | Provided an average >4-log reduction of viable H1N1 influenza virus against both the droplet and aerosol challenges for all 6 FFRs. No other FFRs showed noticeable deterioration or deformation. |
| Viscusi et al 2011 [51] | FFRs placed on a laboratory stand inside a Sterilgard III laminar flow cabinet (The Baker Company) fitted with a 40W UV-C bulb, Intensity 1.8 mW/cm^2^ measured with a UVX Digital Radiometer with MODEL UVX-25 sensor (254 nm filter) (VWR Lab Shop, Batavia, Ill.). Total exposure was 30 min (15 min each FFR side) | - Tolerability. - Usability (specifically, odor, comfort, or donning ease). | (1) 3M 8000, (2) 3M 8210, (3) Moldex 2200, (4) 3M 1860, (5) 3M 1870, and (6) Kimberly Clark PFR95–270. | None. | Test technicians did not report any unusual or strong odor from FFRs following the decontamination method. |
| Bergman et al 2011 [52] | The FFR with 15 min exposure to the outer side, were placed on a laboratory stand inside a Sterilgard III laminar flow cabinet (The Baker Company, Sanford, ME) fitted with a 40 W UV-C bulb. Intensity 1.8 mW/cm2 was measured with a UVX Digital Radiometer with a model UVX-25 sensor (254 nm filter) (VWR Lab Shop). | - Respirator fit (odor, comfort, or donning ease) | Three models of surgical N95 respirators (3M 1860, 3M 1870, and Kimberly Clark PFR95-270) | None | UVGI did not cause degradation in any FFR models. |
| Lore et al 2012 [53] | A 126- (L) X 15.2- (W) X 10.8-cm (H), dual-bulb, 15-W UV-C(254-nm wavelength) lamp (Ultraviolet Products, Upland, CA, USA) was placed in a Labgard class II, type A2, laminar flow cabinet (NuAire, Inc., Ply-mouth, MN, USA) set to a height 25 cm above the cabinet’s working surface. Measured by a UVX digital radiometer (UVP Inc., Upland, CA, USA), the lamp’s UV-C wavelength irradiance ranged between1.6 mW cm^-2^ and 2.2 mW cm^-2^ | - Virucidal capability. - Post-decontamination filter performance | 3M models 1860s and 1870 | Influenza A H5N1 virus. | Showed that the decontamination method whit UVGI satisfactorily decontaminated the 3M1860s and 1870 FFRs measured by a virus culture method. Within the experiment's constraints, the three methods were all completely effective for the decontamination of FFRs as assessed by a culture method. |
| Woo et al 2012 [54] | Filters were contaminated by different transmission pathways (droplet and aerosol) using three spraying media (deionized water [DI], beef extract [BE], and artiﬁcial saliva [AS]) under different humidity levels (30% [low relative humidity {LRH}], 60% [MRH], and 90% [HRH]). UV exposure whiff the UV-C lamp (UVG-11; 254nm, 230 V, 4 W; UV Products, Cambridge, United Kingdom) was adjusted to a height of 10 cm. UV intensity of 1.0 mW/cm^2^ has been measured with a radiometer (PS-300; Apogee, Logan, UT). The quadrants were placed on a petri dish in a chamber and exposed to UV for different exposure times (0 to 2 h). One quadrant used as a control was not exposed to UV. All were evaluated after the maximum exposure time. After the maximum exposure time, each quadrant was placed in a 50-ml conical tube containing sterilized DI water and agitated to extract MS2, assayed with the single-layer method. IE was determined by comparing the count from the irradiated coupon to that from the paired control. | - Decontamination efﬁciency by UV | FFR 3M 1870 (NIOSH-certiﬁed N95) | MS2 bacteriophage (MS2; ATCC  15597-B1) | UV irradiation at constant intensity was applied for two-time intervals at each relative humidity condition. The highest inactivation efﬁciency, around 5.8 logs, was seen for deionized water aerosols containing MS2 on ﬁlters at LRH after applying a UV intensity of1.0 mW/cm^2^ for 30 min. The IE of droplets containing MS2 was lower than that of aerosols containing MS2. |
| Lindsley et al 2015 [55] | Respirator coupons and straps were exposed to ultraviolet light with a primary wavelength of 254 nm (UV-C) in a custom-made 91 cm × 31 cm × 64 cm high chamber. The chamber was fitted with two 15 Watt T-150 254 nm UV-C lamps in a reflective housing and lined with black felt to minimize reflections. UV-C irradiance was measured using a radiometer (ILT-1700, International Light Technologies, Peabody, MA). Eight respirator coupons were placed on a horizontal surface so that the coupons and the sensor were approximately 6.2 cm below the lamps. A section of the filter cassette was attached to the radiometer's sensor head so that the irradiation of the sensor head would match that of the coupons. Calibration measurements using the radiometer showed that the eight positions' irradiance varied by no more than ±4%. Samples were also rotated once among the positions when they were flipped so that the mean exposures for the different groups were within ±0.1% of each other. The respirator coupons were exposed to 0, 120, 240, 470, or 950 J/cm^2^ of UV-C on each side (one side was exposed at a time). To expose the respirator straps, eight straps were laid side-by-side horizontally on a support surface at the same height as the sensor, and each side was exposed to 0, 590, 1180, or 2360 J/cm^2^ in a similar manner to the coupons. Temperature and humidity in the chamber were monitored using a humidity and temperature transmitter (HMT330). The mean temperature during coupon exposures was 27°C (SD 1.7), and the mean relative humidity was 25% (SD 6.5). The exposure system was controlled using a custom-written computer program. | - Particle penetration - Flow resistance - Bursting strengths - Breaking strength of the respirator straps | 3M-1860, 3M-9210, Gerson-1730 and Kimberly-Clark-46727. | None. | Ultraviolet germicidal irradiation had a small effect on filtration performance and essentially no effect on flow resistance at doses up to 950 J/cm^2^. At the same time, the structural integrity of the respirators showed a noticeable decrease at lower doses. The strength of the respirator straps was less affected by UVGI than the strength of the body material |
| Mills et al 2018 [56] | The custom UVGI device was made of polished aluminum (Alloy6061-T6 and Alloy 2024-T3; OnlineMetals.com, Seattle, WA) and measuring 40-in L×16-in W×13-in H with a tunnel extension measuring 18-in×8-in W×6-in H. The polished aluminum alloys were selected because they are UV reflective surfaces that do not alter the reflected light's wavelength. Eight 32-in 254-nm UV-Cbulbs with an irradiance of 0.39 W/cm^2^ at 1 m (Fresh-Aire UV) were incorporated into the device to deliver a UV dose of 1 J/cm^2^ in approximately 1 minute | - Remaining viable virus. | N95 FFR models: 3M 1860, 3M 1870, 3M VFlex 1805, Alpha Protech 695, Gerson 1730, Kimberly-Clark PFR, Moldex 1512, Moldex 1712, Moldex EZ-22, Precept 65-3395, Prestige Ameritech RP88020, Sperian HC-NB095, Sperian HC-NB295F, U.S. Safety AD2N95A. | Influenza A H1N1 virus. | Across all 180 FFRs tested, the mean UV dose per FFR was 1.1±0.1 J/cm^2^, the mean temperature was 21°C±2°C, and the mean relative humidity was 48%±6% within the UV device. For mucin-soiled straps, the mean viable virus recovered from UV-treated samples was statistically significantly lower than control samples for all FFR models tested except the VFlex 1805, Alpha Protech 695, Moldex EZ 22, and the U.S. Safety AD2N95A. The log reduction values observed for all mucin-soiled FFR straps were statistically significantly lower than their respective FFR facepieces. For sebum-soiled FFR facepieces, the mean viable influenza re-covered from control surfaces was 4.10±0.56 log TCID50; for sebum-soiled FFR straps, the mean viable influenza recovered from control surfaces was 3.90±0.65 log TCID50. The mean log reduction ranged from 1.25-4.64 log TCID50 for sebum-soiled facepieces and 0.08-4.40 log TCID50for sebum-soiled straps. The mean viable virus recovered from UV-treated samples was significantly lower for sebum-soiled facepieces than control samples for all FFR models tested. For sebum-soiled straps, the mean viable virus recovered from UV-treated samples was significantly lower than control samples for all FFR models tested except the 3M 1860, Alpha Protech, and Moldex EZ-22. The log reduction values observed for the sebum-soiled FFR straps were significantly lower than the respective FFR facepieces. |
| Lin et al 2018 [57] | An N95 FFR was placed 10 cm below an a6W handheld UV lamp (modelUVGL-58, VUP LLC, Upland, CA) that emitted a wavelength of 254nm (UVC, 18.9 mW/cm^2^) or 365nm (UVA, 31.2 mW/cm^2^). Both sides of each N95 FFR were exposed different times -1, 2, 5, 10and20 min-in a BSC. The UV intensity was measured using a handheld laser power and energy meter (OPHIR NOVA II, model Nova II PD300-UV) and was reported as a mean of five measurements over a 10X10mm aperture with a swivel mount and a removable filter | - Relative survival of *Bacillus subtilis spores* | N95 FFR (8210,3M, St. Paul, MN) | *Bacillus subtilis Spores* | This study found that UVC 254 nm had a strong decontamination effect, with a 100% bactericidal effect. No colony was recovered after exposure to UVC for as little as 5 min. However, relative survival remained above 20% after 20min of irradiation by UVA 365 nm, exponentially decaying with increased exposure time |
| Fischer et al 2020 [34] | Plates with fabric and steel discs were placed under an LED high power UV germicidal lamp (effective UV wavelength 260-285nm) without the titanium mesh plate (LEDi2, Houston, Tx) 50 135 cm from the UV source. At 50 cm, the UVAB power was measured at 5 μ W/cm^2^ using a General UVAB digital light meter (General Tools and Instruments New York, NY). Plates were removed at 10, 30, and 60 minutes and 1 mL of cell culture medium added. | - Mask Integrity was quantitatively determined using the Portacount Respirator fit tester - Disinfection of disposable | N95 respirators (3M Aura Particulate Respirator 9211+/37193) AOSafety N9504C respirators (Aearo Company Southbridge, MA). | HCoV-19 nCoV-WA1-2020 (MN985325.1) | UV inactivated SARS-CoV-2 rapidly from steel but more slowly on N95 fabric, likely due to its porous nature. Filtration performance of the N95 respirator was not markedly reduced after single decontamination. After two rounds of decontamination, the UV-treated masks retained comparable filtration performance to the control group and maintained acceptable performance after three rounds. |
| Liao et al 2020 [58] | Samples were placed into a UV sterilizer cabinet (CHS-208A), with a 254 nm, 8 W lamp, and 475 cm^2^ internal area. Samples were irradiated for 30 minutes and let to stand under ambient conditions for 10 minutes per cycle. Samples were either returned to the chamber for the next cycle or tested. | - Filtration efficiency  - Pressure drop | 3M 8210 (NIOSH N95), 4C Air, Inc. (GB2626 KN95), ESound (GB2626 KN95) and Onnuriplan (KFDA KF94). | None. | After three treatments of this method, the meltblown fabric still has characteristics similar to the initial sample. The UV-C light areal intensity distribution is not uniform inside the cabinet. Its exact value needs to be measured in the future for dose determination, as the necessary radiation to inactivate SARS-CoV was previously found to be above ~3.6 J/cm^2^. The data agrees with the NIOSH report at ten cycles but eventually decays to 93% at twenty cycles and makes it unsuitable for N95-grade FFRs. |
| Cadnum et al 2020 [59] | For each pathogen, 10-μL aliquots containing ~106 colony-forming units (CFU) or plaque-forming units (PFU) in phosphate-buffered saline were spread to cover steel disks 20 mm in diameter. The steel disk carriers were positioned 32 inches from a room decontamination device (Moonbeam 3) operated for a 90-second cycle or 180-second cycle for more UV-C resistant organisms. The relatively short cycle times were chosen to allow comparison in Log10 reductions. The disks were positioned at the height of 3 feet and oriented vertically parallel with the UV-C bulbs. Log10 CFU or PFU reductions were calculated by comparing recovery from UV-C-exposed carriers to untreated controls. | - To determine the efficacy against organisms on an N95 respirator. | 3M 1860 N95 Moldex 1517 N95 Kimberly-Clark 46727 N95 | Methicillin-resistant Staphylococcus aureus (MRSA) Bacteriophage Phi6 Bacteriophage MS2 | Bacteriophage MS2 and bacteriophage Phi6 were less susceptible to UV-C than vegetative bacteria such as MRSA but more susceptible than Candida species and C. difficile spores. Reductions in MRSA were consistently greater than reductions of the bacteriophages for most test sites. Reductions in MS2 and Phi6 were similar at most test sites. Differences in reduction across masks were statistically significant (F = 5.69, df = 2, P <0.01). Reductions were lower on the 3M 1860S N95 respirator. Reductions were also lower for the interior surfaces versus the exterior surfaces; it was noted that the exterior surfaces were impermeable to the liquid suspensions while the interior surfaces not. For the 3M 1860S N95 respirator, the reductions achieved on the exterior edge were lower than the exterior top surface; for this respirator, the edge has a fold that might result in shadowing that reduce UV-C delivery. The UV-C technologies did not meet pre-established criteria for decontamination under the test conditions used. |
| Ludwig-Begall et al 2020 [39] | Porcine respiratory coronavirus (PRCV strain 91V44) was passaged three times on confluent cell monolayers of the ST continuous cell line. A virus stock with a titer of 107.8 TCID50/mL was used. One negative control mask or respirator (not contaminated but treated), three treated masks or respirators (PRCV-contaminated and treated), and three positive controls (PRCV-contaminated but untreated), i.e., seven masks in total, were utilized. FFRs were individually irradiated using an LS-AT-M1 (LASEA Company, Sart Tilman, Belgium) equipped with 4 UV-C lamps of 5.5W (@UV-C). Hung vertically on a metal frame and FFRs were inserted into a safety enclosure. A 4 min UV-C irradiation (FFRs) led to a fluence of 5.2J/cm2 per mask. Power and irradiation time (120 s) were monitored and recorded. Following irradiation, FFRs were unloaded and placed in individual bags. | Disinfection of disposable with inactivating an infectious coronavirus | KN95 FFR – Guangzhou Sunjoy Auto Supplies CO. LTD, Guangdong, China (2020 N°26202002240270) | Porcine respiratory coronavirus (PRCV) | Recovery from FFR coupons yielded mean infectious virus at 6.96 × 104 (± 13.27) TCID50/mL for positive controls of the UV positive controls.  Recovery values for the infectious virus from FFR straps remained below the cell culture limit for UV detection. The cell culture limit of detection (LOD) was 0.8 log10 TCID50/mL (6.31 × 100). UV was shown to be insufficient to achieve viral inactivation by more than three orders of magnitude on a different lot of FFRs in a trial run (results not shown); a 4-min exposure time was thus tested for FFRs, reducing viral titers by over four orders of magnitude, from 6.96 × 104 (± 13.27) TCID50/mL to below the LOD. The impact of decontamination could not be measured for UV treated FFR straps due to insufficient recovery of infectious virus in positive FFR strap controls. |
| Ozog et al 2020 [60] | The low-pressure mercury lamp ultraviolet germicidal irradiation device (UVGI - 254 nm, 1 series) by Daavlin, with dimensions 22 in. × 10 in. × 8 in. to fit under the BSL3 biosafety hood. The irradiance was approximately 16.5 mW/cm^2^, a distance of 11.5 cm from the lamps approximately at the apex of the N95 respirator. The device used four lamps, spaced 4.5 cm apart. Intact FFRs in a donned position were inoculated on the outside-facing surface with a single 10 μL drop of viral stock (8 × 107 TCID50/mL) on four areas to account for differing received doses on complex surfaces: nosepiece, apex, chin-piece, and strap. Inoculated respirators were dried in a biosafety cabinet at room temperature for 40 min. For each N95 respirator model, FFRs were UVC-irradiated or left untreated as controls for viral load recovery. The respirators were then placed under the UVGI device, in the center, and were individually treated with a dose of 1.5 J/cm^2^. Then, they were rotated, and the wearer-facing side was irradiated with 1.5 J/cm^2^. The irradiation time for each side 60–70 s. After the completion of the irradiation, 4 mm circles containing the inoculated surface were obtained and were placed in 300 microliters of phosphate-buffered-saline for one hour at room temperature. Virus inoculation was performed on all five types of respirators. For each type, three were irradiated with UVC, and one was not irradiated to serve as a positive control. | - Virucidal efficacy | 3M 1860 (St.Paul, MN); 3M 8210 (St. Paul, MN); Moldex 1511 (Culver City, CA); 3M 8511 (St. Paul, MN); and 3M 9211(St. Paul, MN) | The SARS-CoV-2 strain used was USA-WA1/2020 NR-52281. | The dose of 1.5 J/cm2 to each side was an effective decontamination method for the facepieces of 3M 1860 and Moldex 1511 and the straps of 3M 8210 and the Moldex 1511. Some respirator models have materials, as the straps of the 3M 1860, with hydrophilic characteristics. Further, the facepieces of the 3M 8210 have hydrophilic properties, which were reflected in the reduced decontamination results, while the straps did not readily absorb the droplets, and hence were adequately disinfected. The Moldex 1511 facepiece and straps also appeared to be hydrophobic and did not absorb the droplets. Possible respirator-based solutions include a secondary disinfection step applied only to the straps. Further, ancillary disinfection testing was performed on the 3M 1860 straps using over-the-counter 70% isopropyl alcohol prep pads. The straps were inoculated with SARS-CoV-2 and wiped three times with the alcohol pad. Results showed that regardless of UVC irradiation, alcohol alone was sufficient to decontaminate the 3M 1860 straps. Some straps may require additional disinfection to maximize safety. Implementation of UVC decontamination methods requires careful consideration of model, material type, design, and fit-testing following irradiation. |
| Peltier et al 2020 [42] | Respirators were donned on a mannequin that was covered in a layer of soft closed-cell foam. The mannequin was installed in a 0.1-m3 exposure chamber and flooded with polydispersed combustion aerosol. Protocol 1- Surface. No. of Respirators Tested (10); Cycle Time (min) 10 (per side); Repeated Decontamination Treatments (1x, 10x). | Filtration efficiency, respiratory performance | 3M 1860 or 1860S models (3M, StPaul, MN). | None | UVGI degraded the respirator performance after 9 repeated cycles. |
| Simmons et al 2020 [61] | Ultraviolet C from a pulsed-xenon robot model (PXUV4D) was placed 1 m from the test surfaces inoculated with 0.020 mL of SARS-CoV-2. Chamber slides were exposed for 1, 2, or 5-minute durations, and the N95 respirator carriers were exposed for 5-minute durations. Viral titers were determined by plaque assay using a methylcellulose and crystal violet assay. | Disinfection of disposable | 3M 1860 | SARS-CoV-2 from isolate USA-WA1/2020, obtained from BEI resources | Inoculated N95 respirators exposed to 5 minutes of PX-UV showed <1.56 PFU/mL (log10), or a >4.79 log10 reduction (99.998%). PX-UV significantly reduces SARS-CoV-2 on N95 respirators with the potential to rapidly disinfectant environmental surfaces. |
| **3. Method: Ethylene oxide** | | | | | |
| Viscusi et al 2007 [29] | The two treatment conditions: EtO 3M Steri-Vac 4XL sterilizer processed in the warm cycle of 55° C and 883 mg/L ethylene oxide gas and EtO 3M Steri-Vac 5XL sterilizer processed in the warm cycle of 55° C and 725 mg/L ethylene oxide gas. Respirators tested using these treatments were shipped to and from a commercial facility specializing. All respirator samples were exposed to EtO for one hour, followed by a four-hour aeration interval The respirators were shipped back to the investigators and subsequently tested in house for filtration efficiency within 72 hours of receipt. TSI Model 8130 Automated Filter Tester (AFT) (TSI) was used to measure filter penetration. With room temperature with a continuous airflow of 85 L/minute. Particle penetration was determined using a cubic Plexiglas box placed between the filter chucks. | - Filtration performance | Two classes of FFRs were used; the N95 class and the P100 class. A single respirator model from the same manufacturer was selected for both FFR classes tested. | None. | For both treatments, the P100 straps were darkened slightly. The average penetration was slightly increased for both respirator models though not beyond NIOSH certification criteria. EtO 3M 5XL was found to be slightly less degrading than EtO 3M 4XL. EtO is a popular low-temperature decontamination method, and further study on FFRs is warranted. |
| Viscusi et al 2009 [30] | Steri-Vac 5XL sterilizer (3M, St Paul, MN, USA). Single warm cycle (55°C and 725 mg l-11100% EtOgas). FFRs and a chemical indicator placed in an individual standard poly/paper pouch. EtO exposure for 1 h followed by 4 h of aeration. FFRs were shipped to and from a commercial facility specializing in low-temperature sterilization methods and were tested within 72 h of receipt. | - Changes in physical appearance - Odor changes - Laboratory performance (filter aerosol penetration and filter airflow resistance) | Random sampling from those N95 FFR models presented in the US Strategic National Stockpile (SNS) and from models commercially available at the time of the study and included because they were considered more resistant to filtration efficiency degradation and thus offered a more rigorous basis of comparison. | None. | No visible changes were observed for all samples. |
| Salter et al 2010 [31] | Ethylene oxide (Amsco Eagle 3017): Triplicate FFRs of each model was exposed to EO in an Amsco Eagle 3017 EO sterilizer (Steris Corp., Mentor, Ohio)according to supplier directions, for 3 hr at 54◦C, followed by a12-hr aeration cycle at 54◦C. FFRs were packaged individually in sterilization pouches that contained sterilization indicators trips, which verified that the sterilizer performed adequately. Following the aeration period, each respirator was dismantled, and the respiratory components were weighed. Ten 14-mm-diameter samples were punched from areas equally spaced on each respirator, placed in separate Supelco 20-mL headspace vials, and weighed. Straps, nose cushions, and metal nosepieces were cut into∼12-mm pieces and weighed in individual headspace vials. GC-MS analysis for EO used guidance from the ISO standard AAMI/ANSI/ISO10993–7. | -The amount of residual chemicals created or deposited | NIOSH, FDA-approved N95, Surgical FFR, NIOSH-approved N95 and Particulate FFR | None. | The total ion chromatograms were examined in a window from 4.0–6.5 min because the time to elution of EO itself gradually decreased from∼5.6to5.2minastrimmingaway of contaminated sections at the front of the column progressively decreased its working length. This wide time window also accommodated chromatography variations, such as retention time shifts or peak fronting/tailing. No residual EO was detected in any of the respirators or respirator components tested. Diacetone alcohol was found in 11samples, 2-hydroxyethyl acetate (HEA) in 15 samples, and cyclohexanone in 2 samples. All 15 occurrences of HEA were on straps, and all gave an identifiable mass spectrum. However, all were measured in trace amounts (≤3 times the S/N of the baseline), so a more sensitive measurement of concentration will be needed before the significance of these traces can be evaluated. |
| Bergman et al 2010 [32] | Multiple (3-Cycle) of: Amsco® Eagle® 3017 100% EtO Sterilizer/Aerator (STERIS Corp., Mentor, OH) on HI-TEMP setting (55°C); 1-hr EtO exposure (736.4 mg/L) followed by 12-hr aeration. Samples were packaged in Steris Vis-U-All Low-Temperature Tyvek®/polypropylene-polyethylene Heat Seal Sterilization pouches (six samples per pouch with a chemical indicator strip). All samples were physically accommodated by a single EtO cycle. Samples were processed at a university medical center (one treatment per day in three consecutive days). The same pouch was used for all three treatments. | - Filtration performance - Physical - Integrity - Filter airflow resistance | Six models [three N95 FFR models (N95-A, N95-B, and N95-C) and three surgical N95 respirator models (SN95-D, SN95-E, and SN95-F)] | None. | 3X EtO treatments had similar mean % penetration to 1X treated samples tested previously for the same six models (no visible changes were observed for all samples). Filter Aerosol Penetration (%P) and Filter Airflow Resistance (mm H2O), respectively, for 3X, decontaminated masks:  N95-A (0.34± 0.03) and (8.0 ± 0.1)  N95-B (0.96± 0.13) and (12.0 ± 0.4)  N95-C (1.29 ± 0.40) and (11.9 ± 0.5)  SN95-D (2.55 ± 0.72) and (16.9 ± 0.6)  SN95-E (0.25 ± 0.09) and (9.5 ± 0.2)  SN95-F (0.75 ± 0.16) and (10.5 ± 0.4) |
| **4. Method: Dry heat** | | | | | |
| Viscusi et al 2007 [29] | Dry Heat Respirators were placed in a metal pan on racks of a Fisher Isotemp 500 Series (Fisher Scientific) laboratory oven at the temperature 160-180° C and turned over midway through the exposure period 60 minutes. 1st Test Condition Less aggressive 80° C. 2nd Test Condition More aggressive 160° C. TSI Model 8130 Automated Filter Tester (AFT) (TSI) was used to measure filter penetration. With room temperature with a continuous airflow of 85 L/minute. Particle penetration was determined using a cubic Plexiglas box placed between the filter chucks. | - Filtration performance | Two classes of FFRs were used; the N95 class and the P100 class. A single respirator model from the same manufacturer was selected for both FFR classes tested. | None. | At a temperature of 80° C, no visible changes were observed after 60 minutes for either type of respirator. However, a small increase in average penetration was observed for both the N95 and the P100. For treatment at 160° C, both respirators were largely melted and unusable after only 22 minutes. No further penetration tests were attempted using dry heat. |
| Viscusi et al 2009 [30] | To investigate the effects on filter aerosol penetration at various dry heat temperatures and to determine if these effects were similar to those of FFRs that underwent microwave oven irradiation, new FFRs were placed in a Fisher Scientific Isotemp 500 Series laboratory oven (Fisher Scientific, Pittsburgh, PA, USA) for 1 h at temperatures ranging from 80 to 120°C. Filter aerosol penetration was measured after samples cooled to ambient temperature. | - Changes in physical appearance - Odor changes - Laboratory performance (filter aerosol penetration and filter airflow resistance) | Random sampling from those N95 FFR models presented in the US Strategic National Stockpile (SNS) and from models commercially available at the time of the study and included because they were considered more resistant to filtration efficiency degradation and thus offered a more rigorous basis of comparison. | None. | Five SN95-D FFRs melted, one at 100°C, two at 110°C, and two at 120°C and could not be penetration or airflow resistance tested. |
| Fischer et al 2020 [34] | Plates with fabric and steel discs were placed in a 70°C oven. Plates were removed at 10, 20, 30, and 60 minutes and 1 mL of cell culture medium added. | - Mask Integrity was quantitatively determined using the Portacount Respirator fit tester - Disinfection of disposable | N95 respirators (3M Aura Particulate Respirator 9211+/37193) AOSafety N9504C respirators (Aearo Company Southbridge, MA). | HCoV-19 nCoV-WA1-2020 (MN985325.1) | The heat caused more rapid inactivation on N95 than on steel; inactivation rates on N95 were comparable to UV. Filtration performance of the N95 respirator was not markedly reduced after single decontamination. |
| Liao et al 2020 [58] | Samples were loaded into a pre-heated 5-sided heating chamber (Across International, LLC) for the temperatures and times given in the main text. The dry heat was applied using the Across International vacuum heating oven under ambient conditions. In the case of the SH-642, the humidity was set the lowest value (30% RH up to 85 °C. Above 85 °C the humidity is <30% but cannot be controlled). High humidity (100% RH) was simulated via sealing meltblown fabrics, or FFRs, inside a polyethylene bag with 0.3 mL of water and placing them inside the SH-642 chamber. The resting time between cycles was 10 minutes for the 75 °C and 85 °C treatments and 5 minutes for the 100 °C and 125 °C treatments. After resting, the samples were returned to the chamber to begin the next cycle. They initially chose 75 ℃ due to blanket warming ovens in hospital environments that can reach ~80 ℃. Further experiments used 85 ℃ if 75 ℃ is not enough to inactivate SARS-CoV-2. | - Filtration efficiency  - Pressure drop | 3M 8210 (NIOSH N95), 4C Air, Inc. (GB2626 KN95), ESound (GB2626 KN95) and Onnuriplan (KFDA KF94). | None. | Performed multiple humidity experiments (30%, 70%, and 100% RH) at 85 °C (20 minutes/cycle), observing no appreciable degradation of efficiency at any humidity level. At 85°C, 30% RH, observed no efficiency degradation over fifty cycles on a meltblown fabric. Using less harsh conditions (75 °C, dry heat), the results are expectedly in agreement. Testing conditions for the FFRs were under a flow rate of 85 L/min. From all the FFRs, it was observed little change in the filtration properties, as all FFRs with filtration efficiency >95% could retain filtration efficiencies >95% after 20 cycles of heat treatment, even in a humid environment. There is little to no change in the filtration efficiency and pressure drop up to 100 °C in low moisture conditions. However, at 125 °C, there is a sharp drop in filtration efficiency while maintaining a constant pressure drop around cycle 5. Concluded that the highest subjectable temperature to the FFR for repeated use with ≥95% efficiency is <100 °C. At temperatures ≤85 °C, humidity does not play a crucial role in the filtration properties, as FFRs tested at a near 100% RH at 85 °C were unaffected. However, as steam results in a decrease in efficiency, the humidity should be kept low if approaching 100 °C. |
| Pascoe et al 2020 [62] | A laboratory MINI/6 incubator (Genlab Ltd) was used to provide dry heat and was set to 70°C. The temperature was monitored with a glass thermometer whilst relative humidity was monitored with a digital hygrometer (Traceable; Fisher Brand). Relative humidity remained below the lower limit of detection of this device (25%) throughout the experiments. Samples were placed in the dry heat for 5–90 min. Inoculated membranes were placed on a folded N95 respirator as described above, and the respirator placed on a shelf in the center of the incubator. | - Effect of essential oils on filtration efficiency. - Bacterial filtration efficiency via aerosol nebulization. - Assessment of mask NaCl filtration efficiency. | FFP2/N95-type respirators (Kimberly-Clark; Fluidshield) | *Staphylococcus aureus* | 70°C/<25% relative humidity:  Time 30 min Log10 reduction (S. aureus) = 1.77 SD 0.54 Time 60 min Log10 reduction (S. aureus) = 3.50 SD 0.34 Time 90 min Log10 reduction (S. aureus) = 4.49 SD 0.57 Bacterial filtration efficiency (%): ≥99.9%: Dry heat reprocessed once: Log10 cfu recovery from sterile discs = ≤0.30 SD ±0.00 ≥99.9%: Dry heat reprocessed three times = ≤0.30 SD ±0.00  There were no detectable changes in aerosol filtration efficiency, even after three reprocessing cycles. |
| Daeschler et al 2020 [63] | Thermal disinfection in cycles of 60 minutes at 70°C at either 0% relative humidity. To control temperature and relative humidity, they set the BevLes Heated Holding Cabinet with humidity (BevLes Inc.) to 70°C and varied the humidity between 0% and 50% relative humidity. They used a digital thermal- and hygrometer (Hagen Group Inc., Canada) as an added quality-control measure. Additionally, they accounted for potential real-world temperature fluctuations by cooling the masks to room temperature for 5 minutes mid-cycle. | - Inactivation of SARS-CoV-2  - Bacterial inactivation  - Microstructural analysis of the N95 filter layer  - Respirator fit testing, filter efficiency, and breathing resistance testing | N95 respirators (8110s, 9105s, 8210 and 1860s; 3M). | *SARS-CoV-2 Escherichia coli* | After dry heat treatment (70°C for 60 min), no infectious SARS-CoV-2 could be detected in any of the previously virus-inoculated respirators. In contrast, high levels of SARS-CoV-2 could still be detected in respirators that had not undergone heat treatment. In samples exposed to dry heat (70°C/0% relative humidity), more than 1000 bacterial colonies of E. coli were still detectable. Even after 10 cycles of thermal disinfection of 60 minutes at 70°C at either 0% relative humidity, the mean overall fiber diameter remained within the range for unprocessed N95 filters as specified in the US patent. All tested groups of thermally disinfected respirators significantly exceeded the fit factor of 100. The OSHA-defined standard pass value for sufficient respiratory protection (p < 0.001 for all groups), and so did the lower bound of their 99% CIs. None failed the test in a total of 138 performed quantitative fit tests with disinfected respirators (0% relative humidity). The decontaminated respirators' subjective fit and wearing comfort did not differ from new masks and were rated. The disinfected respirators significantly exceeded 95% filtration efficiency after 5 and 10 disinfection cycles (p < 0.001). The breathing resistance of the same set of disinfected respirators was significantly lower than the maximum tolerable resistance standard of 343.23 Pa for all tested groups (p < 0.001). |
| Ludwig-Begall et al 2020 [39] | Porcine respiratory coronavirus (PRCV strain 91V44) was passaged three times on confluent cell monolayers of the ST continuous cell line. A virus stock with a titer of 107.8 TCID50/mL was used. One negative control mask or respirator, three treated masks or respirators (PRCV-contaminated and treated), and three positive controls (PRCV-contaminated but untreated), i.e., seven masks in total, were utilized. FFRs hung horizontally on a metal frame were inserted into an electrically heated vessel (M-Steryl) for treatment with temperatures of 102°C (± 4°C) for 60 min (± 15 min). Temperatures inside the heated vessel were recorded throughout to ensure correct exposure conditions. After the termination of the treatment cycle, FFRs were allowed to cool and then bagged individually. | Disinfection of disposable | KN95 FFR – Guangzhou Sunjoy Auto Supplies CO. LTD, Guangdong, China (2020 N°26202002240270) | Porcine respiratory coronavirus (PRCV) | Recovery from FFR coupons yielded a mean infectious virus at 2.16 × 104 (± 3.28) TCID50/mL for positive controls of the dry heat assays.  Recovery values for the infectious virus from FFR straps remained below the cell culture limit of detection for dry heat positive controls. The cell culture limit of detection (LOD) was 0.8 log10 TCID50/mL (6.31 × 100). FFR coupons decontaminated via dry heat reduced viral titers by over four orders of magnitude (2.16 × 104 (± 3.28) TCID50/mL. The impact of decontamination could not be measured for dry heat-treated FFR straps due to insufficient recovery of infectious virus in positive FFR strap controls. |
| Cadnum et al 2020 [59] | Examined the effectiveness of heating N95 respirators to 70°C for 30 minutes in an oven (Economy Incubator, Precision). N95 respirators were contaminated with MRSA and the bacteriophages by pipetting onto 3 different respirator areas, and log10 reductions were calculated. | - To determine the efficacy against organisms on an N95 respirator. | 3M 1860 N95 Moldex 1517 N95 Kimberly-Clark 46727 N95 | *Methicillin-resistant Staphylococcus aureus (MRSA) Bacteriophage Phi X174 Bacteriophage Phi6 Bacteriophage MS2* | The Dry heat at 70°C for 30 minutes had limited effectiveness against bacteriophages MS2 and Phi6 versus methicillin-resistant Staphylococcus aureus (<1 log10 plaque-forming units versus >4 log10 colony-forming units respectively, F = 54.7, df = 2, P< 0.01). |
| Li et al 2020 [64] | The 10-mL aliquots containing 106 colony-forming units or plaque-forming of the test organisms suspended in 8% simulated mucus were inoculated onto 1-cm 2 areas on both the outer or inner surfaces of the respirators or face masks. Inoculated masks or respirators were subjected to dry heat at 100°C for 15 minutes in an oven (Thermo Fisher Scientiﬁc; Waltham, MA). After treatment, the inoculated sections of the face masks and N95 respirators were vortexed for 1 minute in 1 mL of phosphate-buffered saline with 0.02% Tween and plated quantify organisms. The tests were performed in triplicate. A reduction of 3-log10 or greater in the recovery of organisms inoculated was considered effective for decontamination. | - Decontamination | 3M 1860 N95 respirators (3M; Saint Paul, MN) | *- Methicillin-resistant Staphylococcus aureus (MRSA) - Nonenveloped single-stranded RNA virus bacteriophage MS2* | Dry heat at 100°C for 15 minutes did not result in a greater than 3 log10 reduction of either organism at any inoculated sites on any masks or respirators. No visible changes were observed in any of the masks or respirators after 5 cycles of decontamination. |
| Li et al 2020 [65] | Ten-μL aliquots containing ∼106 colony-forming units (CFU) or plaque-forming units of the test organisms suspended in 8% simulated mucus were inoculated onto 1-cm^2^ areas on both the outer or inner surfaces of the respirators and face masks. The inoculated masks and respirators were subjected to 100°C steam treatments of 2, 10, or 30 seconds by placing them inside a steamer for the specified time during the steam cycle. After treatment, the inoculated sections were cut out and processed to quantify viable organisms. All tests were performed in triplicate. Log10 reductions were calculated in comparison to untreated controls. A reduction of 3-log10 or greater was considered effective for decontamination. To assess the impact on respirator performance, qualitative and quantitative (Portacount Respirator Fit Tester) fit testing was performed before and after N95 respirators were subjected to 20-30-second steam treatments. | - Decontamination  - Fit test | 3M 1860 N95 respirators (3M; Saint Paul, MN) | *Methicillin-resistant Staphylococcus aureus (MRSA), Geobacillus stearothermophilus spores, and the nonenveloped, single-stranded RNA virus bacteriophage MS2.* | The 10- and 30-second steam treatments met the decontamination criteria of bacteriophage MS2 and MRSA on N95 respirators, whereas the 2-second treatment did not. The steam treatments did not substantially reduce G. stearothermophilus spores. N95 respirators passed fit testing after 20- 30-second steam treatments. After steam treatment, the respirators were slightly damp. All 30 used medical procedure masks cultured were contaminated with bacteria with an average of 2.4 log10 CFU recovered, predominantly Streptococcus species and coagulase-negative staphylococci. Staphylococcus aureus was recovered from 3 (10%) masks. The 30-second steam treatment eliminated all bacteria from 29 of 30 (97%) masks. Steam treatment resulted in the rapid decontamination of bacteriophage MS2 and MRSA on N95 respirators and medical procedure masks. The reductions in bacteriophage MS2 met the current Food and Drug Administration Enforcement Policy for Face Masks and Respirators of a >3 log10 reduction of viruses. Still, the requirement for a >6 log10 inactivation of bacterial spores was not met. |
| Xiang et al 2020 [66] | A microbial aerosol generator containing bacteria was pumped through surgical masks and N95 respirators. Two of the inoculated masks for each bacteria strain were placed in a steel box and heated at 60°C and 70°C for 1 hour in an electric oven. Three pieces of a mask were taken and inoculated with the cell suspension infected by H1N1. Two 25 cm2 piece was heated at 60°C or 70°C for 1 hour respectively. An unheated piece was used as the control. They used a 3M Qualitative Fit Test Apparatus FT-30 to assess the fit of N95 respirators after being heated. Filtration efficiency - measuring the filtration rate of live bacteria in aerosols. Bacterial colonies on the petri dish were counted after 48-hour cultivation. | - Decontamination. - Fit test and filtering efficiency | 3M 1860 | Escherichia coli (ATCC25922), Staphylococcus aureus (ATCC25923), Pseudomonas aeruginosa (ATCC27853), Klebsiella pneumonia (ATCC70063), Acinetobacter baumannii (ATCC17978), Corynebacterium pseudodiphtheria (ATCC10701), Candida albicans (ATCC10231) and H1N1 strain (A/Zhejiang/1/2009[H1N1]) | After being heated, the N95 respirators and surgical face masks showed no changes in their shape and components; the filtering efficacies of the N95 respirators for bacterial aerosols were 98%, 98%, and 97% after 1, 2, and 3 hours of heating, respectively. This practice is suitable for use at home for up to at least three rounds of dry heat. |
| **5. Method: Moist heat / Pasteurization** | | | | | |
| Bergman et al 2010 [32] | Multiple (3-Cycle) of: Moist heat incubation / pasteurization (MHI) - 30-min incubation at 60°C, 80% RH in a Caron model 6010 laboratory incubator (Marietta, OH). Following the first incubation, the samples were removed from the incubator and air-dried overnight. Following the second and third incubations, samples were removed from the incubator and air-dried for 30 min with the aid of a fan. | - Filtration performance - Physical Integrity - Filter airflow resistance | Six models [three N95 FFR models (N95-A, N95-B, and N95-C) and three surgical N95 respirator models (SN95-D, SN95-E, and SN95-F)] | None. | Moist heat incubation/pasteurization (MHI) caused all SN95-E samples to experience the partial separation of the inner foam nose cushion from the FFR. Two of the SN95-D samples experienced a slight melting of the head straps following the first 2-minute cycle. |
| Viscusi et al 2011 [51] | Moist heat incubation (MHI) - FFRs incubated for 30 min at 60ºC (upper temp. limit), 80% RH in a Caron Model 6010 laboratory incubator (Marietta). Following treatment, FFRs dried overnight on a laboratory benchtop. | - Tolerability. - Usability (specifically, odor, comfort, or donning ease). | (1) 3M 8000, (2) 3M 8210, (3) Moldex 2200, (4) 3M 1860, (5) 3M 1870, and (6) Kimberly Clark PFR95–270. | None. | Test technicians did not report any unusual or strong odor from FFRs following the decontamination method. The 3M Model 1870 (3M, St. Paul, Minn.) samples experienced a slight separation of the inner foam nose cushion (some to a lesser or greater degree) from the FFR body following moist heat incubation (MHI) treatment. |
| Bergman et al 2011 [52] | 15 min incubation at 60 °C (upper temp. limit), 80 % RH in a Caron model 6010 laboratory incubator (Marietta). | - respirator fit (odor, comfort, or donning ease) | Three models of surgical N95 respirators (3M 1860, 3M 1870, and Kimberly Clark PFR95-270 | None | Did not cause significant changes in respirator fit. Treatment caused the 3M 1870 samples to experience a slight separation of the inner foam nose cushion (some to a lesser or greater degree) from the FFR body; however, multiple treatments were not noticed to cause a more pronounced separation as compared to a single treatment. |
| Heimbuch et al 2011 [50] | A 6-L sealable container (17 cm h X 19 cm wX 19 cm l) was filled with 1 L of tap water for warm moist heat. A plastic support rack was placed in the water to isolate the FFR from the liquid. Before the test, the container was warmed in an oven to 65ºC for a minimum of 3 hours. The container was removed from the oven, and an H1N1-contaminated FFR was placed on the rack. The containers were sealed and returned to the oven for 30 minutes. | - Inactivation of the H1N1 virus | 6 FFR models (3 particulate, designated P1-P3, and 3 surgical, designatedS1-S3). | H1N1 influenza virus. | Provided an average >4-log reduction of viable H1N1 influenza virus against both the droplet and aerosol challenges for 5 FFRs, but not on the P1 FFR model. No other FFRs showed noticeable deterioration or deformation. |
| Lore et al 2012 [53] | A 6-l sealable container (19X19X17cm) was filled with 1l of tap water, placed in an oven (Thermo Fisher Scientific) heated to 65±5°C for 3h. The container was removed from the oven for testing, and a single virus-contaminated respirator was placed on the rack. The container was opened for each decontamination procedure, and the FFR placed onto the rack with the convex surface pointed toward the water layer. The container was then sealed and returned to the oven for the 20-min treatment. | - Virucidal capability. - Post-decontamination filter performance | 3M models 1860s and 1870 | Influenza A H5N1 virus. | This study showed that the moist heat, satisfactorily decontaminated the 3M1860s and 1870 FFRs as measured by a virus culture method. |
| Liao et al 2020 [58] | Three samples were stacked on top of a beaker with boiling water inside (around 15 cm above the water). The samples were left on top of the beaker and steamed for ten minutes. Afterward, they were left to air dry completely. Samples were either tested or placed back on top of the beaker to continue the next treatment cycle. | - Filtration efficiency  - Pressure drop | 3M 8210 (NIOSH N95), 4C Air, Inc. (GB2626 KN95), ESound (GB2626 KN95) and Onnuriplan (KFDA KF94). | None. | After three treatments of this method, the meltblown fabric still has characteristics similar to the initial sample. However, after five steam treatments, the efficiency has a sharp drop, which continues at cycle 10. Filtration efficiency based on steam treatment in a meltblown after cycle 5 has a significant drop. Steam clearly had degradation in the filtration efficiency and pressure drop after ten-cycles of treatment. |
| Daeschler et al 2020 [63] | Thermal disinfection in cycles of 60 minutes at 70°C at 50% relative humidity. To control temperature and relative humidity, they set the BevLes Heated Holding Cabinet with humidity (BevLes Inc.) to 70°C and varied the humidity between 0% and 50% relative humidity. They used a digital thermal- and hygrometer (Hagen Group) as an added quality-control measure. Additionally, they accounted for potential real-world temperature fluctuations by cooling the masks to room temperature for 5 minutes mid-cycle | - Inactivation of SARS-CoV-2 - Bacterial inactivation - Microstructural analysis of the N95 filter layer - Fit testing, filter efficiency, and breathing resistance testing | N95 respirators (8110s, 9105s, 8210 and 1860s; 3M). | *SARS-CoV-2 Escherichia coli* | A single heat treatment rendered SARS-CoV-2 undetectable in all mask samples. Compared with untreated inoculated control masks, E. coli cultures at 24 hours were virtually undetectable from masks treated at 70°C and 50% relative humidity (optical density at 600 nm wavelength, 0.02 ± 0.02 v. 2.77 ± 0.09, p < 0.001), but contamination persisted for masks treated at lower relative humidity. Even after 10 cycles of thermal disinfection of 60 minutes at 70°C at either 50% relative humidity, the mean overall fiber diameter remained within the range for unprocessed N95 filters as specified in the US patent. All tested groups of thermally disinfected respirators significantly exceeded the fit factor of 100. None failed the test in a total of 138 performed quantitative fit tests with disinfected respirators (50% relative humidity). The subjective fit and wearing comfort of the decontaminated respirators did not differ from new masks. Further, they tested the particle filtration efficiency and breathing resistance in the same 4 types of commercially available N95 respirators that underwent 5 cycles or 10 cycles of thermal disinfection at 50% relative humidity. The disinfected respirators significantly exceeded 95% filtration efficiency after 5 and 10 disinfection cycles (p < 0.001). Besides, the breathing resistance of the same set of disinfected respirators was significantly lower than the maximum tolerable resistance standard of 343.23 Pa for all tested groups (p < 0.001). |
| Ma et al 2020 [67] | The strain of avian infectious bronchitis virus H120 was used to mimic SARS‐CoV‐2. A device was made with a seamless plastic bag with syringes, which collected the aerosols containing the virus produced with a nebulizer. Four brands of medical masks and two brands of N95Ms produced in China were tested. These masks were put into intact plastic bags and steamed on boiling tap water in a kitchen pot for a certain time. RNA was extracted for the detection of the amount of the virus using the TaqMan RT-PCR. The blocking efficacy before and after steaming was detected this way: in brief, the top parts of 60‐mL syringes were removed, and then they were wrapped with the tested masks except for the control tubes. A facial cleaning sponge (8‐mm thick) made of hydrophilic polyvinyl alcohol was set inside the syringe in advance behind the wrapped mask for collecting the virus passing through the masks. Three or four syringes were then aligned and bound seamlessly together to generate paired data. These syringes were connected with a seamless plastic bag, which collected the aerosols containing the virus produced with a nebulizer. The aerosols have a median diameter of 3.9 μm, and 65% of the aerosols have diameters less than 5.0 μm, as given in the specifications of the nebulizer. The masks were steamed in boiling water to examine whether the masks' blocking efficacy declined significantly due to steam decontamination. | - Blocking efficacy of steamed masks | Two brands of N95Ms (N95Ma, N95Mb) produced in China were tested. Their brand names were not showed to avoid conflict of interest. | Avian infectious bronchitis virus H120 | To examine whether the masks' blocking efficacy declined significantly due to steam decontamination, the masks were steamed in boiling water. Some N95 had been used for 7 days before steam treatment, and the remaining brands had not been used before the steam treatment. All the Ct values of masks, used or unused, were of no significant difference after being steamed for 20, 60, or 120 minutes (P > .05). Accordingly, their blocking efficacy changed little due to the hot steam. |
| Anderegg et al 2020 [68] | The masks were subjected to 5 cycles at 85ºC + 60-85% humidity. Each N95 FFR was placed into a plastic container with a paper towel with 500 uL of water. Both the mask and container were labeled with a black permanent ink marker, which would allow the mask to be identified by the correct healthcare worker and avoid cross-contamination. For testing purposes, the lid of each container was modified to have a temperature and humidity sensor | - Fit - Filtration efficiency - Pressure drop | 3M 1860, 3M 1870, 3M 8210 Plus, Chen Heng V9501 KN95, HKYQ N95 | None. | All three respirators pass quantitative fit testing (score of >100) and show no degradation of mask filtration efficiency. The Chen Heng V9501 KN95 and HKYQ N95 finding no degradation of mask filtration efficiency; however, even for unheated masks, these scored <50 for every fittest. The heating method presented here is scalable from individual masks to over a thousand a day with a single industrial convection oven, making this method practical for local application inside health-care facilities. |
| **6. Method: Ethanol** | | | | | |
| Lin et al 2017 [69] | 10 min submersion in 70% ethanol solution. Following each exposure, masks were placed in a laboratory chemical hood and allowed to air-dry overnight before performing the laboratory aerosol filtration test. | - Penetration of particles through mask. - Pressure drop. - Filter quality. | Not specified N95. | None. | Before decontamination, the particle penetration through the N95 mask did not exceed 5%. Submersion in 70% ethanol solution significantly changed the penetration through the N95 masks to more than 30%. |
| Lin et al 2018 [57] | Ethanol with various concentrations and volumes was added to the center of the surface of the N95 FFR using a pipette. The FFR was then dried in a petri dish placed in a biosafety cabinet (BSC) for 10 min. | - Relative survival of Bacillus subtilis spores | N95 FFR (8210,3M, St. Paul, MN) | *Bacillus subtilis Spores* | Relative survival of 89+6% was obtained after spiking with 50% ethanol, and 73+5%was obtained after spiking with 70% ethanol. The lowest RS of 68+3% was obtained when the concentration of ethanol was 80%. The result obtained using 95% ethanol (RS=73+7%) was close to that obtained using 70% ethanol, although the samples that were spiked with 95% ethanol sometimes yielded slightly higher values of RS than were obtained using the 80% ethanol samples. Relative survival of 59+8%was obtained in24h without decontamination. The 50%, 70%, 80%, and 95% ethanol-treated samples had relative survival values of33+8%,22+8%, 20+2%and 26+7% after24 h, respectively. Just after spiking with ethanol, the RS was found to have declined from 100% to 68-75%. When 0.4 ml (αaq=0.23) of 70% ethanol was applied, the RS fell to 22% in 24 h. The relative survival fell to 20% when 80% of ethanol was used. |
| Fischer et al 2020 [34] | Fabric and steel discs were placed into the wells of one 24 well plate per time-point and sprayed with 70% ethanol to saturation. The plate was tipped to nearly vertical, and 5 passes of ethanol were sprayed onto the discs from approximately 10 cm. After 10 minutes, 1 mL of cell culture medium was added. | - Mask Integrity was quantitatively determined  - Disinfection of disposable | N95 respirators (3M Aura Particulate Respirator 9211+/37193) AOSafety N9504C respirators (Aearo Company Southbridge, MA). | HCoV-19 nCoV-WA1-2020 (MN985325.1) | Ethanol yielded extremely rapid inactivation both on N95 and on stainless steel. Filtration performance of the N95 respirator was not markedly reduced after single decontamination. Subsequent rounds of decontamination caused sharp drops in the filtration performance of the ethanol-treated masks. |
| Liao et al 2020 [58] | Samples were immersed into a solution of 75% ethanol, and left to air dry (hanging) and subsequently tested. | - Filtration efficiency.  - Pressure drop. | 3M 8210 (NIOSH N95), 4C Air, Inc. (GB2626 KN95), ESound (GB2626 KN95) and Onnuriplan (KFDA KF94). | None. | The first disinfection noted that the ethanol drastically degraded the filtration efficiency to unacceptable levels, while the pressure drop remained comparable. |
| Grinshpun et al 2020 [70] | To simulate contamination of facepieces, they were soiled with a protein of egg whites derived from commercial eggs were dialyzed against de-ionized distilled (Milli-Q) water. Protein solution containing 1 mg of egg whites in 3 mL of water was sprayed on masks/respirators before their sterilization in an autoclave. When multiple sterilizations were applied, facepieces were soiled before each autoclave treatment to mimic the device usage in air environments contaminated with protein that may be associated with emission of pathogenic virions by infected persons—treatment of facepieces by soaking in 70% ethanol for 2 h. A 70% ethanol solution was prepared by diluting a 200 Proof pure ethanol (Decon Laboratories) with distilled deionized (Milli-Q) water. | - efficiency, pressure drop, damage | The FFRs were both from 3M Corp.: Model 8210 and Model 1870. | none | The N95 filter that demonstrated superior initial performance, Ec ≥99%, did not even meet the 95% efficiency requirement after the treatment. It was observed for any tested particle size and flow rate. The initial air pressure drop through the N95 filter was moderate (3.1 mm wg at 30 L/min and 12.7 mm wg at 85 L/min). For the N95 FFR, the post-treatment pressure drop decreased by 13–35%, depending on the flow rate and decontamination method applied. |
| **7. Method: Isopropanol solution** | | | | | |
| Viscusi et al 2007 [29] | Henry Shein Isopropyl alcohol (IPA), 70%. Based on the treatment vessel's capacity, either 4 or 8 respirator samples were submerged into a dishpan or 4L beaker containing 3-5 liters of treatment solution. When needed, a second dishpan was used to keep respirators submerged for the time interval the 10 to 30 minutes. 1st Test Condition Less aggressive: dunk 1 second. 2nd Test Condition More aggressive: dunk 1 minutes. Respirators were removed, hung on a pegboard, and air-dried for 72 hours before filter penetration testing. TSI Model 8130 Automated Filter Tester (AFT) was used to measure filter penetration (Pen). With room temperature with a continuous airflow of 85 L/minute. Particle penetration was determined using a cubic Plexiglas box placed between the filter chucks. | - Filtration performance | Two classes of FFRs were used; the N95 class and the P100 class. A single respirator model from the same manufacturer was selected for both FFR classes tested. | None. | Fading of strap ink was the only visible change observed. As expected, both treatment conditions (1 second and 1-minute submersion) resulted in markedly increased average penetration for both respirator classes. This was possibly due to changes in the density and/or spatial distribution of the electret charges on the polymer fibers' surface by the liquid phase application. |
| Lin et al 2017 [69] | 10 min submersion in 100% isopropanol solution. Following each exposure, masks were placed in a laboratory chemical hood and allowed to air-dry overnight before performing the laboratory aerosol filtration test. | - Penetration of particles through mask. - Pressure drop. - Filter quality. | Not specified N95. | None. | Treatment of N95 masks with isopropyl increases the penetration of particles larger than 50 nm, owing to a reduction in the filter's charge density. |
| **8. Method: Microwave oven** | | | | | |
| Viscusi et al 2007 [29] | Exposures were carried out in a standard commercially 2,450-MHz microwave oven, Sharp Model R-305KS (Sharp Electronics, Mawwah, NJ) with a revolving glass carousel. Although rated at 1100W on the 100% full power setting, they obtained an average power measurement of 750 W/ft3 from four evaluations at various evenly spaced representative locations in the oven using the power determination method recommended by the manufacturer. In both treatments (2 min. and 4 min.) the samples were irradiated for half the time, promptly turned over, and irradiation was repeated for the remainder of the allotted time. TSI Model 8130 Automated Filter Tester (AFT) (TSI, Inc., St. Paul, MN) was used to measure filter penetration. With room temperature with a continuous airflow of 85 L/minute. Particle penetration was determined using a cubic Plexiglas box placed between the filter chucks. | - Filtration performance | Two classes of FFRs were used; the N95 class and the P100 class. A single respirator model from the same manufacturer was selected for both FFR classes tested. | None. | For treatments of two-minute duration, no visible changes were observed. Average penetration was unchanged for P100 respirators, and N95 average penetration was increased slightly. After 4 minutes of microwave exposure, the N95 filter media melted at the aluminum nosebands' ends and formed visible holes. N95 filter penetration was significantly increased. For one P100 sample tested, the face seal was melted when the face seal was placed face-down on the circular glass plate for the second half of the treatment. The melted sample was not tested. The P100 penetration was somewhat increased and significantly more variable. Thus, selecting appropriate parameters for microwave decontamination of FFRs is critical, and future work in this area is warranted, given the ubiquity and relative ease of operating a microwave oven. |
| Viscusi et al 2009 [30] | Commercially available 2450 MHz, Sharp Model R-305KS microwave oven with revolving glass carousel, 1100 W; 750 W ft-3 experimentally measured; 2-min total exposure (1 min each side or FFR). A paper towel was placed on the revolving glass plate for insulation to protect the FFRs from melting onto the glass plate. Using a power setting of 10 (maximum power), FFRs were placed face seal-side down, initially, to reduce the risk of face seal component materials melting onto the paper towel due to elevated temperatures reached by the glass plate when microwaved for 2 min. Ambient cooling of the glass plate was maintained between trials | - Changes in physical appearance - Odor changes - Laboratory performance (filter aerosol penetration and filter airflow resistance) | Random sampling from those N95 FFR models presented in the US Strategic National Stockpile (SNS) and from models commercially available at the time of the study and included because they were considered more resistant to filtration efficiency degradation and thus offered a more rigorous basis of comparison. | None. | SN95-E filtration material melted in areas adjacent to the metallic nosebands. SN95-E was considered unwearable following treatment and subsequently were not evaluated for filter aerosol penetration or filter airflow resistance. |
| Fisher et al 2009 [71] | Steam treatment times of 0, 15, 30, 45, 60, and 75 s were tested in triplicate for efficacy against MS2. Steam sterilization chambers were constructed from plastic pipette tip boxes. Nine holes that were approximately 4 mm in diameter were drilled in the top of each pipette tip box lid for ventilation. The base of the pipette tip box was filled with 50 ml of room temperature water. Respirator coupons were loaded with MS2, as described above. After MS2 loading, the coupons were placed on a rack inside the steam sterilization chamber approximately 2.5 cm above the water level. The vented pipette tip box lid was placed over the base, and the chamber was heated in a microwave oven on high for the appropriate treatment time. After treatment, the coupons were carefully removed from the chamber and placed in 10 ml of ATCC medium 271 in conical tubes. The tubes were capped and vortexed for 45 s. The coupons were removed and discarded, and each suspension was assayed by performing a plaque assay. | - Virucidal efficacy | NIOSH-certified N95 | Coliphage MS2 (ATCC 15597-B1) | Treatments of 45 seconds and longer for both low-protective-factor (LPF) and high-protective-factor (HPF) media resulted in log reduction (LR) which reached the detection limits. |
| Bergman et al 2010 [32] | Multiple (3-Cycle) of: Commercially available 2,450-MHz, Sharp Model R-305KS (Sharp Electronics, Mahwah, NJ) microwave oven with revolving glass carousel, 1,100 W (manufacturer rated); 750 W/ft3 experimentally measured; 2-min total exposure duration at a power setting of 10 (maximum power). Two pipette tip boxes placed side-by-side (each 11.7 cm x 8.0 cm x 5.0 cm) filled with 50 mL room-temperature tap water (~ 20°C). FFR is placed outer-side down on top of pipette-tip boxes. FFR samples dried 1 hr between each exposure. | - Filtration performance - Physical Integrity - Filter airflow resistance | Six models [three N95 FFR models (N95-A, N95-B, and N95-C) and three surgical N95 respirator models (SN95-D, SN95-E, and SN95-F)] | None. | 3X Microwave oven generated steam (MGS) all SN95-E samples to experience the partial separation of the inner foam nose cushion from the FFR. Two of the SN95-D samples experienced a slight melting of the head straps following the first 2-minute cycle. Some concerns have been raised about possible sparking during microwave heating caused by the metallic FFR nose bands. In these experiments where water basins were placed in the microwave with the FFR, no sparking was observed. Sparking has previously been observed only once in our laboratory when microwaving an FFR for one minute without using a water basin. |
| Viscusi et al 2011 [51] | Commercially available 2450 MHz, Sharp Model R-305KS (Sharp Electronics, Mahwah, N.J.) microwave oven with revolving glass carousel, 1100 W (manufacturer rated); 750 W/ft3 experimentally measured; 2 min total exposure at a power setting of 10 (maximum power). FFR placed outer-side down on top of two side-by-side pipette tip boxes, centered, (each box 11.7 cm × 8.0 cm × 5.0 cm) with 50 ml room temperature tap water (∼20ºC). Following treatment, FFRs dried overnight on a laboratory benchtop. | - Tolerability. - Usability (specifically, odor, comfort, or donning ease). | (1) 3M 8000, (2) 3M 8210, (3) Moldex 2200, (4) 3M 1860, (5) 3M 1870, and (6) Kimberly Clark PFR95–270. | None. | Test technicians did not report any unusual or strong odor from FFRs following the decontamination method. While five of the six FFR models included in this study contained metallic nosepieces (only the Moldex 2200 did not), no sparking occurred from microwaving during the MGS processing. There was no melting of an FFR or any of its components. The 3M Model 1870 (3M, St. Paul, Minn.) samples experienced a slight separation of the inner foam nose cushion (some to a lesser or greater degree)from the FFR body following microwave-generated steam (MGS) treatment. |
| Bergman et al 2011 [52] | 2 min exposure at power setting 10 (maximum power). Commercially available 2450 MHz, Sharp Model R-305KS (Sharp Electronics, Mahwah, NJ) microwave oven with revolving glass carousel, 1100 W (manufacturer rated); 750 W/ft3 experimentally measured. FFR placed outer-side down and centered on top of two side-by-side pipette tip boxes (each box 11.7 cm x 8.0 cm x 5.0 cm). Each box contained 50 ml room temperature tap water (~20 °C). | - respirator fit (odor, comfort, or donning ease) | Three models of surgical N95 respirators (3M 1860, 3M 1870, and Kimberly Clark PFR95-270 | None | Did not cause significant changes in respirator fit. Treatment caused the 3M 1870 samples to experience a slight separation of the inner foam nose cushion from the FFR body; however, multiple treatments were not noticed to cause a more pronounced separation than a single treatment. MGS treatment caused one head strap melting in a Kimberly Clark PFR95 270 sample during the third treatment, and the FFR subsequently could not be donned for the final fit test trial While all three FFR models included in this study had metallic nosepieces, no sparking occurred during the MGS processing. |
| Heimbuch et al 2011 [50] | For microwave-generated steam, two plastic reservoirs (4.5 cm h X 12 cm w X 8 cm l) with perforated tops (192 holes of 6 mm diameter, spaced uniformly over the entire surface) were filled with 50 mL of tap water at 22ºC-25ºC. The reservoirs were placed together, and the H1N1-contaminated FFR was set atop the center of the assembly, with the exterior of the FFR resting on the surface of the reservoir. The reservoir assembly and FFR were loaded into the center of a 1250-watt microwave oven and irradiated at full power for 2 minutes. After treatment, the reservoir was replenished with fresh tap water (22ºC-25ºC C), and the next FFR was processed. | - Inactivation of the H1N1 virus | 6 FFR models (3 particulate, designated P1-P3, and 3 surgical, designatedS1-S3). | H1N1 influenza virus. | Provided an average >4-log reduction of viable H1N1 influenza virus against both the droplet and aerosol challenges for all 6 FFRs. No other FFRs showed noticeable deterioration or deformation, and no arcing in the microwave was observed during treatment. |
| Fisher et al 2011 [49] | Two brands of MSBs used for this study, namely, the Medela Quick CleanTM MICRO-STEAMTM BAGS (Medela, McHenry, IL) and the Munchkin® Steam Guard TM Bags (Munchkin Inc., North Hills, CA). These bags will be denoted as ‘‘MSB X’’ or ‘‘MSB Y’’ for the former and latter. Both steam bag brands have similar design structures, including a zipper-lock seal, a steam exhaust port, an internal pleat, and a volume of approximately 2.2 L. The manufacturer’s instructions for use with baby feeding accessories were applied to the steam treatment of FFRs. The instructions were the same for each steam bag brand. Individual FFRs were placed inside separate bags filled with 60 ml of tap water. The bags were sealed using the bag’s integrated zipper-lock seal and placed in a commercially available Sharp Model R-305KS (2450 MHz, 1100 W) microwave oven (Sharp Electronics, Mahwah, NJ, USA). The FFRs in the sealed steam bags were irradiated on high power for 90 s; the prescribed time for a microwave with a rating of 1100 W. | - Filtration performance. - Water absorption. - Decontamination efficacy. - Ease of use. - Logistic benefits. | 3M 1860, 3M 8210, Cardinal Health N95, 3M 1870, Kimberly-Clark PFR95 and Moldex 2200 | MS2 Bacteriophage | The six FFR models (one sample per model) surpassed the filtration efficiency requirements of 95%. The absorption values for models 3M 1860, 3M 8210, and the Cardinal Health N95 were roughly an order of magnitude higher than the values for 3M 1870, Kimberly-ClarkPFR95, and Moldex 2200. The models 3M 1860, 3M 8210, and the Cardinal Health N95 remained wet after the 60 min drying period and were eliminated from further testing. The FFR models, 3M 1870, Kimberly-Clark PFR95, and Moldex2200 passed filtration efficiency testing after three decontamination cycles using both steam bag brands. For the MSB X bags, the filtration efficiencies of the experimental models were statistically similar to the controls for both the 3M 1870(p = 0.19) and the Moldex 2200 (p = 0.40), while the treated Kimberly-Clark PFR95 models were statistically different from the controls (p = 0.01). MSB Y bags produced statistically similar results for the control and treated samples for each model; 3M1870 (p = 0.19) Moldex 2200 (p = 0.40) and Kimberly-ClarkPFR95 (p = 0.42). The drying results of the FFRs were similar for 30 min compared to the 60 min drying time. The average decontamination efficacy resulting from MSB X bags' use was greater than 99.9% (3 logs). The average decontamination efficacy for the Moldex model was greater than 99.99% or 4 logs. The MS2 challenge concentration for the Moldex models was more than 2 logs higher than the Kimberly Clark (7.1) or 3M 1860 (7.6). MSB Y bags achieved a 99.9% reduction of MS2 for two FFR models, while the third model's results measured greater than or equal to 99.86%. |
| Lore et al 2012 [53] | Microwave-generated steam (MGS): A 1250-W (2450MHz) commercially available microwave oven (Panasonic Corp., Secaucus, NJ, USA) with a rotating glass plate was used to irradiate a single respirator per treatment. Samples were placed above a plastic box filled with 50 ml of room temperature tap water. The top of the box was perforated with holes (7 mm diameter) evenly distributed over the entire surface to allow MGS to vent through the respirator. The virus-contaminated respirator was placed with the convex surface pointed toward the steam source and the FFR was then irradiated for 2 min at full power. | - Virucidal capability. - Post-decontamination filter performance | 3M models 1860s and 1870 | Influenza A/H5N1 (VNH5N1) | The microwave-generated steam satisfactorily decontaminated the 3M1860s and 1870 FFRs as measured by a virus culture method. Within the experiment's constraints, the three methods were all completely effective for the decontamination of FFRs as assessed by a culture method. |
| Pascoe et al 2020 [62] | Industrial-grade 2.45-GHz microwave oven (NE-1853; Panasonic), with adjustable power settings up to a maximum output of 1800 W, was used as a microwave irradiation source. A microwave steam ‘sterilizer’ was used to provide moist heat. Before use, the sterilizer base was filled with either 100 or 200 mL of 20°C deionized water. The power could be varied via changing the duty cycle, and 1800-W (100% duty cycle) and 900-W (50% duty cycle) exposures were assessed in this study. The final decontamination procedure used to prepare masks and respirators for downstream testing were prepared using 1800 W power for 90 s alongside 200 mL of water in the reservoir. For decontamination, inoculated membranes were carefully placed on the inner and outer sides of a folded N95 respirator (i.e., on the top and within the area within the fold). Samples were irradiated for 60, 90, and 120 s using either 900- or 1800-W power settings. To verify that PPE reached a minimum target temperature of 70°C, irreversible heat indicator stickers were placed within the steam sterilizers and inspected the following irradiation. | - Effect of essential oils on filtration efficiency. - Bacterial filtration efficiency via aerosol nebulization. - Assessment of mask NaCl filtration efficiency. | FFP2/N95-type respirators (Kimberly-Clark; Fluidshield) | *Staphylococcus aureus* | Microwave (200 mL in ‘sterilizer’): 900W for 90s Log10 reduction (S. aureus) = 0.30, SD 0.28 1800W for 90s Log10 reduction (S. aureus) = 6.85, SD 0.51  Microwave (100 mL in ‘sterilizer’): 900W for 120s Log10 reduction (S. aureus) = 3.96, SD 1.10 1800W for 60s Log10 reduction (S. aureus) = 6.14, SD 0.35  Bacterial filtration efficiency (%): ≥99.9%: MGS reprocessed once - Log10 cfu recovery from sterile discs ≤0.30 (SD) ±0.00 ≥99.9%: MGS reprocessed three times Log10 cfu recovery from sterile discs ≤0.30 (SD)  There were no detectable changes for aerosol filtration efficiency. Kimberly–Clark N95 respirators have a flat metal clip across the nose bridge and were compatible with microwave reprocessing, as was the respirator, which contained no metal components. The Honeywell FFP3 respirator was incompatible as the nose clip arced during the microwave procedure. This led to the heating of the material, which created holes along the bridge of the nose. A generic unbranded respirator with a flat metal nose clip did not cause arcing. There was a loss of adhesion between the clip and respirator following a single MGS-reprocessing cycle, which may be due to the adhesive degradation. Elastic fasteners did not appear to be negatively impacted by MGS-reprocessing. |
| Zulauf et al 2020 [72] | (1) A 10-cm-diameter mug was filled with 60 ml of distilled water and covered with mesh from a produce bag, secured with a rubber band. Triplicate N95 1-cm2 coupons were placed on top of the mesh. The mug was then placed in the microwave either in a sealed, ventilated Ziploc bag or directly into the microwave. After a 1-min microwave treatment, with or without Ziploc bag enclosure, phage was extracted from N95 coupons and quantified by plaque assay. Triplicate untreated N95 coupons were included as controls in all assays.  (2) To identify the optimal length of microwave time required for MS2 phage decontamination, they performed a dose-response test using 1-min increments where they examined the decontamination of 107 PFU of MS2 on 1-cm2 N95 coupons placed over an open mug.  (3) To accurately assess the ability of this method to decontaminate all areas of an N95 respirator, 107 PFU of MS2 was spotted on 10 premarked sections of a whole N95 respirator. After a 3-min treatment in a 1,100-W microwave, demarcated pretreated segments measuring 1 cm2 were excised from the respirator, and MS2 phage was then extracted and quantified by plaque assay.  (4) Ultimately, they selected a generic glass container-sized at 17 × 17 × 7.5 cm (length [L] × width [W] × height [H]) that had an opening large enough to expose the entire N95 respirator to the vertical column of the generated steam. They secured mesh from a produce bag over the top of the container with a rubber band and added 60 ml of distilled water to the basin. They repeated a sterilization time course against 1-cm2 N95 respirator coupons in 1-min increments in a 1,100-W microwave. | - Decontamination | 3M 1860 N95 | *Escherichia coli* MS2 bacteriophage | (1) After 1 min of microwave steam decontamination, there was no significant difference in MS2 phage reduction between the two methods. Both methods resulted in a greater than 4-log10 reduction in MS2 titer after only 1 min of microwave treatment. However, the Ziploc bag melted under this treatment and posed the risk of steam burns during retrieval of the N95 respirator.  (2) Following 3 min of microwave steam treatment, there was no detectable MS2 phage remaining on the coupons.  (3) Following 3 min of treatment, they observed a greater than the 4-log10 reduction in PFU on all N95 respirator segments, except the elastic straps, which showed only a 1- to a 3-log10 reduction in PFU. Due to the mug's limited diameter, the elastic straps draped over the edges and presumably were minimally exposed to microwave-generated steam. Consequently, they hypothesized that direct exposure to steam is essential for effective decontamination and sought to identify a commercial container of sufficient diameter to treat an entire respirator.  (4) After 2 min of microwave steam treatment, they could not detect residual viable phage on the coupons. This represents a 1-min reduction in sterilization time compared to the ceramic mug decontamination assay, indicating that the glass container is a more efficient decontamination system. |
| **9. Method: Sodium hypochlorite** | | | | | |
| Viscusi et al 2007 [29] | Bleach; Fisher 5.25% Sodium Hypochlorite (NaOCl) with 0.20% Sodium Hydroxide (NaOH). Based on the treatment vessel's capacity, either 4 or 8 respirator samples were submerged into a dishpan or 4L beaker containing 3-5 liters of treatment solution. When needed, a second dishpan was used to keep respirators submerged for the time interval the 10 to 30 minutes. 1st Test Condition Less aggressive: dunk 30 minutes, 0.525%. 2nd Test Condition More aggressive: dunk 30 minutes 5.25%. Respirators were removed, hung on a pegboard, and air-dried for 72 hours before filter penetration testing. TSI Model 8130 Automated Filter Tester (AFT) (TSI) was used to measure filter penetration (Pen). With room temperature with a continuous airflow of 85 L/minute. Particle penetration was determined using a cubic Plexiglas box placed between the filter chucks. | - Filtration performance | Two classes of FFRs were used; the N95 class and the P100 class. A single respirator model from the same manufacturer was selected for both FFR classes tested. | None. | The aluminum nosebands were tarnished by both treatments (0.525% and 5.25% bleach) after a 30-minute submersion. For the 0.525% treatment, average penetration was not significantly changed for N95 samples, but average penetration significantly increased for P100 not beyond the NIOSH certification criteria of 0.03%. Treatment with 5.25% bleach resulted in the stiffening of filter media and elastic straps for both respirator models. N95 average penetration was increased from baseline but was still less than the 5% maximum specified by NIOSH certification. P100 penetration results were more variable, although the average penetration was not significantly increased. |
| Vo et al 2009 [48] | Sodium hypochlorite (NaOCl) solutions (stock solution, 6% NaOCl). In the chemical decontamination experiments, all sodium hypochlorite working solutions (0.005, 0.01, 0.05, 0.1, 0.25, 0.5, and 0.75%) was freshly prepared. Each FFR loaded with MS2 was submerged in 1 liter of a sodium hypochlorite solution or puriﬁed water. Treatment with water (with no NaOCl) was used as a baseline treatment to determine losses due to the handling of FFR samples during the chemical decontamination process. Both sides of a complete FFR were decontaminated by submerging the FFR in a sodium hypochlorite solution. After 10 min of treatment, the respirator was removed from the puriﬁed water or sodium hypochlorite solution and air-dried for 2 min. A toxicity control to determine if there was any interference by residual sodium hypochlorite with the chemical inactivation process under these conditions (10 min of sodium hypochlorite treatment and 2 min of air drying). Each respirator was cut into coupons (2 cm by 2 cm), and each coupon was then placed in 10 ml of 271B in a 50-ml conical tube for extraction. | Effectiveness of procedures for decontamination and decreases in the number of viable viruses recovered from the respirators. | N95 FFR (models N1105) | MS2 coliphage (ATCC 15597-B1) | Sodium hypochlorite doses of 2.75 to 5.50 mg/liter with a 10-min decontamination period resulted in approximately 3- to 4-log reductions in the level of MS2 coliphage. When higher sodium hypochlorite doses (>8.25 mg/liter) were used with the same contact time used for the dilute solutions containing 2.75 to 5.50 mg/liter, all MS2 was inactivated. |
| Viscusi et al 2009 [30] | Thirty minutes submersion in 0.6% (one part bleach to nine parts of deionized water) aqueous sodium hypochlorite (original concentration = 6% available as Cl2). Manufacturing specification: 6.00–0.06% (w/w) available chlorine; Cat no. 7495.7-1, CAS no. 7732-18-5 (Ricca Chemical Company). After treatment, FFRs were hung on a laboratory pegboard and allowed to air-dry overnight with a fan. | - Changes in physical appearance. - Odor changes. - Laboratory performance (filter aerosol penetration and filter airflow resistance). | Random sampling from those N95 FFR models presented in the US Strategic National Stockpile (SNS). | None. | Metallic nosebands were slightly tarnished and visibly not as shiny when compared with their as-received counterparts. SN95-E inner nose comfort cushion was discolored. Following air-drying overnight for 16-hours, all FFRs were dry to the touch, and all still had a characteristic smell of bleach. |
| Fisher et al 2009 [71] | The decontamination efficacy of sodium hypochlorite (Clorox; registration no. 5813-50) at concentrations of 0.0006, 0.006, 0.06, and 0.6% was tested in triplicate. Respirator coupons were used as air-permeable inanimate carriers and exposed to MS2 (approximately 6 logs/coupons) the BARTS methods. After the exposure to MS2, one coupon was placed directly into 10 ml of ATCC medium 271 as a loading level control. One coupon was submerged in water (dipping control), and one coupon was placed in sodium hypochlorite at each of the different concentrations in a 50-ml conical tube. After 10 min of treatment, the coupons were carefully removed from the sodium hypochlorite solutions and air-dried for 2 min. Each coupon was then placed in 10 ml of ATCC medium 271, performing a plaque assay. The process was repeated for triplicate. | - Virucidal efficacy | NIOSH-certified N95 | Coliphage MS2 (ATCC 15597-B1) | Bleach concentrations of 0.006%, 0.06%, and of 0.6% for low-protective-factor (LPF) medium and 0.6% for high-protective-factor (HPF) medium resulted in log reductions which reached the detection limits. |
| Bergman et al 2010 [32] | Multiple (3-Cycle) of: 30-min submersion in 0.6% (one part bleach to nine parts of deionized water) solution of sodium hypochlorite (original concentration = 6% available as Cl2). Manufacturing specification: 6.00 ± 0.06% w/w) available chlorine; Cat No. 7495.7-1, CAS No. 7732-18-5 (Ricca Chemical Company). Following each exposure, FFRs were hung on a laboratory pegboard and dried for a minimum of 16 hours with a fan's aid before repeating the treatment or performing the laboratory aerosol filtration test. | - Filtration performance. - Physical integrity. - Filter airflow resistance. | Six models:  -Three N95 FFR models - N95-A, N95-B, and N95-C  -Three surgical N95 respirator models - SN95-D, SN95-E and SN95-F | None. | Bleach exposure caused various effects: for all FFR models, metallic nosebands were slightly tarnished and visibly not as shiny as their as-received counterparts. Those models with staples (N95-B, N95-C, SN95-E, and SN95-F staples were oxidized to varying degrees. Three models (N95-A, SN95-E, and SN95-F) had discolored (yellowed) inner nose pads. The nose pad of model SN95-E samples dissolved (only 50% remained). Discoloring of other areas of the FFR was observed in models SN95-F (bleeding of printed ink lettering), SN95-E (material adjacent to nose pad became yellowed), and SN95-D (the area adjacent to nose clip discolored). Following air-drying between exposure cycles (at least 16 hr), all FFRs exposed to bleach were dry to the touch, and all still had a characteristic bleach odor.  The 3X treated samples of model SN95-D showed a much larger mean penetration (4.01%) compared to 1X bleach-treated for the same model. The 3X and 1X bleach treatment mean % penetration P values were similar for the other five models. |
| Salter et al 2010 [31] | Sodium hypochlorite (0.6%) | -The amount of residual chemicals created or deposited. | NIOSH, FDA-approved N95, Surgical FFR, NIOSH-approved N95 and Particulate FFR | None. | All FFR models treated with 0.6% hypochlorite retained similar amounts of an oxidant except for S3, on which none was detected. |
| Lin et al 2017 [69] | 10 min submersion in 0.5% sodium hypochlorite solution (original concentration = available as Cl2). Manufacturing specification: 0.5% (w/w) available chlorine. Following each exposure, masks were placed in a laboratory chemical hood and allowed to air-dry overnight before performing the laboratory aerosol filtration test. | - Penetration of particles through mask. - Pressure drop. - Filter quality. | Not specified N95. | None. | The submergence in bleach changed significantly the penetration through the N95 masks, decreased the quality of the filter with the increase of the penetration, and increase the pressure drop. Probably because it eliminated the electrostatic charges in the filters.  Numerical parameters of the degradation were not provided in the manuscript. |
| Lin et al 2018 [57] | A 0.4 ml volume of bleach with various concentrations(5.4% (w/w) as Cl2: original; 2.7%: one part bleach to one part of deionized water; 0.54%: one part bleach to nine parts of deionized water) was added to the center of the surface of the N95 FFR using a pipette. The FFR was then dried in a petri dish in a BSC for 10 min. | - Relative survival of Bacillus subtilis spores | N95 FFR (8210,3M, St. Paul, MN) | *Bacillus subtilis Spores* | In the bleach decontamination test, no colony was recovered after 5.4%, 2.7% or 0.54% NaOCl was used, constituting no dilution, two-fold, and ten-fold dilution, respectively. This study found that NaOCl, even when diluted ten-fold from standard bleach, had a strong decontamination effect, with a 100% bactericidal effect. |
| Liao et al 2020 [58] | Samples were sprayed with approximately 0.3-0.5 mL of household chlorine-based disinfectant (~2% NaClO). Samples were left to air dry and off-gas completely, hanging. Samples were tested. | - Filtration efficiency.  - Pressure drop. | 3M 8210 (NIOSH N95), 4C Air, Inc. (GB2626 KN95), ESound (GB2626 KN95) and Onnuriplan (KFDA KF94). | None. | It can clearly note that chlorine-based solution drastically degraded the filtration efficiency to unacceptable levels from the first disinfection while the pressure drop remained comparable. |
| Peltier et al 2020 [42] | Respirators were donned on a mannequin that was covered in a layer of soft closed-cell foam. The mannequin was installed in a 0.1-m3 exposure chamber and flooded with polydispersed combustion aerosol (10% diluted bleach). Protocol 1- six Respirators Tested, Cycle Time 10 minutes; Repeated Decontamination Treatments (1x, 5x). | - Filtration efficiency - Respiratory performance, | 3M 1860 or 1860S models (3M, StPaul, MN). | None. | The performance of the N95 respirator that was immersed in a 0.5% bleach solution was degraded. |
| **10. Method: Autoclave** | | | | | |
| Viscusi et al 2007 [29] | Autoclave 121°C (15 psi) All samples were sealed in a standard poly/paper autoclave bag and treated in a Market Forge Automatic Sterilmatic Steam Pressure Sterilizer (Everett, MA) for the time the 15 or 30 minutes. The respirators were then air-dried for 72 hours before filter testing. TSI Model 8130 Automated Filter Tester (AFT) (TSI, Inc., St. Paul, MN) was used to measure filter penetration (Pen). With room temperature with a continuous airflow of 85 L/minute. Particle penetration was determined using a cubic Plexiglas box (20 x 20 x10 cm3) placed between the filter chucks (sample holding mechanism on the ATF). | - Filtration performance | Two classes of FFRs were used; the N95 class and the P100 class. A single respirator model from the same manufacturer was selected for both FFR classes tested. | None. | For both treatment conditions (30 and 15 minutes), the N95 FFRs were deformed. Both treatment conditions markedly increased the average shrunken, stiff, and mottled. No remarkable visual changes were observed in the P100 respirators, though the respirator media itself felt softer. Penetration for both classes of respirators. This observation is not surprising given the general push towards low-temperature sterilization methods for sensitive materials and equipment. Temperature greater than 80° C will likely affect the performance of a filter. The maximum filter operating temperature for non-woven polypropylene is 90-100° C |
| Lin et al 2017 [69] | Set the temperature at 121ºC with 1.06 kg cm−2 for 15 minutes. | - Penetration of particles through mask. - Pressure drop. - Filter quality. | Not specified N95. | None. | Wet heat in the autoclave had little effect on that of the N95 mask. |
| Lin et al 2018 [57] | The N95 FFR was heated for 15 min at 121°C and 103 kPa. | - Relative survival of Bacillus subtilis spores | N95 FFR (8210,3M, St. Paul, MN) | *Bacillus subtilis Spores* | Effectively sterilized almost 100% of the bacteria. |
| Carillo et al 2020 [73] | Immediate-use steam sterilization (IUSS), using a Steris Amsco Evolution HC1500 PreVac Steam Sterilizer autoclave (Steris, Mentor, OH) was performed on N95 masks. Masks were packed in paper-plastic sterilization peel pouches for IUSS (Medical Action Industries 8” role, no. 422R). Masks were photographed and fit tested before IUSS, and this testing protocol was repeated after the IUSS cycle. Quantitative fit tests were performed using the gold standard TSI PortaCount Respirator Fit Tester (TSI, Shoreview, MN). A fit test was performed before the IUSS cycle as a control. Fit tests were performed again after 3 IUSS cycles. | - Respirator fit | 3M 1870 and M3 1870+ masks (3M, Saint Paul, MN) | None. | According to the authors, in all cases, masks retained their structural integrity and efficacy. However, no results are provided regarding the filtering capacity. Only the fittest was performed. |
| Czubryt et al 2020 [74] | NRMs wore by volunteer Animal Care Centre laboratory workers for 2–8 h were collected and autoclaved at 121°C for 30 min plus 15 min drying time (total cycle length 48 min) in a Steris Amsco 400 Series Pre-vacuum Steam Sterilizer Model 20 (Steris Corp., Mentor, OH, USA). Biological indicators (Attest 1292) were included in each autoclave cycle to confirm sterilization. Quantitative fit testing was performed using a PortaCount PRO+ 8038 to evaluate respirator facial seal during seven work-simulating exercises (the 60s each): normal breathing, deep breathing, side-to-side head-turning, head nodding up and down, talking out loud, bending over, and the second round of normal breathing. Fit factor scores were assigned for each exercise according to protocol CSA Z94.4–2011; respirator masks yielding an average fit factor <100 failed fit testing and were discarded. | - Respirator fit | AO Safety 1054S Pleats Plus | No specific agent. | This investigation revealed that, whereas all NRMs passed fit testing after a single round of sterilization by autoclave, masks started failing after the second round of wear and sterilization. Although this number was relatively small – two masks out of 14 failed fit testing (14% failure rate) – this failure necessitated that the entire lot be discarded. |
| Harskamp et al 2020 [75] | The masks were decontaminated for multiple cycles using a cylindrical chamber tabletop autoclave (Kronos S18). The sterilization program involved a 34 min cycle. After decontamination, the respirators were checked for visual deformities. Subsequently, the respirators were put on to evaluate whether breathing felt normal, followed by a user seal check's performance. Two independent researchers from the Delft University of Technology tested the masks at the testing laboratory using a dry particle penetration test set-up. The equipment involved a SOLAIR 3100 particle counter (Lighthouse Worldwide Solutions). | - Signs of deformity of the respirator, which was performed by visual inspection. - The percentage of filtered particles with a diameter of 0.3 µm. | The face masks were FFP-2 respirators (3M Aura 1862+, Maco Pharma ZZM002), FFP-2 respirators with exhalation valve (3M Aura 9322+and San Huei 2920V)  03111 | None. | After the decontamination process, all FFP-2 respirators retained their shape and were without visible damage. When fitting, all masks' elastic bands still functioned normally, with no difference from non-decontaminated masks in terms of breathing resistance. The seal checks also did not reveal significant air leakage suggesting poor fit. Of the tested FFP-2 respirators, they found that the 3M Aura 1862+ remained close to its original filtering capacity after one-time, two-time, and three-time decontamination (0.3 µm: 99.3%±0.3% vs. 97.0±1.3, 94.2±1.3% or 94.4±1.6, respectively, p<0.001). The 3M Aura 9322+ (with valve) had a filter capacity of 96.8%±0.2% without decontamination vs 91.0%±1.4% and 77.5%±2.1% after one-time or two-time decontamination (p<0.001). The Maco Pharma ZZM002 FFP-2 mask did not have a reference mask, but after one-time and two-time decontamination, the filter capacities were 89.3%±3.9% and 86.6%±2.6%, respectively. The San Huei 2920V respirator had 95.5%±0.7% at baseline vs 92.3%±1.7% vs 90.0±0.7 after one-time and two-time decontamination (p<0.001). The breathing resistance test tested six FFP-2 respirators (3M Aura 1862+): two were used once and reprocessed; two were used twice and reprocessed after each use, and two were used three times and reprocessed after each use. The average pressure did not increase with the number of reuses (35.6%±0.3%, 35.4%±0.0%, 36.7%±0.3%, respectively). |
| Grinshpun et al 2020 [70] | Sterilization in an autoclave Tuttnauer Model 5596, under 250 F at 15 psi for 30 min, fast exhaust following by drying for 30 min. This was performed once and consecutively five times. To simulate contamination of facepieces between donning, they were soiled with protein egg whites derived from commercial eggs were dialyzed against de-ionized distilled (Milli-Q) water. Protein solution containing 1 mg of egg whites in 3 mL of water was sprayed on masks/respirators before their sterilization in an autoclave. When multiple sterilizations were applied, facepieces were soiled before each autoclave treatment to mimic the device usage in air environments contaminated with a protein associated with the emission of pathogenic virions by infected persons. They choose to perform five + sterilization cycles to simulate daily re-use of the filtering facepiece over a period of 5 days. Conditions of the tested respirator: new, autoclaved once, autoclaved five times, soiled and autoclaved once, soiled and autoclaved five times. | Efficiency, pressure drop, damage | 3M Corp Model 1870. | None. | Even the most penetrating particles were collected at 99.1% at 30 L/min and 97.1% at 85 L/min for single and multiple sterilizations and soiled and non-soiled filters. However, even with this reduction, the N95 filter offered a 97.5% efficiency or higher at 30 L/min. At the higher flow rate (85 L/min), the filter collected more than 95% of particles of all tested sizes under all tested scenarios with one exception: (soiled + autoclaved) × 5, when Ec decreased to 93.6−94.8% for particles of 0.037−0.52 μm, just a little below the 95% benchmark. The Ec value was still above 95% for larger particles. It is noted that the above particle size range includes the single coronavirus as well as particle-carriers that are up to five times bigger in size. The initial air pressure drop through the N95 filter was moderate (3.1 mm wg at 30 L/min and 12.7 mm wg at 85 L/min). For the N95 FFR, the post-treatment pressure drop decreased by 13–35%, depending on the flow rate and decontamination method applied. |
| **11. Method: Electric rice cooker** | | | | | |
| Lin et al 2017 [69] | Place the test masks in a traditional electric rice cooker using dry heat for 3 minutes (149~164 C, without adding water). | - Penetration of particles through mask. - Pressure drop. - Filter quality. | Not specified N95. | None. | The experimental results indicate that decontamination by dry heat in a rice cooker had little effect on particles' penetration through the N95 mask. |
| Lin et al 2018 [57] | The N95 FFR was placed in an electric rice cooker for dry heating for 3 minutes (149-164°C, without added water). | - Relative survival of Bacillus subtilis spores | N95 FFR (8210,3M, St. Paul, MN) | *Bacillus subtilis Spores* | Effectively sterilized almost 100% of the bacteria. |
| Li et al 2020 [64] | The 10-mL aliquots containing 106 colony-forming units or plaque-forming of the test organisms suspended in 8% simulated mucus were inoculated onto 1-cm 2 areas on both the outer or inner surfaces of the respirators or face masks. The inoculated masks or respirators were subjected to a cycle of treatment in a steamer (Aroma; San Diego, CA) lasting approximately 13-15 minutes, including 8-10 minutes of heating and 5 minutes of steam. After treatment, the inoculated sections of the face masks and N95 respirators were vortexed for 1 minute in 1 mL of phosphate-buffered saline with 0.02% Tween and plated quantify organisms. The tests were performed in triplicate. A reduction of 3-log10 or greater in recovery of organisms inoculated was considered effective for decontamination. | - Decontamination | 3M 1860 N95 respirators (3M; Saint Paul, MN) | *methicillin-resistant Staphylococcus aureus single-stranded RNA virus bacteriophage MS2* | The treatment resulted in a greater than 5 log10 reduction in bacteriophage MS2 and methicillin-resistant S aureus. No visible changes were observed in any of the masks or respirators after 5 cycles of decontamination. However, it did not examine the effect of treatment on respirator or face mask performance. Further studies are needed to evaluate steam treatment for N95. |
| **12. Method: Cleaning Wipes** | | | | | |
| Heimbuch et al 2014 [76] | Wipe products selected were 504/07065 Respirator Cleaning Wipes (3M Company, St Paul, MN), which contain benzalkonium chloride (BAC); Hype-Wipes (Current Technologies, Inc, Crawfordsville, IN), which contain 0.9% hypochlorite (OCL); and Pampers wipes (Proctor & Gamble, Cincinnati, OH), which contain no active antimicrobial ingredients. BAC and other quaternary ammonium disinfectants commonly appear in wipe products. The OCL wipe was included to measure the ability of a limited application (wiping vs. immersion) to remove contaminants and minimize incompatibilities with FFRs. Alcohol- and soap-based wipe products were avoided because they are known to decrease FFR performance. | - Physical removal of deposited contaminants - Effectiveness of disinfection | 3M-1860S (FFR A); 3M-1870 (FFR A); Kimberly-Clark-46727-PFR (FFR A) | *Staphylococcus aureus* | Reduction in viable S aureus varied among wipe—FFR component pairs. The mean loading concentration of S aureus on FFR samples was 6.72 × 105 CFU/cm2. The inert wipes captured 81.56%–96.53% of Staphylococcus aureus from the base fabrics of all FFR models tested. REs were low for the exterior surface of perforated edge strips from FFR C (59.37%) and FFR B’s nose pad (69.28%). OCL wipes reduced viability below the detection limit (>5-log attenuation) for 7 of 10 samples among the 3 FFR models. Two remaining samples (interior fabrics of FFRs B and C) lost >4 logs in viability, the last sample (nose pad of FFR B) showing the smallest decrease (98.98%) of the sample set. BAC wipes produced 2 samples below the detection limit (the interior surface of perforated edge strip from FFR C, the interior fabric of FFR B); 5 other samples showed 3–5 log reductions in viability. Attenuation on FFR B’s nose pad again was the least (68.92%) of the sample set. Mean particle penetration of each thrice-cleaned FFR model was <5%, NIOSH’s N95 certification criterion. For all 3 FFR models tested, BAC wipes caused more penetration than the other wipes; for FFRs A and B, this difference was significant (P < 0.05). Of the models tested, FFR C showed the greatest penetration—1 replicate exceeded the 5% threshold (5.6%) after cleaning with a BAC wipe—and the differences were not significant. |
| **13. Method: bar soap and water** | | | | | |
| Viscusi et al 2007 [29] | Ivory bar soap, 1g/L, shaved from the bar and diluted in tap water. Based on the treatment vessel's capacity, either 4 or 8 respirator samples were submerged into a dishpan or 4L beaker containing 3-5 liters of treatment solution. When needed, a second dishpan was used to keep respirators submerged for the time interval the 2 and 20 minutes. 1st Test Condition Less aggressive: 2 minutes. 2nd Test Condition More aggressive: 20 minutes. Respirators were removed, hung on a pegboard, and air-dried for 72 hours before filter penetration testing. TSI Model 8130 Automated Filter Tester (AFT) (TSI, Inc., St. Paul, MN) was used to measure filter penetration. With room temperature with a continuous airflow of 85 L/minute. Particle penetration was determined using a cubic Plexiglas box placed between the filter chucks. | - Filtration performance | Two classes of FFRs were used; the N95 class and the P100 class. A single respirator model from the same manufacturer was selected for both FFR classes tested. | None. | No visible changes were observed. Average penetration was markedly increased for N95 respirators at both time intervals, for the P100 samples, the 2 min. Treatment resulted in a slight increase in penetration while the 20 min. Treatment significantly increased average penetration. Dipping in the water had essentially no effect. It would follow that the soap was likely to be responsible for the increase in filter penetration. It is possible that the soap removed the charge on the fibers similar to the effect observed with IPA exposure. |
| **14. Method: Multi-Purpose High-Level Disinfection Cabinet (Altapure Mequon WI)** | | | | | |
| Cadnum et al 2020 [59] | Tested 1, 2, and 3 cycles of treatment with the cabinet to determine if longer treatment time would increase efficacy. Because multiple cycles increased efficacy, also tested a single extended cycle with a 15-minute dwell time and 31-minute total cycle time. After completing the treatment, log10 CFU or PFU reductions were calculated by comparing recovery from exposed respirators to untreated control respirators. The tests were performed in triplicate. For the testing with 3 cycles, additional testing was completed with the 2 other respirator types. The suspension containing 106 PFU of MS2 and 106 CFU of MRSA was sprayed onto the entire inner and outer surface of the Moldex 1517 respirator. Finally, they tested the efficacy of a single 21-minute cycle. | - To determine the efficacy against organisms on an N95 respirator. | Moldex 1517 N95 | Methicillin-resistant Staphylococcus aureus (MRSA) Bacteriophage MS2 | A high-level disinfection cabinet that generates aerosolized peracetic acid and hydrogen peroxide was more effective and met the criteria for disinfection with an extended cycle de 31 minutes. With 1, 2, and 3 treatment cycles of 21 minutes and with an extended 31-minute cycle, reductions of >2.1, >3.6, and >6 log10 PFU or CFU were achieved for all the test sites. Additional testing with the other 2 respirator types demonstrated similar results. The 3-cycle treatment effectively achieved>6-log10 on the Moldex 1517 respirator when the suspension containing 106 PFU of MS2 and 106 PFU or CFU reductions CFU of MRSA was sprayed onto the entire inner and outer surface. No visible changes were observed in any of the respirators after 3 or more cycles. |
| **15. Method: Chlorine dioxide (ClO2)** | | | | | |
| Cai and Floyd 2020 [36] | A stable salt aerosol was generated using a 3-jet Collison nebulizer (CH Technologies) and 2% sodium chloride solution by the National Institute of Occupational Safety and Health procedure No. TEB-APR-STP-0059. A scanning mobility particle sizer (model 3936; TSI) was used to measure the particle number concentration from 16.8 nm to 514 nm. All masks were preconditioned in an incubator at 38 °C and 100% relative humidity for 12 hours. For each mask, 5 samples were tested with 4 upstream measurements and 4 downstream measurements. Acceptable pressure drop was defined as less than 35 mm or 1.38-inch water for inhalation. | - Filtration performance | N95s (model 1860; 3M) N95s (Civilian Antivirus; Qingdao Sophti Health Technology) | None. | The mean (SD) filtration efficiencies of untreated masks were 97.3% (0.4%) for N95s and 96.7% (1.0%) for KN95s. After ClO2 sterilization, the filtration efficiencies were 95.1% (1.6%) for N95s and 76.2% (2.7%) for KN95s. |
