# Supplementary material for: Mask decontamination methods (model N95) for respiratory protection: a rapid review
Source: Syst Rev. 2021 Aug 7;10:219. doi: 10.1186/s13643-021-01742-1 (PMC8349237; doi:10.1186/s13643-021-01742-1)
Supplement: Supplementary file 5 — Additional file 5: Table S4. Results assessing filter aerosol penetration, airflow resistance and filtration efficiency. [file 13643_2021_1742_MOESM5_ESM.docx]

**Supplementary Material 5**

**Table 4. Results assessing filter aerosol penetration, airflow resistance and** **filtration efficiency. The experimental group refers to the different methods included. The control group refers to masks not subjected to any disinfection process. Thus, the closer to the control value, the more advantageous the disinfection method.**

| **1. Outcome: Filter aerosol penetration** | **Experimental** | | | **Control** | | | **Additional information** |
| --- | --- | --- | --- | --- | --- | --- | --- |
| **Study or subgroup** | **Mean** | **SD** | **Total** | **Mean** | **SD** | **Total** |  |
| **1.1 Liquid Hydrogen Peroxide** | | | | | | | |
| Bergman et al 2010 [32] | 1.33 | 0.4617 | 18 | 1.16 | 0.245 | 18 | 3%, 30 min, 03 cycles |
| Viscusi et al 2007 [29] | 0.75 | 0.242 | 4 | 0.7 | 0.267 | 10 | 3%, 30 min, 01 cycle |
| Viscusi et al 2007[29] | 0.71 | 0.148 | 4 | 0.7 | 0.267 | 10 | 6%, 30 min, 01 cycle |
| **1.2 Hydrogen Peroxide Vapor** | | | | | | | |
| Bergman et al 2010 [32] | 0.97 | 0.17 | 36 | 1.16 | 0.245 | 18 | Clarus® R, 03 cycles |
| Bergman et al 2010 [32] | 5.16 | 5.3133 | 36 | 1.16 | 2.45 | 18 | STERRAD® 100S, 03 cycles |
| Viscusi et al 2007 [29] | 0.75 | 0.166 | 4 | 0.7 | 0.267 | 10 | STERRAD® 100S, 01 cycle |
| Viscusi et al 2007 [29] | 0.5 | 0.213 | 4 | 0.7 | 0.267 | 10 | STERRAD® NX, 01 cycle |
| Viscusi et al 2009 [30] | 0.7 | 0.4133 | 18 | 0.87 | 0.5083 | 18 | STERRAD® 100S, 01 cycle |
| **1.3 Ultraviolet Germicidal Irradiation** | | | | | | | |
| Bergman et al 2010[32] | 0.92 | 0.2533 | 18 | 1.16 | 0.245 | 18 | 254 nm, 15 min, 03 cycles |
| Lindsley et al 2015[55] | 2.66 | 1.4435 | 16 | 2.38 | 1.2192 | 16 | 254 nm, 120 Jcmˉ², 01 cycle  All models of N95 FFRs were considered together in the analysis. |
| Lindsley et al 2015[55] | 3.29 | 1.6252 | 16 | 2.72 | 1.5125 | 16 | 254 nm, 240 Jcmˉ², 01 cycle  All models of N95 FFRs were considered together in the analysis. |
| Lindsley et al 2015[55] | 2.98 | 1.7514 | 16 | 2.33 | 1.3365 | 16 | 254 nm, 470 Jcmˉ², 01 cycle  All models of N95 FFRs were considered together in the analysis. |
| Lindsley et al 2015[55] | 2.76 | 1.5111 | 16 | 2.29 | 1.523 | 16 | 254 nm, 950 Jcmˉ², 01 cycle  All models of N95 FFRs were considered together in the analysis. |
| Viscusi et al 2007[29] | 0.57 | 0.152 | 4 | 0.7 | 0.267 | 10 | 254 nm, 30 min, 01 cycle |
| Viscusi et al 2007[29] | 0.79 | 0.082 | 4 | 0.7 | 0.267 | 10 | 254 nm, 480 min, 01 cycle |
| Viscusi et al 2009[30] | 0.93 | 0.4967 | 18 | 0.87 | 0.5083 | 18 | Sterilgard III, 01 cycle |
| **1.4 Ethylene Oxide** | | | | | | | |
| Bergman et al 2010[32] | 1.02 | 0.255 | 36 | 1.16 | 0.245 | 18 | Amsco® Eagle® 3017, 03 cycles |
| Viscusi et al 2007[29] | 1.29 | 0.35 | 4 | 0.7 | 0.267 | 10 | EtO 3M 4XL, 01 cycle |
| Viscusi et al 2007[29] | 1.2 | 0.729 | 4 | 0.7 | 0.267 | 10 | EtO 3M 5XL, 01 cycle |
| Viscusi et al 2009[30] | 0.78 | 0.455 | 18 | 0.87 | 0.5083 | 18 | Steri-Vac 5XL, 01 cycle |
| **1.5 Dry Heat** | | | | | | | |
| Viscusi et al 2007[29] | 0.84 | 0.258 | 4 | 0.7 | 0.267 | 10 | oven, 80ºC, 60 min, 01 cycle |
| **1.6 Moist Heat** | | | | | | | |
| Bergman et al 2010[32] | 0.97 | 0.2433 | 18 | 1.16 | 0.245 | 18 | 60ºC, 80% RH, 30 min, 03 cycles |
| **1.7 Isopropanol solution** | | | | | | | |
| Viscusi et al 2007[29] | 21.6 | 1.337 | 4 | 0.7 | 0.267 | 10 | 1 minute submersion, 01 cycle |
| Viscusi et al 2007[29] | 17.8 | 5.508 | 4 | 0.7 | 0.267 | 10 | 1 second submersion, 01 cycle |
| **1.8 Microwave** | | | | | | | |
| Bergman et al 2010[32] | 1.05 | 0.2567 | 18 | 1.16 | 0.245 | 18 | 2 min exposure, 03 cycles |
| Viscusi et al 2007[29] | 1.13 | 0.662 | 4 | 0.7 | 0.267 | 10 | 2 min exposure, 01 cycle |
| Viscusi et al 2007[29] | 1.77 | 0.617 | 4 | 0.7 | 0.267 | 10 | 4 min exposure, 01 cycle |
| Viscusi et al 2009[30] | 0.73 | 0.48 | 15 | 0.98 | 0.572 | 15 | 2 min exposure, 01 cycle |
| **1.9 Sodium hypochlorite** | | | | | | | |
| Bergman et al 2010[32] | 1.35 | 0.205 | 18 | 1.16 | 0.245 | 18 | 0.6%, 30 min sub, 03 cycles |
| Viscusi et al 2007[29] | 0.68 | 0.124 | 4 | 0.7 | 0.267 | 10 | 0.525% in tap water, 01 cycle |
| Viscusi et al 2007[29] | 3.79 | 3.446 | 4 | 0.7 | 0.267 | 10 | 5.25% in tap water, 01 cycle |
| Viscusi et al 2009[30] | 0.58 | 0.36 | 18 | 0.87 | 0.5083 | 18 | 0.6%, 30 min sub, 01 cycle |
| **1.10 Autoclave** | | | | | | | |
| Viscusi et al 2007[29] | 18.7 | 5.263 | 4 | 0.7 | 0.267 | 10 | 121°C (15 psi), 15 min, 01 cycle |
| Viscusi et al 2007[29] | 34.4 | 9.963 | 4 | 0.7 | 0.267 | 10 | 121°C (15 psi), 30 min, 01 cycle |
| **1.11 Bar soap and water** | | | | | | | |
| Viscusi et al 2007[29] | 38.8 | 1.269 | 4 | 0.7 | 0.267 | 10 | 02 min, 01 cycle |
| Viscusi et al 2007[29] | 34.9 | 4.599 | 4 | 0.7 | 0.267 | 10 | 20 min, 01 cycle |
|  | | | | | | | |
| **2. Filter airflow resistance** | **Experimental** | | | **Control** | | | **Additional information** |
| **Study or subgroup** | **Mean** | **SD** | **Total** | **Mean** | **SD** | **Total** |  |
| **2.1 Liquid Hydrogen Peroxide** | | | | | | |  |
| Bergman et al 2010[32] | 9.78 | 5.833 | 18 | 10.45 | 3.333 | 18 | 6%, 30 min, 03 cycles |
| **2.2 Hydrogen Peroxide Vapor** | | | | | | |  |
| Bergman et al 2010[32] | 10.62 | 4.833 | 36 | 10.45 | 3.333 | 18 | Clarus® R, 03 cycles |
| Bergman et al 2010[32] | 10.58 | 3.833 | 36 | 10.45 | 3.333 | 18 | STERRAD® 100S, 03 cycles |
| Viscusi et al 2009[30] | 7.68 | 0.93 | 18 | 7.52 | 6.567 | 18 | STERRAD® 100S, 01 cycle |
| **2.3 Ultraviolet Germicidal Irradiation** | | | | | | |  |
| Bergman et al 2010 | 11.17 | 6.167 | 18 | 10.45 | 3.333 | 18 | 254 nm, 15 min, 03 cycles |
| Lindsley et al 2015[55] | 11.8 | 3.9553 | 16 | 11.83 | 4.1971 | 16 | 254 nm, 120 Jcmˉ², 01 cycle  All models of N95 FFRs were considered together in the analysis. |
| Lindsley et al 2015[55] | 11.85 | 3.8176 | 16 | 11.87 | 3.8495 | 16 | 254 nm, 240 Jcmˉ², 01 cycle  All models of N95 FFRs were considered together in the analysis. |
| Lindsley et al 2015[55] | 11.66 | 3.7878 | 16 | 11.66 | 3.9936 | 16 | 254 nm, 470 Jcmˉ², 01 cycle  All models of N95 FFRs were considered together in the analysis. |
| Lindsley et al 2015[55] | 12.08 | 3.8758 | 16 | 12.08 | 3.8757 | 16 | 254 nm, 950 Jcmˉ², 01 cycle  All models of N95 FFRs were considered together in the analysis. |
| Viscusi et al 2009[30] | 7.98 | 5.667 | 18 | 7.52 | 6.567 | 18 | Sterilgard III, 01 cycle |
| **2.4 Ethylene Oxide** | | | | | | |  |
| Bergman et al 2010[32] | 11.47 | 3.667 | 36 | 10.45 | 3.333 | 18 | Amsco® Eagle® 3017, 03 cycles |
| Viscusi et al 2009[30] | 7.5 | 0.44 | 18 | 7.52 | 6.567 | 18 | Steri-Vac 5XL, 01 cycle |
| **2.5 Moist Heat** | | | | | | |  |
| Bergman et al 2010[32] | 10.18 | 0.15 | 18 | 10.45 | 3.333 | 18 | 60ºC, 80% RH, 30 min, 03 cycles |
| **2.6 Microwave** | | | | | | |  |
| Bergman et al 2010[32] | 10.63 | 4.667 | 18 | 10.45 | 3.333 | 18 | 2 min exposure, 03 cycles |
| Viscusi et al 2009[30] | 7.44 | 4.648 | 15 | 7.8 | 758 | 15 | 2 min exposure, 01 cycle |
| **2.7 Sodium hypochlorite** | | | | | | |  |
| Bergman et al 2010[32] | 10.02 | 0.5 | 18 | 10.45 | 3.333 | 18 | 0.6%, 30 min submersion, 03 cycles |
| Viscusi et al 2009[30] | 8.0 | 8.567 | 18 | 7.52 | 6.567 | 18 | 0.6%, 30 min submersion, 01 cycle |
|  | | | | | | | |
| **3. Filtration efficiency** | **Experimental** | | | **Control** | | | **Additional information**  **(provided when necessary)** |
| **Study or subgroup** | **Mean** | **SD** | **Total** | **Mean** | **SD** | **Total** |  |
| **3.1 Plasma Vapor Hydrogen Peroxide (PVHP)** | | | | | | |  |
| Cai and Floyd 2020[36] | 96.85 | 1.7 | 10 | 97.0 | 0.7 | 10 | PVHP |
| Cramer et al 2020[43] | 99.67 | 875 | 16 | 98.59 | 142 | 20 | SteraMist-BIT, 01 cycle  The Gerson 2130 model was not considered in the analysis, as there was no reference (control) for this mask model. |
| Cramer et al 2020[43] | 99.33 | 0.03 | 8 | 98.37 | 1.267 | 12 | SteraMist-BIT, 05 cycles  Only the models 3M 1860 and KC/Halyard 46767 were considered in the analysis, as they were the only ones tested in 05 cycles and with reference data (control) |
| Cramer et al 2020[43] | 99.16 | 1.233 | 12 | 98.3 | 0.15 | 16 | SteraMist-BIT, 10 cycles  Only the models 3M 1860, KC/Halyard 46767 and 3M 8210 were considered in the analysis, as they were the only ones tested in 10 cycles and with reference data (control). |
| **3.2 Ultraviolet Germicidal Irradiation** | | | | | | |  |
| Liao et al 2020[58] | 95.0 | 1.59 | 3 | 96.52 | 1.37 | 30 | 254 nm, 8 W, 01 cycle |
| Liao et al 2020[58] | 96.78 | 0.8 | 3 | 96.52 | 1.37 | 30 | 254 nm, 8 W, 10 cycles |
| **3.3 Dry Heat** | | | | | | |  |
| Daeschler et al 2020[63] | 97.2 | 3.61 | 18 | 96.2 | 1.57 | 12 | 70°C, 60 min, 05 cycles |
| Daeschler et al 2020[63] | 97.6 | 1.5 | 16 | 96.2 | 1.57 | 12 | 70°C, 60 min, 10 cycles |
| Liao et al 2020[58] | 96.67 | 0.65 | 3 | 96.52 | 1.37 | 30 | 75 °C, 30 min, 01 cycle |
| Liao et al 2020[58] | 97.25 | 0.34 | 3 | 96.52 | 1.37 | 30 | 75 °C, 30 min, 10 cycles |
| Pascoe et al 2020[62] | 98.19 | 0.08 | 3 | 98.35 | 0.1 | 3 | 70°C, 90 min, 01 cycle |
| Pascoe et al 2020[62] | 98.07 | 0.08 | 3 | 98.35 | 0.1 | 3 | 70°C, 90 min, 03 cycles |
| **3.4 Moist heat** | | | | | | |  |
| Anderegg et al 2020[68] | 99.12 | 8.544 | 5 | 99.29 | 8.347 | 5 | 85°C, 60-85%RH, 05 cycles |
| Daeschler et al 2020[63] | 97.0 | 0.94 | 12 | 96.2 | 1.57 | 12 | 70°C, 50% RH, 05 cycles |
| Daeschler et al 2020[63] | 96.3 | 1.89 | 12 | 96.2 | 1.57 | 12 | 70°C, 50% RH, 10 cycles |
| Liao et al 2020[58] | 95.16 | 0.73 | 3 | 96.52 | 1.37 | 30 | 100°C, 10 min, 01 cycle |
| Liao et al 2020[58] | 80.65 | 2.97 | 3 | 96.52 | 1.37 | 30 | 100°C, 10 min, 10 cycles |
| **3.5 Ethanol** | | | | | | |  |
| Grinshpun et al 2020[70] | 80.2 | 1.7 | 3 | 96.9 | 1.8 | 3 | soaking in 70% ethanol for 2 h, 01 cycle |
| Liao et al 2020[58] | 56.33 | 03.03 | 3 | 96.52 | 1.37 | 30 | 75%, immersion and air dry, 01 cycle |
| **3.6 Microwave** | | | | | | |  |
| Pascoe et al 2020[62] | 98.28 | 0.06 | 3 | 98.35 | 0.1 | 3 | MGS, 90 sec, 03 cycles |
| Pascoe et al 2020[62] | 98.21 | 0.03 | 3 | 98.35 | 0.1 | 3 | MGS+oil, 90 sec, 01 cycle |
| Pascoe et al 2020[62] | 98.19 | 0.07 | 3 | 98.35 | 0.1 | 3 | MGS+oil, 90 sec, 03 cycles |
| Pascoe et al 2020[62] | 97.36 | 0.96 | 3 | 98.35 | 0.1 | 3 | MGS 90 sec, 01 cycle |
| **3.7 Sodium hypochlorite** | | | | | | |  |
| Liao et al 2020[58] | 73.11 | 7.32 | 3 | 96.52 | 1.37 | 30 | chlorine-based (2%), light spray and air dry, 01 cycle |
| **3.8 Autoclave** | | | | | | |  |
| Grinshpun et al 2020[70] | 95.5 | 1.1 | 3 | 96.9 | 1.8 | 3 | 121ºC, 15 psi, 30 min, 01 cycle |
| Grinshpun et al 2020[70] | 96.0 | 2.1 | 3 | 96.9 | 1.8 | 3 | 121ºC, 15 psi, 30 min, 05 cycles |
| Grinshpun et al 2020[70] | 96.0 | 1.9 | 3 | 96.9 | 1.8 | 3 | soiled, 121ºC, 15 psi, 30 min, 01 cycle |
| Grinshpun et al 2020[70] | 93.0 | 1.8 | 3 | 96.9 | 1.8 | 3 | soiled, 121ºC, 15 psi, 30 min, 05 cycles |
| Harskamp et al 2020[75] | 93.43 | 1.1333 | 14 | 97.17 | 3.333 | 8 | 17 min at 121°C with 34 min total cycle time, 01 cycle |
| Harskamp et al 2020[75] | 87.3 | 1.1667 | 16 | 97.17 | 3.333 | 8 | 17 min at 121°C with 34 min total cycle time, 02 cycles |
| Harskamp et al 2020[75] | 94.4 | 1.3 | 4 | 99.3 | 0.3 | 4 | 17 min at 121°C with 34 min total cycle time, 03 cycles  Only the 3M1862 model was considered in the analysis, as it was the only one that maintained the characteristics after two test cycles. |
| **3.9 Chlorine dioxide** | | | | | | |  |
| Cai and Floyd 2020[36] | 85.65 | 2.15 | 10 | 97.0 | 0.7 | 10 | ClO₂ |
